# Functional differences between PD-1^+^ and PD-1^-^ CD4^+^ effector T cells in healthy donors and patients with glioblastoma multiforme

**DOI:** 10.1371/journal.pone.0181538

**Published:** 2017-09-07

**Authors:** Brittany A. Goods, Amanda L. Hernandez, Daniel E. Lowther, Liliana E. Lucca, Benjamin A. Lerner, Murat Gunel, Khadir Raddassi, Vlad Coric, David A. Hafler, J. Christopher Love

**Affiliations:** 1 Departments of Biological Engineering and Chemical Engineering, Koch Institute for Integrative Cancer Research, Massachusetts Institute of Technology, Cambridge, Massachusetts, United States of America; 2 Departments of Neurology and Immunobiology, Yale School of Medicine, New Haven, Connecticut, United States of America; 3 Department of Genetics, Yale School of Medicine, New Haven, Connecticut, United States of America; 4 Department of Neurosurgery, Yale School of Medicine, New Haven, Connecticut, United States of America; 5 Bristol-Myers Squibb, Wallingford, Connecticut, United States of America; 6 The Broad Institute of MIT and Harvard, Cambridge, MA, United States of America; Jackson Laboratory, UNITED STATES

## Abstract

Immune checkpoint inhibitors targeting programmed cell death protein 1 (PD-1) have been highly successful in the treatment of cancer. While PD-1 expression has been widely investigated, its role in CD4^+^ effector T cells in the setting of health and cancer remains unclear, particularly in the setting of glioblastoma multiforme (GBM), the most aggressive and common form of brain cancer. We examined the functional and molecular features of PD-1^+^CD4^+^CD25^—^CD127^+^Foxp3^—^effector cells in healthy subjects and in patients with GBM. In healthy subjects, we found that PD-1^+^CD4^+^ effector cells are dysfunctional: they do not proliferate but can secrete large quantities of IFNγ. Strikingly, blocking antibodies against PD-1 did not rescue proliferation. RNA-sequencing revealed features of exhaustion in PD-1^+^ CD4 effectors. In the context of GBM, tumors were enriched in PD-1^+^ CD4^+^ effectors that were similarly dysfunctional and unable to proliferate. Furthermore, we found enrichment of PD-1^+^TIM-3^+^ CD4^+^ effectors in tumors, suggesting that co-blockade of PD-1 and TIM-3 in GBM may be therapeutically beneficial. RNA-sequencing of blood and tumors from GBM patients revealed distinct differences between CD4^+^ effectors from both compartments with enrichment in multiple gene sets from tumor infiltrating PD-1^—^CD4^+^ effectors cells. Enrichment of these gene sets in tumor suggests a more metabolically active cell state with signaling through other co-receptors. PD-1 expression on CD4 cells identifies a dysfunctional subset refractory to rescue with PD-1 blocking antibodies, suggesting that the influence of immune checkpoint inhibitors may involve recovery of function in the PD-1^—^CD4^+^ T cell compartment. Additionally, co-blockade of PD-1 and TIM-3 in GBM may be therapeutically beneficial.

## Introduction

Glioblastoma multiforme (GBM) is the most common primary brain tumor in adults, accounting for 82% of cases of malignant gliomas [1,2]. The current standard of care for GBM is surgical resection of the tumor followed by chemotherapy and radiation, with an average survival time of 15–17 months.[3] GBM tumors are challenging to treat and recur in nearly all patients, where the 5-year survival time is 1–5% [4]. Several targeted therapies and chemotherapeutic agents have also failed to increase survival or enhance patient outcomes in GBM [5]. Treatment of GBM is further complicated by immunosuppressive mechanisms within the tumor microenvironment, resulting in dampening of T cell responses through immunosuppressive cytokine secretion and activation of immune inhibitory cascades [6,7]. Checkpoint inhibitors, or therapeutics that relieve immunosuppression, represent a promising avenue for treatment given their demonstrated effectiveness in many difficult to treat and advanced tumors such as melanoma, renal cell carcinoma, and lung cancer [8–11].

One such co-inhibitory receptor for which approved blocking antibodies exist is programmed cell death protein (PD-1). PD-1 is an inhibitory co-receptor that imparts negative feedback on effector function and it is expressed upon T cell activation and during T cell exhaustion [12]. One of its ligands (PD-L1) is diffusely expressed on tumor cells in 88% of patients with newly diagnosed GBM and 72% of patients with recurrent GBM making it an appealing co-inhibitory target for immunotherapy [13]. PD-1 is often co-expressed with other co-inhibitory receptors including the T cell immunoglobulin and mucin-domain containing-3 (TIM-3), lymphocyte-activation gene 3 (LAG3), and cytotoxic T-lymphocyte associated protein 4 (CTLA-4) [14]. The total pattern of receptor co-expression shapes overall T cell function, and has been used as a proxy for assessing the degree of exhaustion in T cells in chronic diseases such as cancer, viral infection and autoimmunity [15–17]. While CD4^+^ T cells are known to infiltrate the GBM tumor microenvironment and a significant proportion express PD-1, little is known about their influence on an immune response [18–20]. Given the fundamental role of the PD-1/PD-L1 axis in promoting T cell dysfunction [21], it is essential to delineate the molecular profiles of PD-1 expressing T cells in GBM to better understand their role in pathology.

Here, we characterized the functional and molecular signatures linked to PD-1 expression in CD4^+^ effector cells isolated from healthy subjects and from cells infiltrating GBM tumors. In healthy subjects, PD-1^+^CD4^+^CD25^—^CD127^+^Foxp3^—^effector cells have decreased proliferative ability and a transcriptional profile characterized by increased IFNγ, IL-17, and EOMES expression. Surprisingly, blocking PD-1 does not rescue proliferation of PD-1 expressing CD4^+^ effector cells. These data suggest that PD-1 may mark dysfunctional CD4^+^ effector cells, even in the peripheral blood of healthy donors. GBM tumors were enriched with PD-1^+^TIM-3^+^CD4^+^ effector cells; the percentage of which correlated with tumor grade. Like those in healthy subjects, tumor-derived PD-1^+^CD4^+^ effector cells displayed significantly less proliferation but retained the ability to produce inflammatory cytokines. Whole transcriptome analysis of healthy subjects and GBM patients confirmed the presence of distinct PD-1^+^ and PD-1^—^populations with enrichment for several gene transcriptional signatures suggestive of exhaustion in the PD-1^+^ subset. Tumor-infiltrating PD-1^—^CD4^+^ effectors also expressed many unique gene sets, including those related to metabolism. Our study suggests that high PD-1 expression on human CD4 effector cells identifies a population of exhausted effector cells that are enriched in malignant cancer and serve a crucial role in the context of inflammation and anti-tumor responses in GBM.

## Results and discussion

### PD-1^+^ CD4^+^ effector cells from healthy donors have impaired proliferative ability but retain the capacity to produce IFNγ

**Fig 1:**
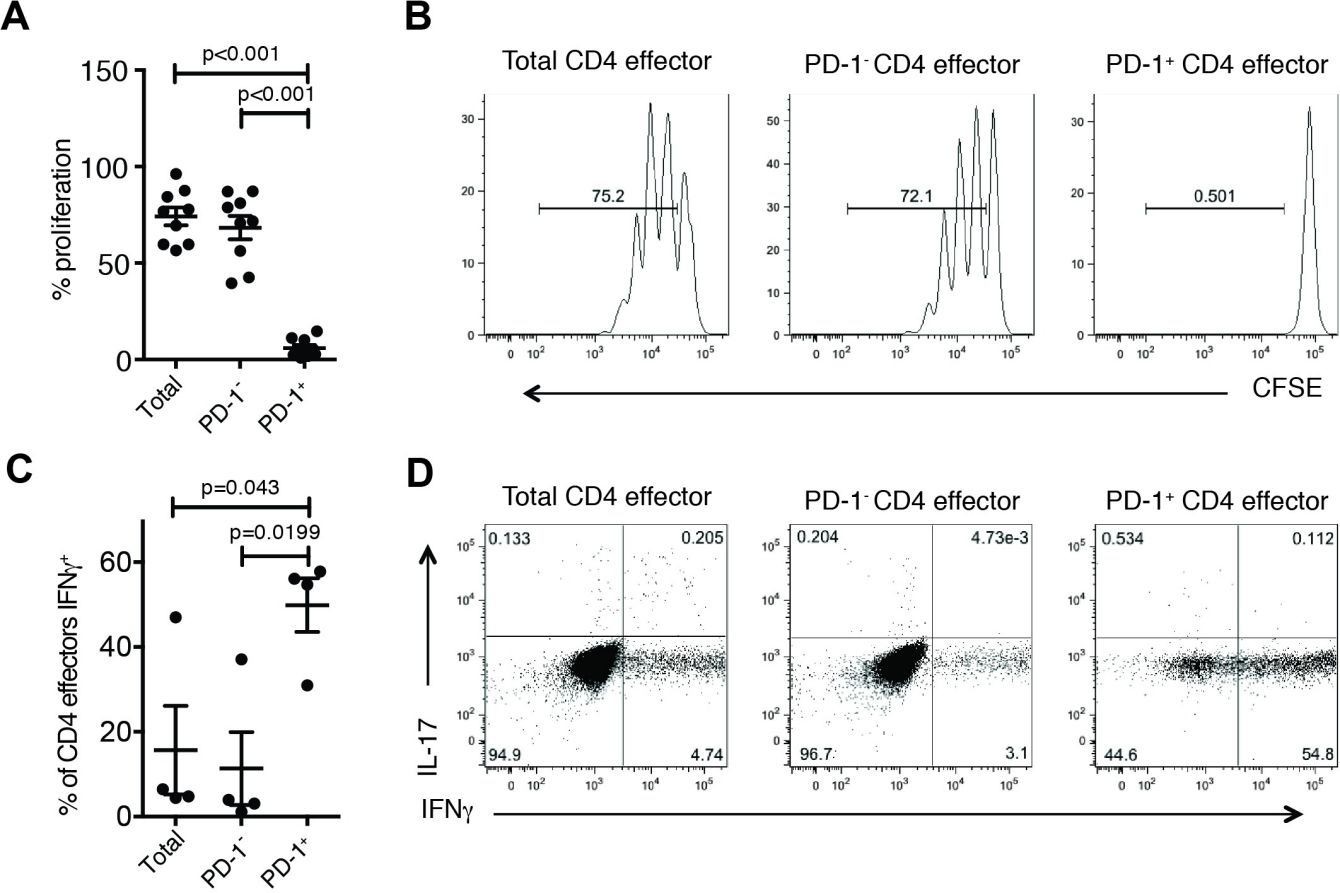
PD-1^+^ CD4 effectors demonstrate impaired proliferative ability despite competent cytokine secretion in healthy donors. CFSE labeled CD4 effectors (CD4^+^CD25^—^CD127^+^) were stimulated with αCD3/αCD28/αCD2 for 4 days. Proliferation was assessed via CFSE dilution as measured by flow cytometry. Percentage of proliferating cells is plotted (n = 9) (A) and representative plots from one experiment (B) are shown. After 4 days, cells were restimulated with PMA (50ng/ml) and Ionomycin (250ng/ml) for 4 hours in the presence of Brefeldin A (10μg/ml) and production of IFNγ and IL-17 were measure by flow cytometry. Cytokine production from CD4 effectors displayed as summary (n = 4) (C) and in representative plots (D). Statistical analyses were performed using paired student’s t-test.

We first sought to characterize the function and phenotype of PD-1^+^ and PD-1^—^populations of CD4^+^CD25^—^CD127^+^Foxp3^—^T cells (CD4 effectors) in healthy individuals to determine if PD-1^+^ marks exhausted and dysfunctional phenotypes as those previously characterized in PD-1^+^ Tregs [22]. We sorted CD4 effectors into PD-1^+^, PD-1^—^and total effector cell populations (S1 Fig) and compared entry into cell cycle by CFSE dilution after stimulation with αCD3/αCD28/αCD2. PD-1^—^CD4 effector cell proliferation was similar to the total effector population, while PD-1^+^ CD4 effectors did not enter cell cycle (Fig 1A and 1B). We confirmed that this was independent of CD45RO expression (S2 Fig). We next determined whether PD-1 expression was associated with cytokine secretion. PD-1^+^, PD-1^—^and total CD4 effectors were stimulated with αCD3/αCD28/αCD2 [23] for four days before re-stimulating with PMA and ionomycin in the presence of brefeldin A. We found significantly increased expression of the Th1-associated cytokine IFNγ in PD-1^+^ CD4 effectors compared to PD-1^—^and total CD4 effectors, but no significant increase in the Th17-associated cytokine IL-17 (Fig 1C and 1D). Our data suggests that PD-1^+^ CD4 effectors cannot enter cell cycle but, paradoxically, can secrete inflammatory cytokines.

### Proliferative ability of PD-1^+^ CD4 effector cells from healthy subjects is not rescued by PD-1 blockade or by addition of IL-2

**Fig 2:**
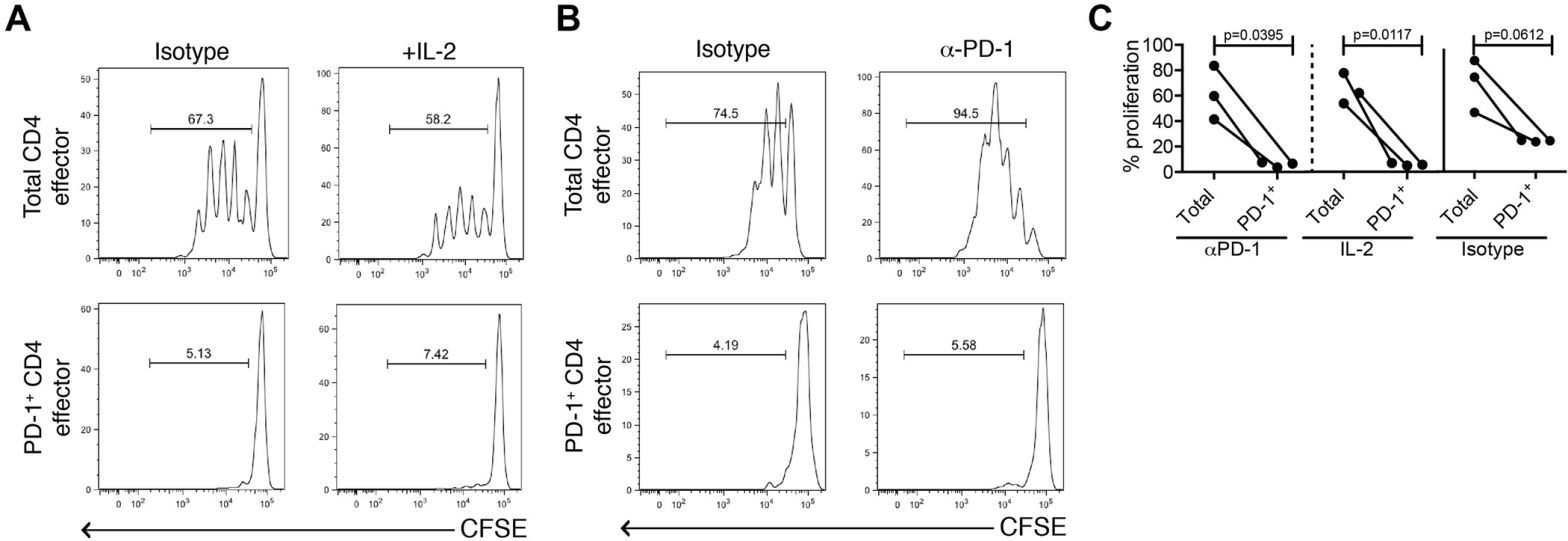
Administration of IL-2 or of PD-1 blocking antibodies does not recover proliferative function of PD-1^+^ CD4 effectors in healthy donors. CFSE labeled CD4 effectors (CD4^+^CD25^—^CD127^+^) stimulated with αCD3/αCD28/αCD2 for 4 days in the presence of either (A) IL-2 (10U/mL, n = 3) or (B) an anti-PD-1 antibody (10μg/ml, n = 3). Proliferation assessed by CFSE dilution measured by flow cytometry following 4-day incubation. One representative donor is shown for each. (C) Dot plot summary of 3 independent donors per experimental condition, isotype control on far-right panel.

It has previously been shown that blocking CD8^+^PD-1^+^ T cells isolated from healthy donors and non-small cell lung cancer patients with PD-1 antibodies partially restores T cell proliferative function and cytokine secretion [21,24]. Thus, we determined if the function of CD4 effectors from healthy donors could be recovered upon the addition of PD-1 blocking antibodies or IL-2. Here, however, the proliferative ability of PD-1^+^ CD4 effectors was not rescued by PD-1 blockade or by the addition of IL-2 (Fig 2A–2C); this effect was confirmed with isotype control. Increased proliferation was observed when blocking PD-1 antibodies were added to the total CD4 effector cell cultures. This suggests that anti-PD1 agents may exert therapeutic efficacy through PD-1^—^CD4 effectors rather than by reversing dysfunction in T cells already expressing PD-1 or through mechanisms not related to PD-1 signaling. Increased IFNγ expression by PD-1^+^CD4^+^ T cells, coupled with an inability to recover proliferative capacity even in the presence of PD-1 blocking antibodies, suggests that these cells are terminally differentiated and resistant to entry into cell cycle, as has previously been described for CD8^+^ T cells [25].

### PD-1^+^ CD4 effector cells from healthy donors have a unique transcriptional profile enriched in pro-inflammatory, exhaustive, and apoptotic signatures

We used transcriptional profiling to better understand the observed proliferative deficiency in PD-1^+^ CD4 effector cells despite retained ability to produce cytokine. PD-1^+^ and PD-1^—^CD4 effectors were sorted from healthy donors and their transcriptome profiled by RNA-sequencing. High-quality replicates were included for subsequent analysis (S1 and S2 Tables). A total of 435 genes were identified as differentially expressed between PD-1^+^ and PD-1^—^CD4 effectors (S3 Table), where *IFNG*, *CTLA-4*, and *LAG-3* were significantly upregulated in PD-1^+^ CD4 effectors (S3A Fig**)**. Functional classification using DAVID [26] revealed unique gene modules in PD-1^+^ CD4 effectors, including enrichment of activation, apoptotic, and immune system pathways (S3B Fig). The gene module containing PD-1 (*PDCD1*) also included surface markers and co-receptors, including integrins and TNF-family members (S4 Table). Conversely, there were only two modules enriched in PD-1^-^ CD4 effectors, one containing surface markers and the other several transcription factors (S5 Table). Taken together, these data suggest that in healthy donors PD-1^+^ CD4 effectors are distinct from their PD-1^—^counterparts; they co-express many inhibitory co-receptors and unique surface receptors, and may be apoptotic.

**Fig 3:**
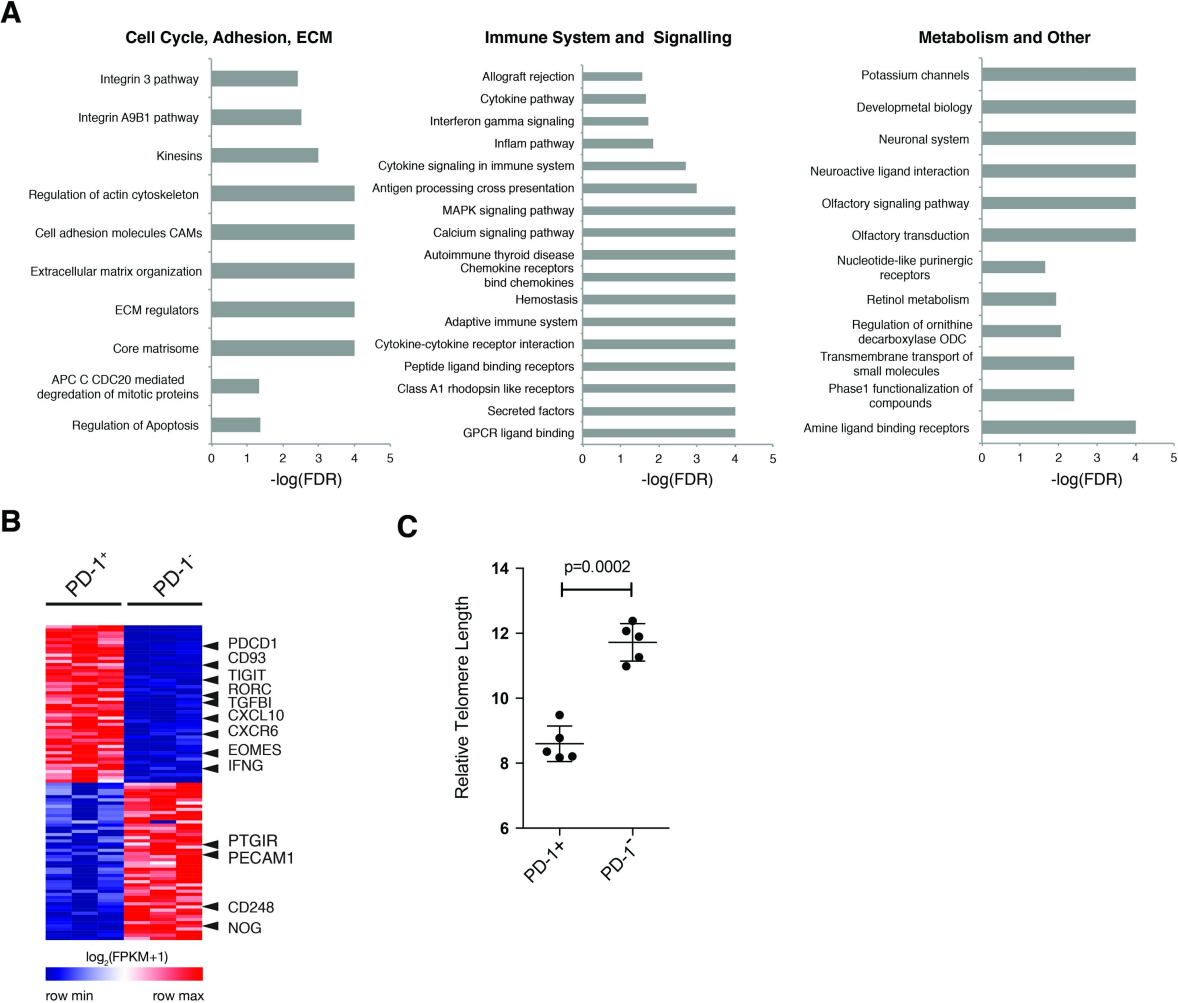
Transcriptional profiling of PD-1^+^ CD4 effector cells from healthy donors confirms enrichment in pro-inflammatory and exhaustion signatures. (A) Gene set enrichment analysis was performed on PD-1^+^ and PD-1^—^CD4 effector cells isolated from healthy donors (n = 3). Forest plots show enrichment of gene sets from each indicated functional category in PD-1^+^ cells. (B) Top features by rank in PD-1^+^ or PD-1^—^subsets are shown, with selected markers highlighted. (C) Telomere length assessed in *ex-vivo* sorted CD4 effector (CD4^+^CD25^—^CD127^+^) cells using Telomere PNA Kit for flow cytometry (Dako) (n = 5). Statistical analyses were performed using paired student’s t-test.

Next we performed gene set enrichment analysis to identify pathways that may account for functional differences between the two cell populations (27). We found significant enrichment (FDR < 0.05) of 83 pathways in PD1^+^ cells and 73 in PD-1^—^cells (S5 Table). Several pathways related to extracellular matrix and adhesion, immune system signaling, and metabolism were enriched in PD-1^+^ cells (Fig 3A). Examining the top 50 features scored by GSEA, we also found molecules involved in IFNγ and IL-17 production (Fig 3B), confirming our observed functional data. Interestingly, we did not see enrichment in PD-1 signaling or regulation of PD-1 signaling in PD-1^+^ CD4 effectors. We confirmed differential protein expression of several surface markers by flow cytometry, which highlighted changes in CXCR5, LAG-3, and CTLA-4 (S4 Fig). We also found an increase in Annexin V expression in the PD-1^+^ compartment, which taken with our GSEA data, may suggest a sub-population of apoptotic cells. This alongside the presence of the transcription factor *EOMES* and multiple co-inhibitory receptors suggests that PD-1^+^ expression in CD4^+^ T cells identifies an exhausted population of cells [27].

Given that we did not find enrichment in cell cycle or observe proliferation of PD-1^+^ CD4 effectors, we wished to determine their differentiation state. Length of telomeres can indicate previous cell divisions; telomere shortening can induce replicative senescence and suggest impaired function [28]. We observed significantly shorter telomeres in the PD-1^+^ as compared to the PD-1^—^population, suggesting that PD-1^+^ CD4 effectors are likely terminally differentiated (Fig 3C). Thus, PD-1^+^ CD4 effectors are replicatively exhausted and apoptotic, despite the retained ability to produce cytokines. Expression of integrins and general immune activation gene sets suggest that PD-1 likely marks tissue-experienced and activated T cells. Thus, PD-1-expressing T cells may serve as a cellular window into immune and tissue, and longitudinal characterization of PD-1 expressing T cells may yield insight into the immune state of an individual. PD-1^+^ CD4 effectors from healthy donors are functionally distinct and our data suggests that, in addition to its role in exhaustion, expression of PD-1 on CD4 effectors characterizes a subset of CD4^+^ T cells that are unable to recover from exhaustion and dysfunction.

### Glioblastoma multiforme tumors are enriched in PD-1^+^Tim-3^+^ CD4 effector cells

PD-1 is a promising target for GBM therapy because tumors express high amounts of PD-L1 and expression of PD-1 on blood-derived T cells has been correlated with poor clinical outcome [29]. Since tumor-infiltrating CD4^+^ T cells can express PD-1, we sought to investigate the phenotype of PD-1^+^ CD4 effectors infiltrating gliomas as compared to those isolated from the blood of patients or healthy donors. TIM-3 is a negative regulator of T cell activation and is also involved in exhaustion, Th1 responses, and response to viral infections [30]. Our previous work has shown that TIM-3 co-expression with PD-1 marks dysfunctional Tregs in glioblastoma [22]. Therefore we also sought to characterize TIM-3 expression on CD4 effectors from GBM patients.

**Fig 4:**
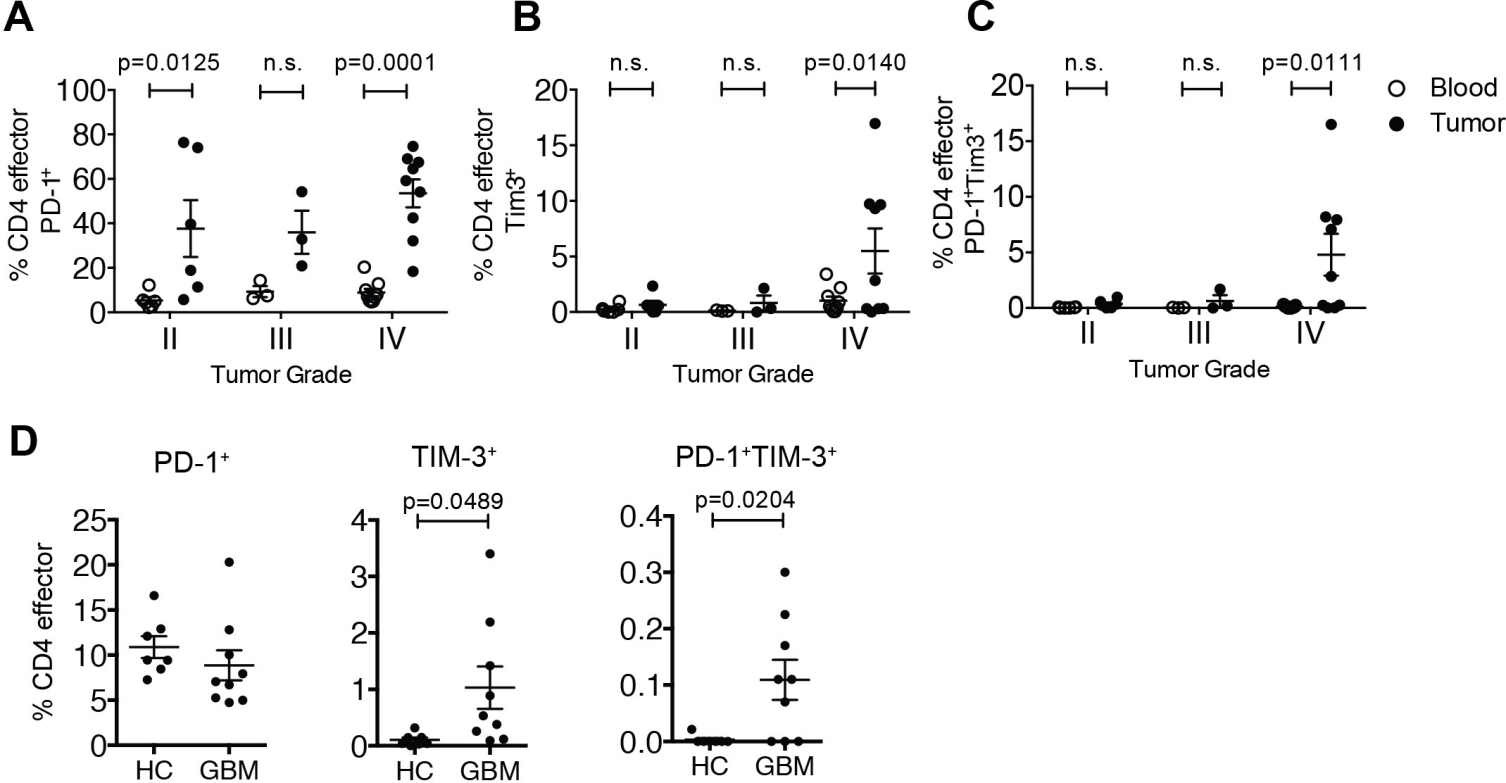
Enrichment of PD-1 and TIM-3 on CD4^+^ effector cells isolated from GBM tumors. CD4 effector cells were isolated by flow cytometric sorting from healthy donors and patients with glioblastoma multiforme (GBM). (A) Presence of PD-1^+^ (A), TIM-3^+^ (B) and PD-1^+^TIM-3^+^ CD4 effectors (CD4^+^CD25^—^CD127^+^) (C) within glioblastoma tumors (black) and matched blood (white) as a function of tumor grade (n = 6 for II, n = 3 for III, and n = 9 for IV). Mean + S.D., Two-way ANOVA with Sidak’s multiple comparisons test. (D) Comparison of frequencies of PD-1^+^, TIM-3^+^ and PD-1^+^TIM-3^+^ CD4 effectors (CD4^+^CD25^—^CD127^+^) within peripheral blood of healthy controls (n = 7) compared to patients with glioblastoma. Statistical analyses were performed using student’s t-test.

PD-1^+^ and PD-1^—^CD4 effectors were isolated from fresh tumor resections and matched peripheral blood from patients with GBM (S6 Table). We observed up-regulation of PD-1 on the cell surface of CD4 effectors infiltrating gliomas of all WHO tumor grades, with a statistically significant increase in GBM tumors **(**Fig 4A, Grade IV**)** [31].

We also found an increase in the percentage of TIM-3^+^ CD4 effectors from GBM (WHO Grade IV) **(**Fig 4B), as well as an enrichment of PD-1^+^TIM-3^+^ CD4 effectors relative to peripheral blood **(**Fig 4C**).** We found comparable expression of PD-1 on CD4 effector cells from the blood of patients and healthy donors (Fig 4D). Interestingly, there were no TIM-3^+^ CD4 effectors in the circulation of healthy controls, and conversely a significant number of circulating CD4 effectors expressing both TIM-3 and PD-1 in patients with GBM (Fig 4D). This is suggestive of a highly exhausted population of T cells present in the periphery of patients with GBM.

Overall, we found increased frequencies of PD-1^+^TIM-3^+^ CD4 effector cells in blood and tumors of GBM patients as compared to healthy donors, suggesting a population of tumor-experienced cells. Previous work has linked PD-1 and TIM-3 expression with T cell exhaustion and dysfunction in cancer [32], and coupled with our data, suggests that blocking TIM-3 in conjunction with PD-1 may help mitigate apoptosis and boost effector function of a large fraction of T cells within the GBM tumor environment. Recent work in the context of melanoma also suggests that expression of PD-1 marks tumor experienced cells [20]. Given that we found TIM-3^+^ effectors in glioblastoma patients but not healthy donors, PD-1^+^TIM-3^+^ effectors may also represent a tumor-experienced population of T cells detectable in circulation. We also found that about half of the patients analyzed showed a more drastic increase in the percentage of TIM-3^+^ and PD-1^+^ effectors. This suggests that study of these cells in a larger cohort of patients could reveal a method for patient stratification. In sum, our data suggests that further study of this rare subpopulation of T cells is needed in the context of GBM.

### CD4 effector cells isolated from patients with glioblastoma multiforme have impaired proliferation but retain effector function

**Fig 5:**
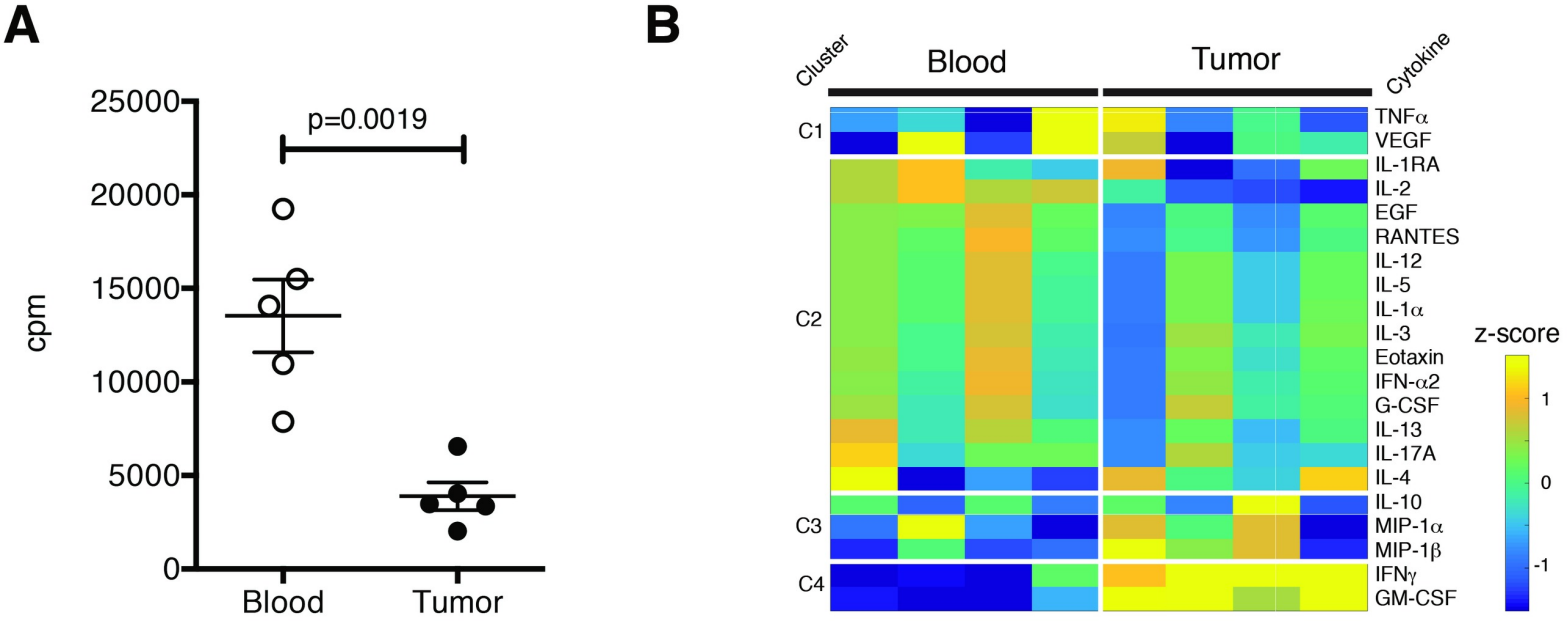
CD4 effector cells isolated from GBM tumors are associated with poor proliferation and production of IFNγ and GM-CSF. (A) Proliferation of peripheral blood or tumor-infiltrating CD4 effectors (CD4^+^CD25^—^CD127^+^) from patients with glioblastoma multiforme (GBM) (n = 5) as measured by thymidine incorporation. (B) Tumor (black) and peripheral blood (white) derived GBM patient CD4 effectors (CD4^+^CD25^—^CD127^+^) were sorted and stimulated with PMA (50ng/ml) and Ionomycin (250ng/ml) for 4 hours (n = 4). Cytokine release measured by Luminex analysis. Statistical analyses were performed using paired student’s t-test.

To determine the functionality of tumor infiltrating CD4 effector cells as compared to blood, we isolated CD4 effector cells from GBM tumors and peripheral blood, stimulated them with αCD3/αCD28/αCD2, and assessed proliferation. Tumor-derived CD4 effectors proliferated significantly less than those from the peripheral blood (Fig 5A**)**, similar to our observations from healthy donors. To ascertain whether these CD4 effectors also retained the ability to produce cytokines, tumor-infiltrating and peripheral blood-derived CD4 effectors were stimulated with PMA and ionomycin for four hours, the supernatants were collected, and analyzed by Luminex (S5 Fig). We found that tumor-derived CD4 effector cells retained the ability to produce inflammatory cytokines, including the pro-inflammatory cytokines IFNγ and GM-CSF.

These data suggest the presence of mixed populations of effector cells capable of secreting cytokines of the Th1, Th2 or Tr1 lineages as compared to matched CD4 cells from the peripheral blood. T cells from tumors may secrete high levels of IFNγ to recruit additional CD4 effector cell help to the CNS in an effort to replenish an exhausted store that is likely unable to perform supportive functions for CD8 T cells, since it has been shown that IFNγ can promote migration of T cells to the CNS [33]. Previous work has also found that exhaustion and dysfunction can be induced in CD4 T cells in an IFNγ dependent manner, suggesting that tumor-derived factors such as secretion of IL-12 from monocytes and macrophages or IFNγ secretion could also contribute to T cell dysfunction in a paracrine manner [34]. Our data indicate that tumor-derived CD4 effectors are capable of secreting cytokine, including IFNγ; further work at the single-cell level or profiling of secretion from broader populations of infiltrating immune cells in concert with tumor-cells is needed to determine potential local drivers of such dysfunction.

### CD4 effector cells from blood and tumors of GBM patients are transcriptionally distinct and tumor-derived PD-1^—^Teff are enriched in many gene sets

The expression of PD-1 on T cells isolated from the blood of glioblastoma patients has been studied previously, however, few have compared molecular profiles to those derived from tumors [29,35]. We performed transcriptional profiling to determine if PD-1^+^ CD4 effector cells from the blood of GBM patients differed from those found in tumors. PD-1^+^ and PD-1^—^CD4 effectors were isolated from blood and tumors of GBM patients and transcriptionally profiled by RNA-sequencing. High quality replicates were selected on the basis of sequencing metrics (S1 Table) and expression of housekeeping genes for subsequent analysis (S2 Table). Principal components analysis (PCA) revealed that tumor-derived cells, regardless of PD-1 status, were transcriptionally distinct from their GBM blood or healthy donor blood derived counterparts (S6 Fig). The increased spread in tumor samples as compared to blood indicates a high degree of heterogeneity between patient tumors, even at a bulk level, a finding also observed in the setting of melanoma [36].

We then performed GSEA in order to compare enriched gene sets from all patients and healthy donors. We compared the top 100 and bottom 100 ranked genes found in PD-1^+^ versus PD-1^—^T cells from GBM patient blood, tumors, and blood from healthy donors (S7A Fig). Very few genes were common among all compartments (S7B Fig). This result agrees with previous transcriptional data showing distinct differences between blood of GBM patients and healthy donors, and further suggests there are key differences between the blood and tumor compartments [35]. We found that *GITR* (tumor necrosis factor receptor superfamily, member 18) was one of the shared up-regulated genes in PD-1^+^ CD4 effectors across compartments, which could suggest a common role in the program of PD-1^+^ CD4 effector cells. GITR is a co-stimulatory molecule responsible for maintaining self-tolerance; when blocked it can induce autoimmunity [37,38]. CD4 effectors thus may up-regulate GITR to prevent anergy and maintain effector functions in blood and tumors, and future studies should investigate the specific role of GITR in the context of immune-privileged tumor sites like GBM.

**Fig 6:**
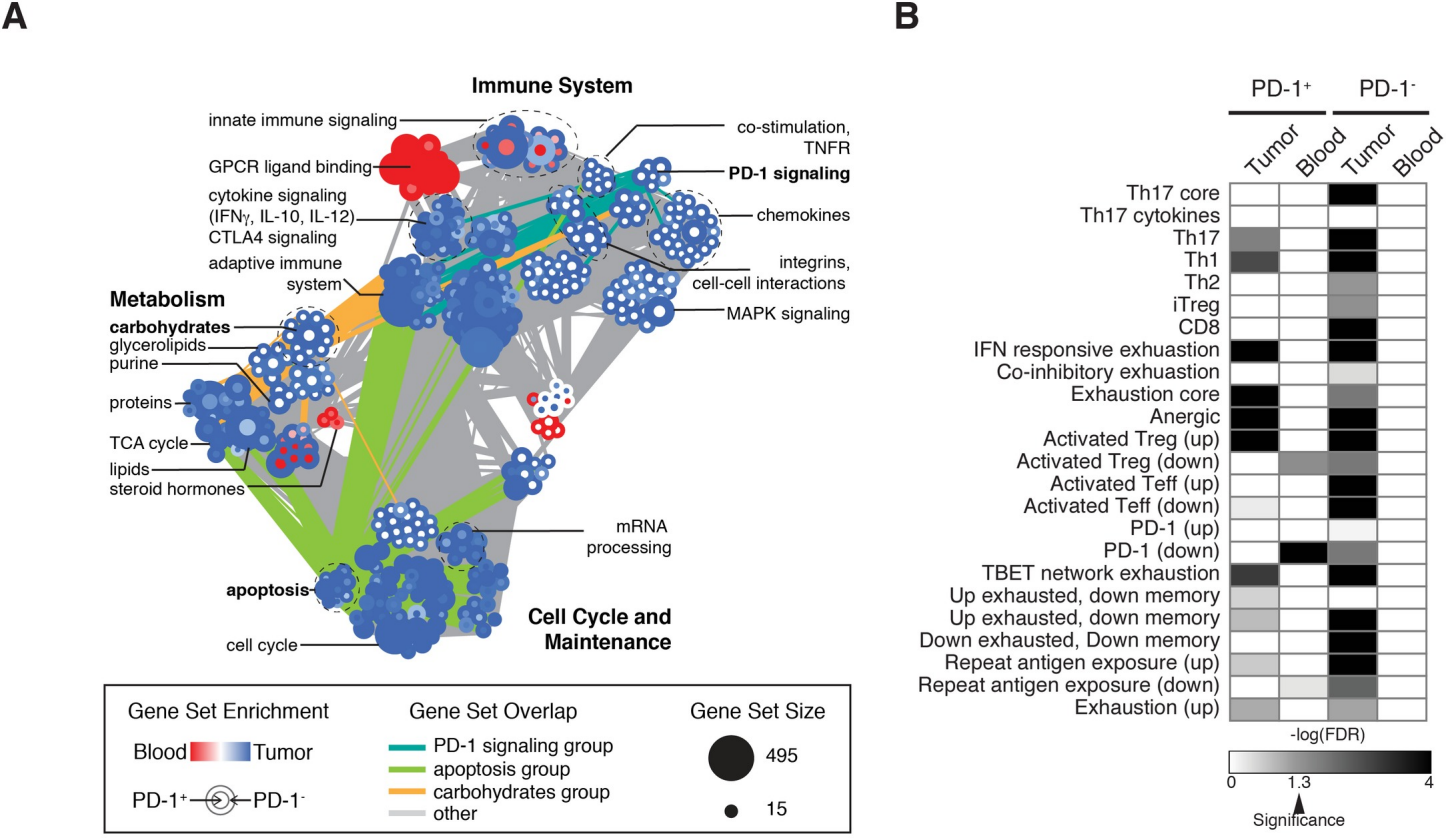
Transcriptional profiling of PD-1^+^ and PD-1^—^CD4 effectors from tumors and blood of GBM patients reveals unique signatures of metabolism in the PD-1^—^compartment and overall exhaustion in tumors. (A) Enrichment map of GSEA results comparing tumors and blood in the PD-1^+^ or PD-1^—^compartment of GBM patients. Gene sets with a FDR < 0.01 are shown as nodes, edges represent gene set overlap, color of the node represents blood (red) or tumor (blue) and PD-1^+^ is inside and PD-1^—^is outside. (B) Heatmap of log(FDR) from GSEA results comparing tumor to blood within each compartment for curated gene sets described in S7 Table. Significance (FDR < 0.05) is indicated on the color bar.

To test the hypothesis that CD4 effectors from tumors are overall more impaired that their counterparts in blood, regardless of PD-1 status, we performed GSEA using selected replicates (S2 Table) and compared PD-1^+^ or PD-1^—^CD4 effectors from blood as compared to tumors. We created an enrichment map to visualize the most highly significant gene sets (FDR < 0.01) (Fig 6A). Many gene sets were enriched in tumor CD4 effectors, regardless of PD-1 status, including several cell cycle and maintenance gene sets (apoptosis, mRNA processing, and cell cycle) and immune-related gene sets (cytokine signaling, CTLA4 signaling, and adaptive immunity). Previous work has established that T cells isolated from GBM are apoptotic [39]. Interestingly, we found enrichment of several metabolic gene sets, including lipid, protein and TCA cycle genes. Conversely, there were only two groups of genes enriched in blood regardless of PD-1 status, including one related to steroid hormone metabolism. Surprisingly, we found a high number of gene sets enriched in tumors in the PD-1^—^compartment only. These include immune-related gene sets (co-stimulation, PD-1 signaling, and several chemokines), as well as a group of gene sets related to carbohydrate and glycerolipid metabolism. Additionally, gene sets in the PD-1 signaling group also have many connections to other immune groupings, including carbohydrate and glycerolipid metabolism. The role of metabolism in T cell activation and function has emerged as a potential therapeutic avenue in addition to checkpoint blockade in cancer treatment [40]. Although an admittedly small sampling of tumors, our data indicate that PD-1^—^CD4 effectors may be overall more metabolically active through the glycolytic and amino acid programs that are necessary to support effector T cell function.

Finally, given that we found enrichment in anergic and exhaustion marker signatures in healthy donors, apoptotic and inhibitory signaling in GBM patients, but retained ability to produce cytokines of Th1, Th2, or Tr1 lineages we sought to better determine the relative contribution of these T cell states to each compartment. Using a previously described set of known exhaustion, Th-specific, memory, and activation gene signatures curated from the literature (S7 Table), we used GSEA to compare tumor to blood for the PD-1^+^ and PD-1^—^T cells from each compartment (Fig 6B). We found that tumor-derived T cells, regardless of PD-1 status, were enriched in many gene sets, including Th17, Th1 and several exhaustion as compared to blood, further supporting the compartment specific differences discussed above.

Taken together, tumor-derived CD4 effectors are transcriptionally different from their blood-derived counterparts. CD4 effectors from tumors are enriched in many gene sets as compared to blood, including those that suggest PD-1^—^CD4 effectors are more metabolically active and enriched in immune co-stimulation gene sets. Our transcriptional data indicates that PD-1^—^CD4 effectors from tumors may be attempting to mount an immune response while PD-1^+^ CD4 effectors from tumors may be functionally impaired. Given the large spread in our transcriptional data set and high-degree of heterogeneity, future work should use single-cell approaches to determine the full resolution of T cell states, both transcriptionally and metabolically, in GBM.

## Conclusion

Defining the functional immunological state of PD-1 expressing T cells in the context of health and cancer is essential to better understanding the efficacy of immune checkpoint blockade. Previous studies have highlighted the role of PD-1 expression on CD8 and regulatory T cells in chronic infection and cancer, but few studies have investigated PD-1^+^ CD4 effector T cells. Here, we characterized the role of PD-1 expression on CD4^+^ T cells from healthy donors and in the context of GBM.

We found that that PD-1 may identify CD4 effector cells that are irreversibly dysfunctional as shown by their cytokine production and decreased proliferation that is not rescued by PD-1 blockade. Transcriptionally, PD-1^+^ CD4 effectors from healthy donors express co-inhibitory markers and are enriched in several gene sets, including apoptosis, extracellular matrix gene sets, immune signaling pathways, and activation gene sets. This suggests that PD-1 expression denotes a population that is functionally distinct, tissue-experienced, activated, and likely unable to recover from exhaustion. In the context of GBM, we found that tumors have a high degree of T cell infiltration and CD4 effectors isolated from tumors are able to produce cytokines, including IFNγ as was observed in our data from healthy subjects. Through transcriptional analysis, we found that *GITR* was one of the few genes commonly up-regulated in PD-1^+^ CD4 effectors in health and GBM. This finding may suggest that CD4 effectors up-regulate GITR as an attempt to prevent anergy and maintain effector functions in both blood and tumors (*46*). Interestingly, we found a number of gene sets enriched in PD-1^—^CD4 effectors isolated from tumors of GBM patients. These included immune-related gene sets (co-stimulation, PD-1 signaling, and several chemokines), as well as gene sets related to carbohydrate and glycerolipid metabolism. The role of metabolism in T cell activation and function has emerged as a potential therapeutic avenue in addition to checkpoint blockade in cancer treatment (*48*). Although a small sampling of tumors, our data indicate that PD-1^—^CD4 effectors may be overall more metabolically active through glycolytic and amino acid programs that are necessary to support CD4 effector T cell function. Given the large spread in our transcriptional data set and high-degree of heterogeneity, future work should use single-cell approaches to determine the full resolution of T cell states, both transcriptionally and metabolically, in GBM.

Finally, we found an increase in the percentage of TIM-3 and PD-1 co-expressing T cells in tumors of GBM patients and their blood, but not in healthy donors. This may suggest that this rare population of cells is tumor experienced, and may further indicate that blockade of PD-1 and TIM-3 may be beneficial in the context of GBM immunotherapy. This also suggests that the immune functions of TIM-3 and PD-1 co-expressing cells should be investigated further in the context of GBM. In the context of treatment with anti-PD-1 antibodies, our data suggests that treatment may affect PD-1^—^CD4 effectors but not PD-1^+^ CD4 effectors. Clinically, this suggests that treatment with anti-PD-1 immunotherapy could be greatly enhanced by combining anti-PD-1 with T cell trafficking agents to promote greater T cell infiltration into the tumor microenvironment to aid tumor resident PD-1^—^and PD-1^intermediate^ CD4 effectors perform supportive functions.

## Materials and methods

### Study design and approval

All patients with brain tumors and healthy donors provided samples (human tissue and/or blood) after informed written consent and approval by the Institutional Review Board at the Yale School of Medicine. Patients were recruited between 2012 and 2014 on the basis of presentation with symptoms associated with glioblastoma. Patients with recurrent glioblastoma were also recruited. Baseline demographic data was recorded for each patient and is summarized in S6 Table. Tumors were histologically characterized by the Pathology Department at the Yale-New Haven Hospital according to the 2007 WHO classification 59 and immunohistochemical Ki-67 staining performed to measure the proliferative index of each tumor. Immune monitoring samples were taken from patients as part of the CheckMate 143 clinical trial (ClinicalTrials.gov Identifier:NCT02017717) of Nivolumab for recurrent GBM.

### Flow cytometry

For cellular isolation and immunophenotyping analyses, flow cytometry was performed using a BD LSRFortessa for analysis and a BD FACS Aria II for cell sorting. All analysis was carried out using FlowJo software (Treestar Inc). Cell viability was assessed using Live/Dead Cell Viability Assays (Life technologies). The following anti-human surface and intracellular antibodies and dyes were utilized: anti-CD4 (RPA-T4), anti-CD8 (RPA-T8), anti-CD25 (M-A251), anti-CD127 (HIL-7R-M21), anti-PD-1 (EH12.1), anti-PDL1 (MIH1) anti-IFNγ(B27), anti-KI67 (B56), anti-CCR6 (11A9), anti-CXCR3 (IC6), anti-CCR7 (3D12), anti-CXCR5 (RF8B2), V500 Annexin-V stain (all BD Biosciences); anti-TIM-3 (F38-2E2), anti-CTLA-4 (L3D10), anti-CD226 (TX25), anti-IL-10 (JES3-9D7) (all from Biolegend), anti-Foxp3 (PCH101), anti-IL-2 (MQ1-17H12), anti-ICOS (ISA3) (all from eBioscience), anti-LAG-3 (polyclonal goat IgG, R&D systems), anti-phosphoSer319 FoxO1 (polyclonal rabbit IgG, Bioss).

### Tumor infiltrating lymphocyte isolation

For extraction of infiltrating lymphocyte populations, freshly resected tumor specimens were manually disrupted, digested with collagenase IV (2.5 mg/ml) and DNase I (0.2 mg/ml) (Worthington Biochemical Corporation) for 1 hour, then passed through a 70mm cell strainer prior to separation on discontinuous 70–30% Percoll (Sigma Aldrich) gradients. Matched fresh PBMCs were isolated by Ficoll hypaque gradient centrifugation.

### Cell-Trace CFSE proliferation assays

CD4 effector cells (CD4^+^CD25^-^CD127^+^) were isolated via FACS sorting from healthy donors. To assess proliferative capacity, CD4 effector cells were further sorted based upon PD-1 surface expression in PD-1^+^ and PD-1^—^populations, alongside a total population that was not sorted by PD-1 expression. CD4 effector cells were stained with CellTrace CFSE (1μM, Life technologies) to assess proliferation through dilution of the dye. 6 x 10^3^ CD4 effector T cells were cultured for 4 days before assessing for viability and proliferation on a BD LSRFortessa. As indicated, IL-2 was added at 10U/mL to the culture media. Samples were further analyzed using FlowJo software.

### Blocking antibodies

Human anti-PD-1 (M3, Mouse IgG1) were a generous gift from Dr. Leiping Chen (Yale School of Medicine, New Haven) and were used at a concentration of 5mg/ml with appropriate isotype control antibody.

### Telomere length

Telomere length of sorted populations of CD4^+^CD25^—^CD127^+^PD1^+^ and CD4^+^CD25^—^CD127^+^PD1^—^was assessed using the Telomere Peptide Nuclear Antigen kit for flow cytometry (Dako). Relative telomere length was calculated as the ratio between the telomere signal from each sorted lymphocyte population and the 1301 control cell line (Sigma Aldrich).

### Thymidine incorporation

To assess growth potential of lymphocytes, CD4 effector cells (CD4^+^CD25^-^CD127^+^) were isolated via FACS sorting from blood and tumors of patients with GBM. Cells were cultured in the presence of 2 Treg Inspector Beads (anti-CD2/anti-CD3/anti-CD28) (Miltenyi) per cell. No fewer than 2000 cells were cultured. After 72 hours cells were pulsed with 0.5uCi 3H Thymidine and harvested 18 hours later. 3H-thymidine incorporation was measured using a micro beta counter.

### Luminex

Multiplex assays for cytokine secretion were performed on cell culture supernatants after stimulation with PMA (50ng/ml) and Ionomycin (250ng/ml) using Milliplex Human Cytokine/Chemokine Magnetic Bead Panel (Millipore) and analyzed as described in supplementary materials.

### Luminex data analysis

Data were analyzed using custom MATLAB scripts. Briefly, data were z-score normalized and included for analysis based on pearson correlation scores. Data were then hierarchically clustered.

### RNA-sequencing

T cells from healthy controls and GBM patients were sorted into RNA lysis buffer for RNA sequencing as described previously [41]. Briefly, RNA was extracted using the NucleoSpin RNA XS Kit (Macherey-Nagel) according to the manufacturers instructions. cDNA synthesis and amplification were performed using SMARTer Ultra Low Input RNA for Illumina Sequencing High Volume Kit (Clontech) according to the manufacturers instructions, with the following for LD amplification: [98°C for 10s, 65°C for 30s, 68°C for 3min]x18, 72°C for 10min. cDNA was normalized post quantification using Picogreen (Invitrogen) to an input of 0.5ng total for preparing sequencing libraries. Paired-end sequencing libraries were prepared using the Nextera XT DNA sample Prep Kit (Illumina) according to the manufacturers instructions. Libraries were pooled in an equimolar ratio and sequenced on a HiSeq 2500 sequencer with 200 cycles per lane (Illumina). In depth analysis methods are discussed in the Supplementary Information. All sequencing data are available through DbGaP (#18460).

Reads were mapped against UCSC’s known genes annotation of the hg19 human genome assembly using RSEM v 1.2.15 and bowtie 1.0.1 (parameters -p 4—output-genome-bam—calc-ci—paired-end—bowtie-chunkmbs 1024).[42] Posterior estimates of counts per genes were retrieved for processing in the R statistical environment v. 3.1.1, using the DESeq package.[43] After normalization (estimateSizeFactors) and variance estimates across all samples using the estimateDispersions function similar to its original implementation (sample-blind estimate and fit-only sharing mode), differential expression was performed either pairwise between every combinations of sample labels (nbinomTest) or by building a GLM model to test every condition independently (fitNbinomGLMs). P-values were adjusted using the Benjamin-Hochberg procedure (p.adjust).

Quality control was performed on forward and reverse fastq files from each sample using custom python scripts. QC metrics used were number and percentage of mapped reads, reads mapping to exonic, intronic, CDS, 5’ and 3’ untranslated regions, intergenic regions and different classes of non-coding RNAs. Additional metrics reflecting the mRNA enrichment were derived from exonic/intronic read density ratios, and biases in RNA integrity were detected using the 5’/3’ ratios. Samples kept for down stream analysis are shown in S1 Table. The log2FPKM values for mapped transcripts in each sample were used to create the input.gct file for Gene Set Enrichment Analysis (GSEA) (v17.0). GSEA (classic scoring scheme, signal to noise ranking, 15 gene set size minimum, 1000 permutations) was performed using C2CP gene sets and custom gene sets curated from the literature. Data were further analyzed using DAVID for functional classification. GeneE data visualization tool (http://www.broadinstitute.org/cancer/software/GENE-E/index.html) was used to create heatmaps of selected genes.

Enrichment maps of GSEA data were created using Enrichment Map in Cytoscape (v3.3.0) with a p-value cutoff of 0.005, FDR Q-value cutoff of 0.01, and an overlap coefficient of 0.5, and a combined constant of 0.5. To investigate connections between interesting gene sets, we highlighted connections from carbohydrate signaling (unique to PD-1^—^tumors), PD-1 signaling (unique to PD-1^—^tumors), and apoptosis (common to tumors regardless of PD-1 status).

### Statistics

Statistical analysis was carried out using GraphPad Prism (GraphPad Inc). Paired and unpaired two tailed students t tests were used when appropriate. P<0.05 was considered statistically significant.

## Supporting information

S1 Fig Gating strategy for the isolation of PD-1^+^ CD4 effector cells. Representative plot showing gates for CD4 effectors CD4^+^CD25^—^CD127^+^) and (Tregs (CD4^+^CD25^hi^CD127^lo^) among total CD4^+^ cells based on isotype control and on PD-1 stain.
(PDF)

S2 Fig Proliferative impairment of PD-1^+^ CD4 effectors independent of CD45RO expression. CFSE labeled CD4 effectors (CD4+CD25—CD127+) stimulated with αCD3/αCD28/αCD2 for 4 days. Proliferation assessed by CFSE dilution measured by flow cytometry. Representative plots displayed from 1 of 5 independent experiments.
(PDF)

S3 Fig Differential expression analysis of PD-1^+^ and PD-1^—^CD4 effectors from healthy donors. (a) Volcano plot of differentially expressed genes with select genes with p_adj_ values > 0.05 highlighted in pink and select genes annotated. (b) Forest plot of p-values of top GO enrichment analysis terms of genes up in PD-1^+^ CD4 effectors. Data from 3 healthy donors.
(PDF)

S4 Fig Characterization of surface markers on PD-1^+^ and PD-1^−^ CD4 effectors. Expression of selected cell markers within PD-1^+^ and PD-1^−^ CD4 effector (CD4^+^CD25^—^CD127^+^) populations (n = 25 total). The percent of cells expressing several markers were significantly different, including CD27 (p = 0.0006), CD45RA (p = 0.0037), CD45RO (p = 0.0032), CD57 (p = 0.0004), CD62L (p = 0.0009), CCR6 (p = 0.0059), CCR7 (p = 0.0021), ICOS (p = 0.0101), PDL1 (p = 0.0061), and LAG3 (p = 0.0008) were all significantly different, with p values indicated by paired students t test.
(PDF)

S5 Fig Analysis of Luminex data from blood and tumor derived CD4 effectors from GBM patients. (a) Box plots of z-score normalized luminex measurements for each patient. (b) Correlation matrix of all data from tumors and blood.
(PDF)

S6 Fig Principal components analysis (PCA) of transcriptional data from all patients. (a) Principal components analysis of transcriptional data (log_2_(FPKM+1)>0.01) from all samples analyzed. Data points are labeled from glioblastoma (GBM) blood, tumor, or healthy blood. (b) Percent variance accounted for in each component.
(PDF)

S7 Fig Comparison of GSEA results from healthy donors, GBM tumors and GBM blood. (a) Heatmaps of the top 50 features identified by GSEA are shown for all samples analyzed from PD-1^+^ and PD-1^—^CD4 effectors. (b) Venn diagram comparisons of features enriched in PD-1^+^ (top) or PD-1^—^(bottom) CD4 effectors. Members of several overlaps are annotated.
(PDF)

S1 Table Transcriptional data and sequencing metrics. (PDF)

S2 Table Selected housekeeping genes used for quality control of transcriptional data. (PDF)

S3 Table Differentially expressed genes for PD-1^+^ versus PD-1^—^CD4 effectors from healthy donors. (PDF)

S4 Table DAVID gene classification for PD-1^+^ healthy donors. Enrichment scores are shown for each group.
(PDF)

S5 Table Gene set enrichment results for PD1 positive and negative Teff from healthy donors (FDR<0.05). (PDF)

S6 Table Data for patients used in this study. (PDF)

S7 Table Curated exhaustion and T cell specific gene signatures from the literature. (PDF)
