# Supplementary material for: Functional differences between PD-1+ and PD-1- CD4+ effector T cells in healthy donors and patients with glioblastoma multiforme
Source: PLoS One. 2017 Sep 7;12(9):e0181538. doi: 10.1371/journal.pone.0181538 (PMC5589094; doi:10.1371/journal.pone.0181538)
Supplement: S1 Fig — Representative plot showing gates for CD4 effectors CD4+CD25—CD127+) and (Tregs (CD4+CD25hiCD127lo) among total CD4+ cells based on isotype control and on PD-1 stain. (PDF) [file pone.0181538.s001.pdf]

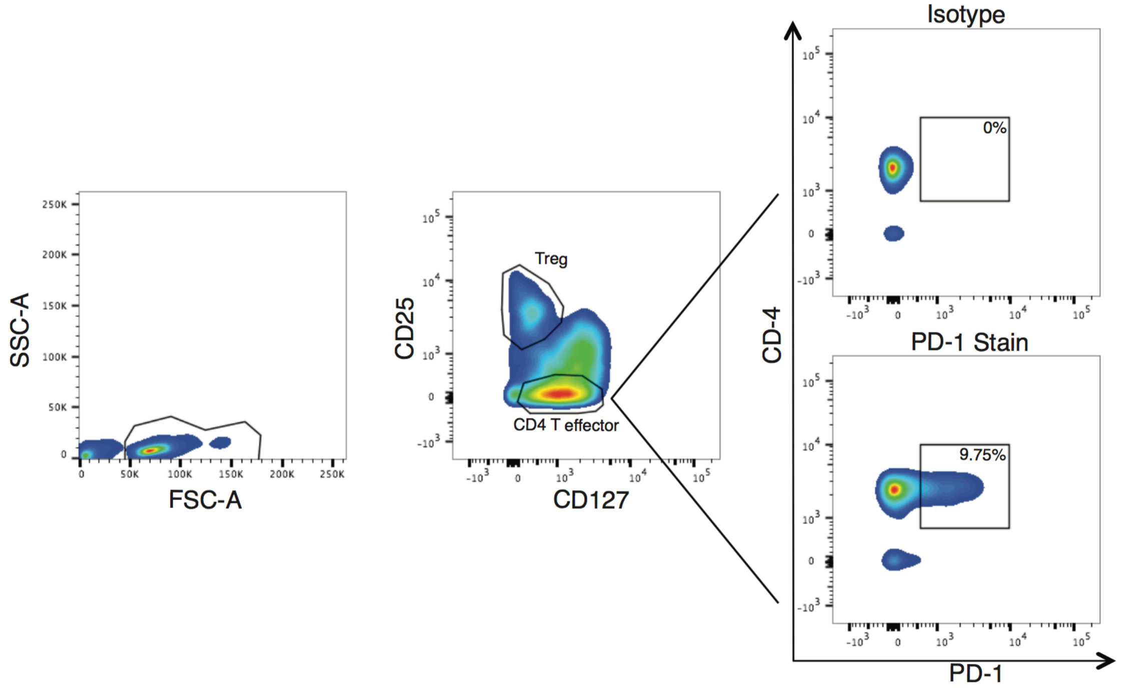

S1 Fig. Gating strategy for the isolation of PD-1⁺ CD4 effector cells. Representative plot showing gates for CD4 effectors $CD4^{+}CD25^{-}CD127^{+}$) and (Tregs ($CD4^{+}CD25^{hi}CD127^{lo}$) among total $CD4^{+}$ cells based on isotype control and on PD-1 stain.
