# Supplementary material for: Functional differences between PD-1+ and PD-1- CD4+ effector T cells in healthy donors and patients with glioblastoma multiforme
Source: PLoS One. 2017 Sep 7;12(9):e0181538. doi: 10.1371/journal.pone.0181538 (PMC5589094; doi:10.1371/journal.pone.0181538)
Supplement: S2 Fig — CFSE labeled CD4 effectors (CD4+CD25—CD127+) stimulated with αCD3/αCD28/αCD2 for 4 days. Proliferation assessed by CFSE dilution measured by flow cytometry. Representative plots displayed from 1 of 5 independent experiments. (PDF) [file pone.0181538.s002.pdf]

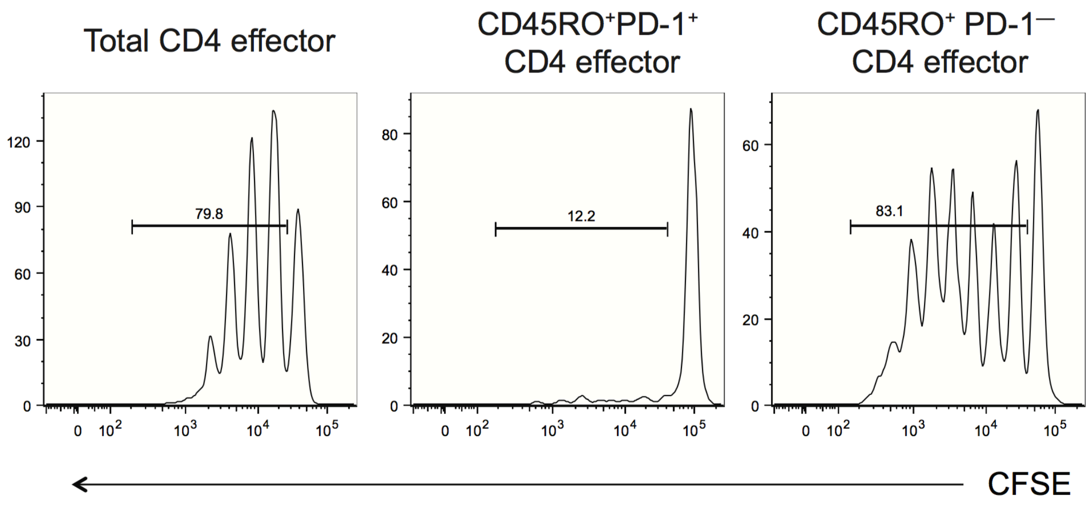

S2 Fig. Proliferative impairment of PD-1+ CD4 effectors independent of CD45RO expression. CFSE labeled CD4 effectors (CD4+CD25— CD127+) stimulated with αCD3/αCD28/αCD2 for 4 days. Proliferation assessed by CFSE dilution measured by flow cytometry. Representative plots displayed from 1 of 5 independent experiments.
