# Supplementary material for: Functional differences between PD-1+ and PD-1- CD4+ effector T cells in healthy donors and patients with glioblastoma multiforme
Source: PLoS One. 2017 Sep 7;12(9):e0181538. doi: 10.1371/journal.pone.0181538 (PMC5589094; doi:10.1371/journal.pone.0181538)
Supplement: S3 Fig — (a) Volcano plot of differentially expressed genes with select genes with padj values > 0.05 highlighted in pink and select genes annotated. (b) Forest plot of p-values of top GO enrichment analysis terms of genes up in PD-1+ CD4 effectors. Data from 3 healthy donors. (PDF) [file pone.0181538.s003.pdf]

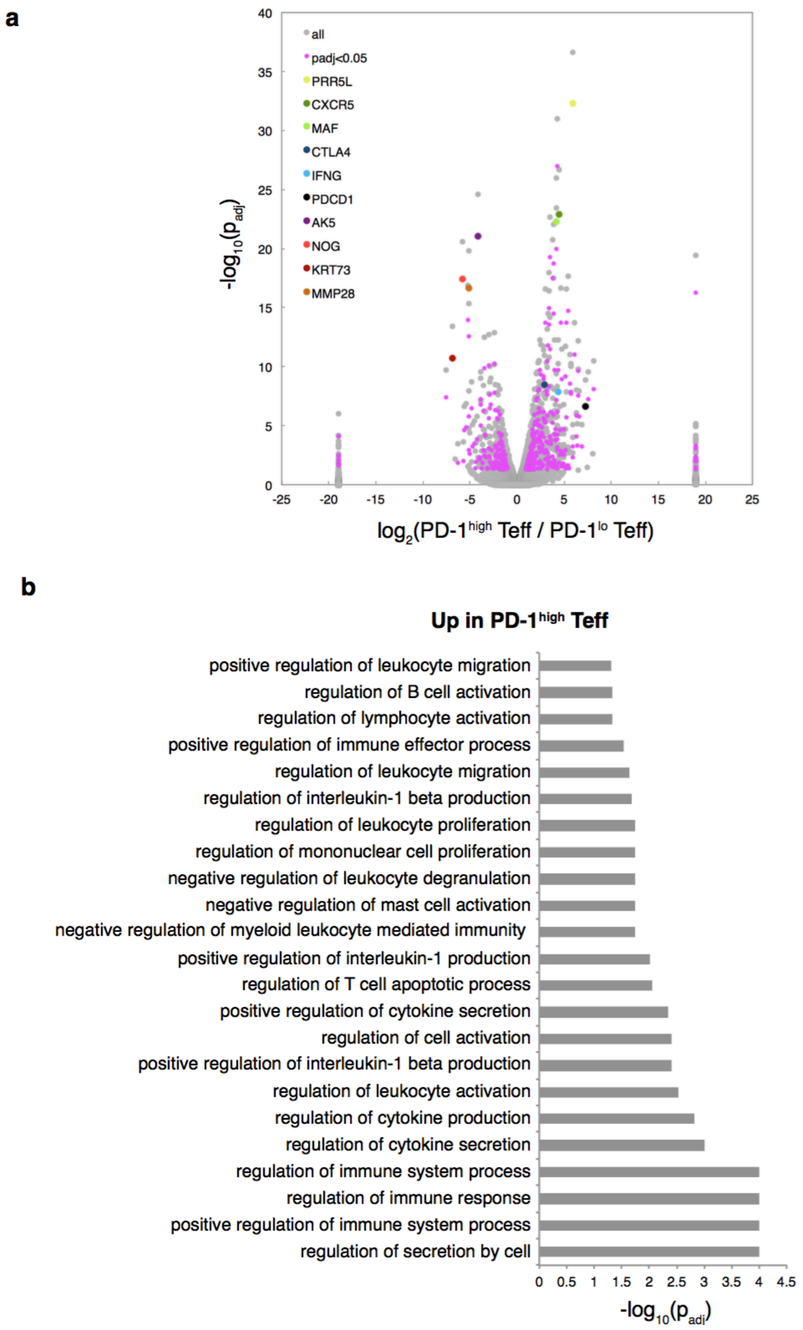

S3 Fig. Differential expression analysis of PD-1⁺ and PD-1⁻ CD4 effectors from healthy donors. (a) Volcano plot of differentially expressed genes with select genes with p_{adj} values > 0.05 highlighted in pink and select genes annotated. (b) Forest plot of p-values of top GO enrichment analysis terms of genes up in PD-1⁺ CD4 effectors. Data from 3 healthy donors.
