# Supplementary material for: Functional differences between PD-1+ and PD-1- CD4+ effector T cells in healthy donors and patients with glioblastoma multiforme
Source: PLoS One. 2017 Sep 7;12(9):e0181538. doi: 10.1371/journal.pone.0181538 (PMC5589094; doi:10.1371/journal.pone.0181538)
Supplement: S4 Fig — Expression of selected cell markers within PD-1+ and PD-1− CD4 effector (CD4+CD25—CD127+) populations (n = 25 total). The percent of cells expressing several markers were significantly different, including CD27 (p = 0.0006), CD45RA (p = 0.0037), CD45RO (p = 0.0032), CD57 (p = 0.0004), CD62L (p = 0.0009), CCR6 (p = 0.0059), CCR7 (p = 0.0021), ICOS (p = 0.0101), PDL1 (p = 0.0061), and LAG3 (p = 0.0008) were all significantly different, with p values indicated by paired students t test. (PDF) [file pone.0181538.s004.pdf]

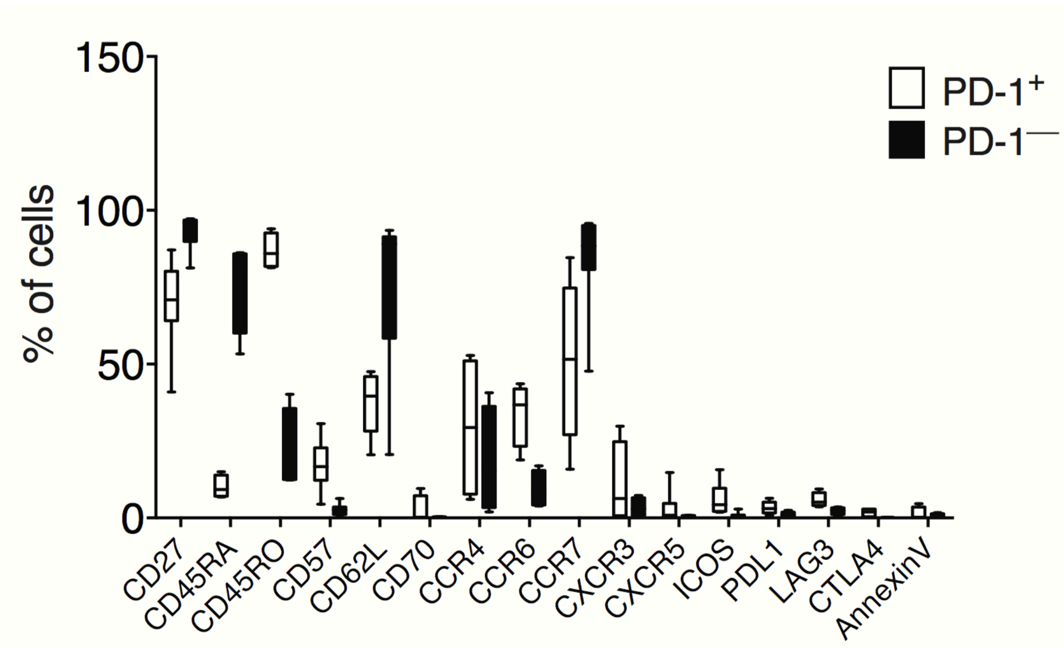

S4 Fig. Characterization of surface markers on PD-1⁺ and PD-1⁻ CD4 effectors. Expression of selected cell markers within PD-1⁺ and PD-1⁻ CD4 effector (CD4⁺CD25⁻CD127⁺) populations (n=25 total). The percent of cells expressing several markers were significantly different, including CD27 (p=0.0006), CD45RA (p=0.0037), CD45RO (p=0.0032), CD57 (p=0.0004), CD62L (p=0.0009), CCR6 (p=0.0059), CCR7 (p=0.0021), ICOS (p=0.0101), PDL1 (p=0.0061), and LAG3 (p=0.0008) were all significantly different, with p values indicated by paired students t test.
