# Supplementary material for: Functional differences between PD-1+ and PD-1- CD4+ effector T cells in healthy donors and patients with glioblastoma multiforme
Source: PLoS One. 2017 Sep 7;12(9):e0181538. doi: 10.1371/journal.pone.0181538 (PMC5589094; doi:10.1371/journal.pone.0181538)
Supplement: S5 Fig — (a) Box plots of z-score normalized luminex measurements for each patient. (b) Correlation matrix of all data from tumors and blood. (PDF) [file pone.0181538.s005.pdf]

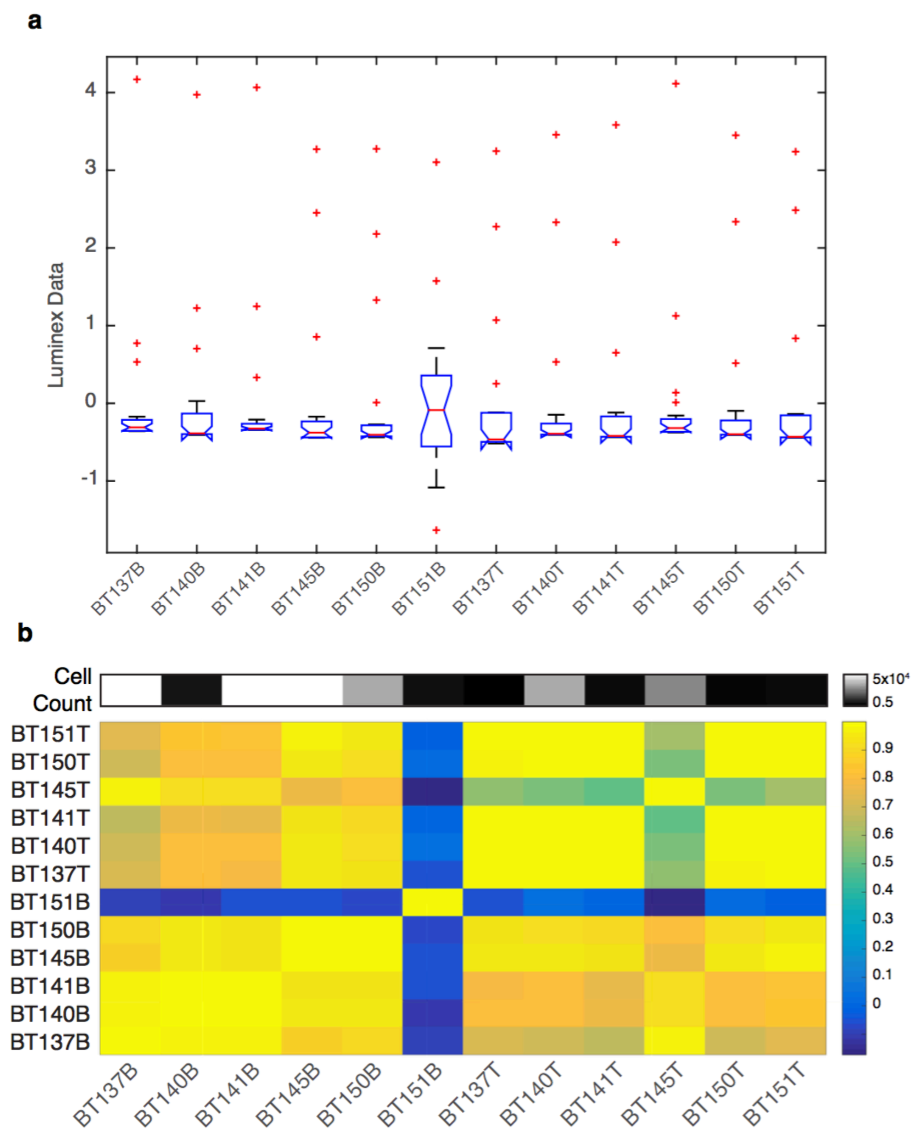

S5 Fig. Analysis of Luminex data from blood and tumor derived CD4 effectors from GBM patients. (a) Box plots of z-score normalized luminex measurements for each patient. (b) Correlation matrix of all data from tumors and blood.
