# Supplementary material for: Functional differences between PD-1+ and PD-1- CD4+ effector T cells in healthy donors and patients with glioblastoma multiforme
Source: PLoS One. 2017 Sep 7;12(9):e0181538. doi: 10.1371/journal.pone.0181538 (PMC5589094; doi:10.1371/journal.pone.0181538)
Supplement: S6 Fig — (a) Principal components analysis of transcriptional data (log2(FPKM+1)>0.01) from all samples analyzed. Data points are labeled from glioblastoma (GBM) blood, tumor, or healthy blood. (b) Percent variance accounted for in each component. (PDF) [file pone.0181538.s006.pdf]

A

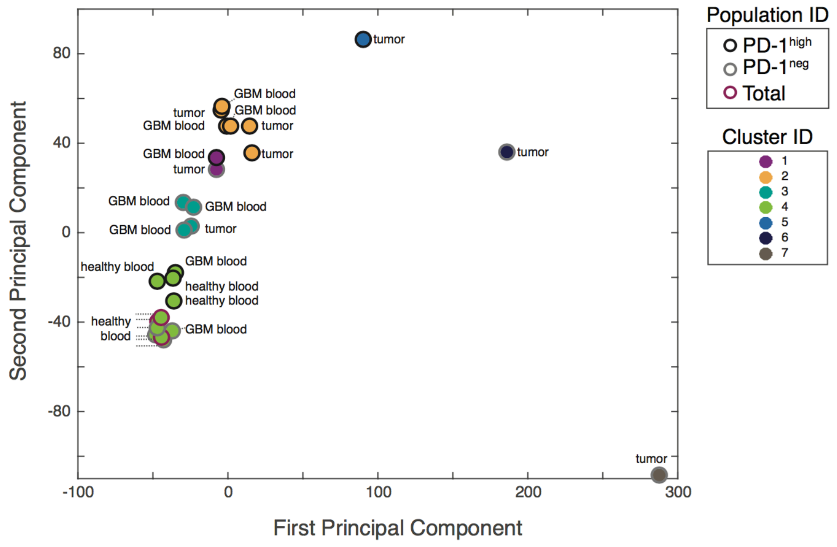

B

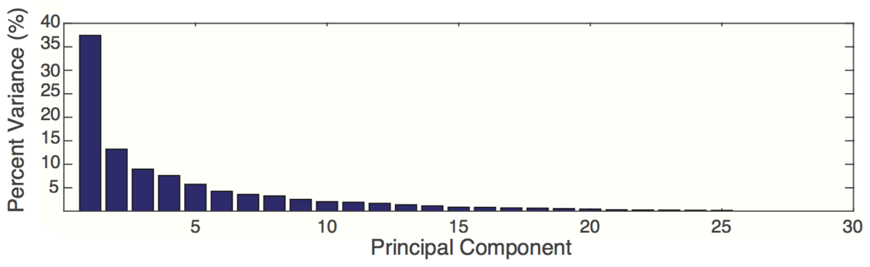

S6 Fig. Principal components analysis (PCA) of transcriptional data from all patients. (a) Principal components analysis of transcriptional data ($\log_2$(FPKM+1)>0.01) from all samples analyzed. Data points are labeled from glioblastoma (GBM) blood, tumor, or healthy blood. (b) Percent variance accounted for in each component.
