# Supplementary figures and images for: Functional differences between PD-1+ and PD-1- CD4+ effector T cells in healthy donors and patients with glioblastoma multiforme
Source: PLoS One. 2017 Sep 7;12(9):e0181538. doi: 10.1371/journal.pone.0181538 (PMC5589094; doi:10.1371/journal.pone.0181538)

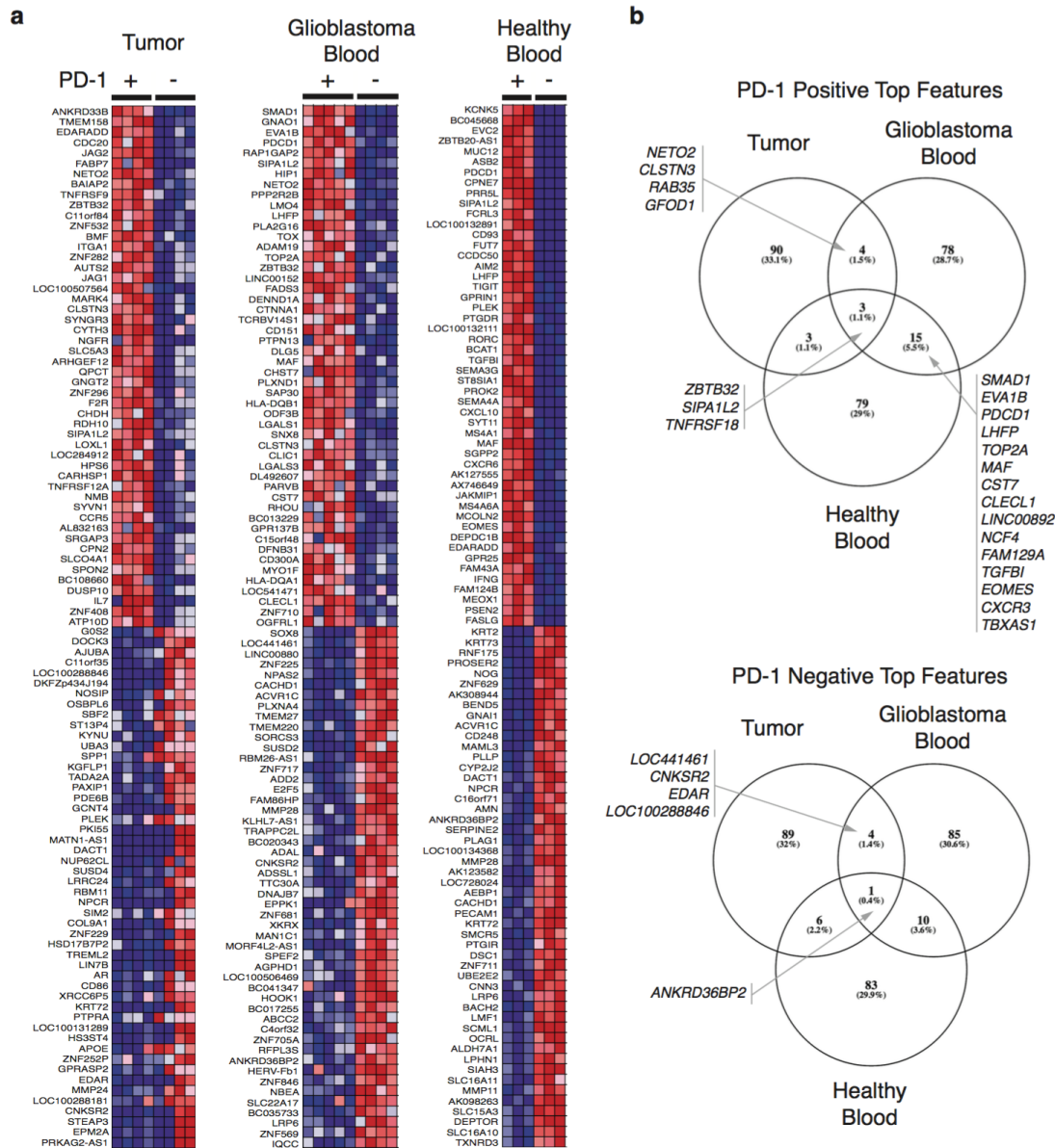

Supplement: S7 Fig — (a) Heatmaps of the top 50 features identified by GSEA are shown for all samples analyzed from PD-1+ and PD-1—CD4 effectors. (b) Venn diagram comparisons of features enriched in PD-1+ (top) or PD-1—(bottom) CD4 effectors. Members of several overlaps are annotated. (PDF) [file pone.0181538.s007.pdf]
