# Supplementary material for: Functional differences between PD-1+ and PD-1- CD4+ effector T cells in healthy donors and patients with glioblastoma multiforme
Source: PLoS One. 2017 Sep 7;12(9):e0181538. doi: 10.1371/journal.pone.0181538 (PMC5589094; doi:10.1371/journal.pone.0181538)
Supplement: S1 Table — (PDF) [file pone.0181538.s008.pdf]

S1 Table. Transcriptional data and sequencing metrics.

Sample	Number of Cells Sorted	Number of reads	Mapped	rRNA%	exon/ intron	exon/ intergenic	5'/3'
134-2_2688-PD1-Teff_N711-N517	30000	1.78E+07	81.82%	0.0048	41.41	268.47	0.62
133-2_2688-PD1plusTeff_N710-N508	30000	1.15E+07	80.64%	0.0041	56.62	380.76	0.58
131-2_2688TotalTeff_N710-N506	30000	1.97E+07	82.24%	0.0047	35.55	218.52	0.62
126_2687-PD1-Teff_N710-N517	30000	2.33E+07	77.89%	0.0097	67.70	343.14	0.77
125_2687-PD1plusTeff_N709-N508	30000	3.05E+07	77.50%	0.0139	98.31	426.52	0.64
123_2687TotalTeff_N709-N506	30000	2.22E+07	79.94%	0.0109	50.39	289.87	0.73
120_2686-PD1-Teffr2_N709-N503	30000	2.16E+07	78.48%	0.0168	70.94	275.21	0.81
119_2686-PD1plusTeffr2_N709-N502	30000	2.38E+07	78.71%	0.0107	76.34	384.01	0.69
117_2686TotalTeffr2_N708-N508	30000	2.84E+07	79.18%	0.0113	48.33	305.37	0.68
108-2_BT229BTeff-PD1-_N707-N507	24806	2.08E+07	82.95%	0.0047	34.32	246.08	0.59
107-2_BT229BTeff-PD1plus_N707-N506	1054	1.47E+07	81.47%	0.0075	37.56	292.50	0.61
100-2_BT228BTeff-PD1-_N706-N507	30000	1.83E+07	80.91%	0.0152	62.84	245.64	0.57
99_BT228BTeff-PD1plus_N706-N506	1650	1.24E+07	79.12%	0.0179	54.21	265.87	0.65
95_BT228TTeff-PD1plus_N706-N502	83	1.28E+07	77.92%	0.0106	43.53	63.41	0.55
92_BT223B-PD1-Teff_N705-N507	857644	2.05E+07	82.29%	0.0050	37.31	148.85	0.72
91_BT223B-PD1plusTeff_N705-N506	221188	1.56E+07	79.60%	0.0056	68.29	296.45	0.67
88_BT223T-PD1plusTeff_N705-N503	296	1.78E+07	76.55%	0.0043	71.43	271.37	0.66
85_BT220B-PD1-Teff_N704-N508		1.92E+07	82.49%	0.0072	36.16	255.85	0.77
84_BT220B-PD1plusTeff_N704-N507		1.58E+07	80.56%	0.0053	43.65	170.15	0.74
81-2_BT220T-PD1-Teff_N704-N504		1.83E+07	76.49%	0.0044	52.42	98.37	0.55
80_BT220T-PD1plusTeff_N704-N503		1.37E+07	82.25%	0.0040	59.24	419.33	0.58
76_BT219B-PD1plusTeff_N703-N507	900	2.85E+07	79.53%	0.0041	44.34	264.61	0.72
72_BT219T-PD1plusTeff_N703-N503	772	1.85E+07	79.73%	0.0043	37.87	77.00	0.69
62_BT215B-PD1negTeff-r1_N702-N517	25000	1.10E+07	79.98%	0.0055	48.10	230.14	0.78
61_BT215B-PD1posTeff-r1_N701-N508	7500	1.95E+07	79.61%	0.0043	51.36	199.56	0.71
65_BT215T-PD1neg-Teff_N702-N504	22594	2.17E+07	80.14%	0.0046	44.28	141.64	0.67
55_BT213B-PD1neg-Teff_N701-N502	5000	1.97E+07	80.69%	0.0046	18.00	22.44	0.75
54_BT213B-PD1pos-Teff_N701-N517	5000	2.41E+07	79.34%	0.0030	24.21	36.48	0.76
58_BT213T-PD1neg-Teff_N701-N505	3125	2.00E+07	80.09%	0.0053	51.55	118.04	0.68
