# Supplementary material for: Functional differences between PD-1+ and PD-1- CD4+ effector T cells in healthy donors and patients with glioblastoma multiforme
Source: PLoS One. 2017 Sep 7;12(9):e0181538. doi: 10.1371/journal.pone.0181538 (PMC5589094; doi:10.1371/journal.pone.0181538)
Supplement: S2 Table — (PDF) [file pone.0181538.s009.pdf]

S2 Table. Selected housekeeping genes used for quality control of transcriptional data.

	Sample Description	PDCD1	CD4	B2M	Enrichment map GSEA	Notes/ Sample Flags
GBM Blood	BT229BTeffPD1plus	4.637	5.559	13.002	yes	
	BT229BTeffPD1neg	1.683	5.888	12.856	yes	
	BT215BPD1posTeff	6.861	6.734	14.105	yes	
	BT215BPD1negTeffr1	0.660	6.898	13.394	yes	
	BT219BPD1plusTeff	5.211	6.151	13.091	yes	
	BT219BPD1negTeff	-	-	-	no	Lower quality
	BT220BPD1plusTeff	6.481	4.639	12.369	yes	
	BT220BPD1negTeff	2.430	4.022	12.139	yes	
	BT228BTeffPD1plus	5.793	5.641	13.108	yes	
	BT228BTeffPD1neg	2.570	5.146	12.615	yes	
GBM Tumor	BT219TPD1plusTeff	7.057	6.587	13.157	yes	
	BT219TPD1negTeff	-	-	-	no	Lower quality
	BT220TPD1plusTeff	8.659	6.490	13.629	yes	
	BT220TPD1negTeff	6.662	1.176	12.908	no	Extreme outlier in PCA
	BT223TPD1plusTeff	7.924	6.507	13.761	yes	
	BT223TPD1negTeff	-	-	-		Lower quality
	BT228TTeffPD1plus	0.941	2.000	12.980	no	Extreme outlier in PCA
	BT228TTeffPD1neg	0.485	1.111	13.286	no	Extreme outlier in PCA
	BT215TPD1posTeff	-	-	-	no	Lower quality
	BT215TPD1negTeff	2.296	6.603	13.374	yes	
	BT213TPD1posTeff	-	-	-	no	Lower quality
	BT213TPD1negTeff	3.658	6.348	13.859	yes	
Healthy Donor	2686TotalTeff	1.891	6.731	13.565	yes	
	2687TotalTeff	1.836	6.747	13.337	yes	
	2688TotalTeff	1.521	6.722	13.189	yes	
	2686PD1plusTeff	5.823	6.811	14.048	yes	
	2686PD1Teffr2	0.632	6.675	13.752	yes	
	2687PD1plusTeff	6.938	7.384	13.910	yes	
	2687PD1negTeff	0.956	6.913	13.452	yes	
	2688PD1plusTeff	5.799	7.096	13.779	yes	
	2688PD1negTeff	1.057	6.830	13.312	yes	
