# Supplementary material for: Functional differences between PD-1+ and PD-1- CD4+ effector T cells in healthy donors and patients with glioblastoma multiforme
Source: PLoS One. 2017 Sep 7;12(9):e0181538. doi: 10.1371/journal.pone.0181538 (PMC5589094; doi:10.1371/journal.pone.0181538)
Supplement: S3 Table — (PDF) [file pone.0181538.s010.pdf]

S3 Table. Differentially expressed genes for PD-1^{+} versus PD-1^{-} CD4 effectors from healthy donors.

hg19.kgXref.geneSymbol	Transcript	HC_Blood_PD1pos_up_vs_HC_Blood_PD1neg_(all_Teff)_log2FoldChange	HC_Blood_PD1pos_up_vs_HC_Blood_PD1neg_(all_Teff)_pval	HC_Blood_PD1pos_up_vs_HC_Blood_PD1neg_(all_Teff)_padj
PRR5L	uc001mwo.4,uc001mwp.3,uc009ykk.3,uc010rfc.2	5.876	2.54E-37	4.92E-33
MYO1F	uc002mkg.3,uc010xkf.2	4.283	1.03E-31	9.92E-28
CXCR5	uc001pue.4,uc001puf.3	4.485	1.92E-27	1.24E-23
MAF	uc002ffm.3,uc002ffn.3	4.210	1.07E-26	5.18E-23
AK5	uc001dhm.2,uc001dhn.3,uc001dho.3	-4.180	2.46E-25	9.52E-22
SYT11	uc001fmg.3,uc010pgq.2	4.153	3.63E-24	1.00E-20
JAKMIP1	uc003giu.4,uc003giv.4,uc010idb.1,uc010idc.1,uc010idd.1,uc010ide.3,uc011bwc.2	5.399	3.38E-24	1.00E-20
ITGB1	uc001iwr.4,uc001iws.4,uc001iwt.4	3.481	2.27E-23	5.50E-20
FAM129A	uc001gra.4,uc001grb.1,uc009wyh.2,uc009wyi.2	3.903	9.28E-23	1.99E-19
TIGIT	uc003ebg.2	6.385	1.57E-21	3.04E-18
CD58	uc001egm.3,uc001egn.3,uc001ego.1,uc001egp.4,uc010owy.2	3.799	1.77E-21	3.11E-18
NOG	uc002iup.2	-5.823	2.46E-21	3.96E-18
MMP28	uc002hjw.1,uc002hjy.1,uc002hjz.1,uc002hka.3	-5.120	1.51E-20	2.25E-17
TOP2A	uc002huq.3,uc002hur.1	19.000	3.75E-20	5.19E-17
TBXAS1	uc003vvh.3,uc003vvi.3,uc003vvj.3,uc010lne.3,uc011kqu.2,uc011kqv.2,uc011kqw.2,uc011kqx.1	4.125	1.11E-19	1.43E-16
STOM	uc004blh.4,uc004bli.4,uc011lyk.3,uc031tew.1,uc031tex.1	3.114	2.46E-19	2.97E-16
PRDM1	uc003prd.2,uc003pre.3	3.307	5.07E-19	5.77E-16
FAS	uc001kfr.3,uc001kfs.3,uc001kft.3,uc001kfw.3,uc009xtp.3,uc010qna.2,uc010qnb.2,uc010qnc.2,uc010qnd.2,uc010qne.2,uc031pwn.1	3.382	1.03E-18	1.10E-15
CX3CR1	uc003cjl.3,uc021wwa.1,uc021wwb.1,uc021wwc.1,uc021wwd.1	4.779	1.75E-18	1.78E-15
RORC	uc001ezg.3,uc001ezh.3,uc010pdo.2,uc010pdp.2	5.444	2.10E-18	2.03E-15
MCOLN2	uc001dkm.3,uc001dkn.3	3.927	3.42E-18	3.15E-15
KRT72	uc001saq.2,uc001sar.2,uc010sns.1,uc010snt.1	-5.261	1.21E-17	1.06E-14
MIAT	uc003ada.2,uc003adb.2,uc003adc.2,uc003add.2,uc003adf.2	3.019	1.81E-17	1.52E-14
PCSK5	uc004ajy.2,uc004ajz.3,uc004aka.3,uc004akb.3,uc004akc.2	-4.671	1.98E-17	1.60E-14
IGFBP4	uc002hus.3	4.614	2.23E-17	1.73E-14
AHNAK	uc001ntk.2,uc001ntl.3	3.048	2.45E-17	1.82E-14
CHN1	uc002ujg.3,uc002uji.3,uc002ujj.3,uc010zeq.2	5.209	2.77E-17	1.98E-14
CDCA7	uc002uic.1,uc002uid.1,uc010zej.1,uc010zek.1	3.444	3.99E-17	2.75E-14
IRF5	uc003vog.3,uc003voh.3,uc003voi.3,uc003voj.4,uc003vok.2,uc010llr.1,uc010lls.1,uc010llt.3,uc010llu.1,uc010llv.1,uc010llw.1,uc011kot.1,uc011kou.1	4.247	5.37E-17	3.58E-14
CCDC50	uc003fsv.3,uc003fsw.3	3.713	8.78E-17	5.66E-14
GPR125	uc003gql.2,uc003gqm.2,uc003gqn.1,uc003gqo.4,uc010ieo.2	-4.097	1.21E-16	7.58E-14
GZMA	uc003jpm.3	4.132	3.82E-16	2.31E-13
DACT1	uc001xdw.3,uc001xdx.3,uc010trv.2,uc010trw.2	-5.099	4.55E-16	2.67E-13

CCR2	uc003cpm.4,uc003cpn.4,uc021wxa.1	4.232	1.37E-15	7.77E-13
ADTRP	uc003naa.3,uc003nab.3,uc011dip.2,uc031smt.1	-2.978	2.01E-15	1.11E-12
TNFRSF4	uc001ade.3,uc001adf.3	3.292	3.16E-15	1.70E-12
NOD2	uc002egl.1,uc002egm.1,uc010cbj.1,uc010cbk.1,uc010cbl.1,uc010cbm.1,uc010cbn.1,uc010cbo.1,uc010cbp.1,uc010cbq.1,uc010cbr.1,uc010vgq.1,uc021tia.1	3.470	6.36E-15	3.33E-12
SCML1	uc004cyb.3,uc004cyc.3,uc004cyd.3,uc004cye.3	-3.478	1.82E-14	9.26E-12
PLEK	uc002sen.4,uc010fde.3	6.106	2.00E-14	9.91E-12
CCR6	uc003qvl.3,uc003qvm.4,uc003qvn.4,uc010kkm.3	2.901	2.81E-14	1.36E-11
CTNNA1	uc003ldh.3,uc003ldi.3,uc003ldj.3,uc003ldl.3,uc011cyx.2,uc011cyy.2	3.565	2.98E-14	1.41E-11
KRT73	uc001sas.3	-6.880	3.86E-14	1.78E-11
ST8SIA1	uc001rfo.4,uc009zix.3	3.243	6.63E-14	2.98E-11
CCR4	uc003cfg.1,uc021wuw.1	3.395	8.76E-14	3.85E-11
TRABD2A	uc002sou.5,uc002sov.3,uc010fgc.3,uc010ysl.3	-2.409	1.25E-13	5.36E-11
EVC2	uc003gij.3,uc003gik.3,uc011bwb.2	4.783	1.52E-13	6.38E-11
NET1	uc001iia.4,uc001iib.4,uc010qar.3,uc010qas.3	-2.985	2.00E-13	8.22E-11
SATB1	uc003cbh.3,uc003cbi.3,uc003cbj.3	-2.597	2.79E-13	1.12E-10
EPHA1-AS1	uc003wda.4	-2.989	2.88E-13	1.14E-10
DSC1	uc002kwm.3,uc002kwn.3	-3.450	3.24E-13	1.25E-10
BACH2	uc003pnw.3,uc010kch.3,uc011eab.2	-2.831	3.42E-13	1.30E-10
PAM	uc003kns.3,uc003knt.3,uc003knu.3,uc003knv.3,uc003knw.3,uc003knx.1,uc003kny.1,uc003knz.3,uc011cuz.2	2.711	4.13E-13	1.54E-10
HNRPLL	uc002rqv.3,uc021vgb.1,uc021vgc.1	2.455	5.32E-13	1.91E-10
LINC00152	uc002ssk.4,uc010fgy.3,uc010fgz.3	4.392	5.26E-13	1.91E-10
GPR25	uc001gvn.2	4.020	5.69E-13	2.00E-10
NKG7	uc002pwj.3,uc002pwk.3	6.503	6.62E-13	2.29E-10
PYHIN1	uc001fta.4,uc001ftb.3,uc001ftc.3,uc001ftd.3,uc001fte.3	2.559	1.34E-12	4.56E-10
FCRL6	uc001fuc.2,uc001fud.4,uc009wsz.1,uc009wta.3,uc010pix.1	5.178	1.85E-12	6.15E-10
CYB561	uc002jap.3,uc002jaq.3,uc002jar.3,uc002jas.3,uc002jat.3,uc010ddt.3,uc010wpf.2,uc010wpg.2	2.782	1.88E-12	6.15E-10
CXCR6	uc003cpc.1,uc010hix.1,uc021www.1	5.414	2.75E-12	8.85E-10
CPNE7	uc002fnp.3,uc002fnq.3	4.709	3.46E-12	1.10E-09
WEE1	uc001mhs.3,uc001mht.3,uc001mhu.3	2.800	3.79E-12	1.18E-09
KCNK5	uc003oon.3	19.000	5.91E-12	1.82E-09
FGFR1	uc003xlp.3,uc003xlu.3,uc003xlv.3,uc003xlw.1,uc010lwf.3,uc010lwk.3,uc011lbr.2,uc011lbs.2,uc011lbt.1,uc011lbu.2,uc011lbv.2,uc011lbw.2,uc011lbx.1,uc022aua.1,uc022aub.1,uc022auc.1,uc022aud.1	2.984	6.78E-12	2.05E-09
AEBP1	uc003tkb.4,uc003tkc.5,uc003tkd.4,uc022aci.2	-3.876	8.25E-12	2.45E-09
EDARADD	uc001hxu.1,uc001hxv.1	5.637	9.03E-12	2.61E-09
RNF175	uc003int.3,uc003inu.1	-5.670	9.03E-12	2.61E-09
CTLA4	uc002vak.2,uc002val.2,uc010fty.2,uc010ftz.2	2.899	1.12E-11	3.18E-09
NUSAP1	uc001znr.4,uc001zns.4,uc001znt.4,uc001znu.4,uc001znv.4,uc001znw.4,uc010ucw.2	3.379	1.57E-11	4.40E-09
FAM153A	uc003mib.1,uc003mic.3,uc010jkp.1,uc021yix.1	-2.618	1.62E-11	4.46E-09
YWHAH	uc003alz.3,uc003ama.3,uc010gwm.3	2.406	2.18E-11	5.93E-09
SLC16A10	uc003pur.4,uc003pus.3,uc003put.3	-3.993	2.32E-11	6.23E-09

ASB2	uc001ycb.2,uc001ycc.2,uc001ycd.3,uc001yce.1	8.092	3.22E-11	8.53E-09
CD84	uc001fwf.4,uc001fwg.4,uc001fwh.4,uc001fwi.4,uc001fwj.3,uc001fwk.3,uc009wtn.3	2.311	3.31E-11	8.66E-09
LIMS1	uc002teg.3,uc002teh.3,uc002tei.3,uc002tej.3,uc002tek.4,uc002tel.3	2.301	3.48E-11	8.85E-09
UBE2E2	uc003ccg.2,uc010hfc.2	-4.122	3.44E-11	8.85E-09
NCF4	uc003apy.4,uc003apz.4	4.394	3.54E-11	8.89E-09
LINC00426	uc001usu.3	3.096	4.33E-11	1.07E-08
ICA1	uc003srm.3,uc003srn.5,uc003sro.5,uc003srq.4,uc003srr.4,uc003srs.2,uc010ktr.3,uc010kts.4	3.305	4.45E-11	1.09E-08
TBK1	uc001ssc.2	2.284	4.63E-11	1.12E-08
HEMGN	uc004axy.4,uc004axz.4	-2.967	5.22E-11	1.25E-08
IFNG	uc001stw.1	4.336	5.57E-11	1.31E-08
SIPA1L2	uc001hvf.3,uc001hvg.3	5.695	5.98E-11	1.39E-08
LINC00892	uc022cew.1,uc022cex.1	4.395	6.70E-11	1.54E-08
APBA2	uc001zck.3,uc001zcl.3,uc001zcm.1,uc010azj.2,uc010uas.1,uc010uat.2	-2.398	7.08E-11	1.61E-08
ZEB2	uc002tvu.3,uc002tvv.3,uc002tvw.3,uc002tvx.1,uc002tvy.3,uc002twa.3,uc010fnp.3,uc010fnq.1,uc010zbm.2,uc021vqh.1,uc031rpm.1	5.705	8.93E-11	2.01E-08
TMEM220	uc002gmx.3,uc002gmy.3	-2.916	1.01E-10	2.24E-08
ADAM19	uc003lww.3,uc003lwy.3,uc003lwz.4,uc011ddr.1,uc021ygq.1	3.902	1.07E-10	2.34E-08
CSF1	uc001dyt.2,uc001dyu.2,uc001dyw.4,uc021orj.2	3.170	1.16E-10	2.49E-08
MAP3K5	uc003qhc.3,uc010kgw.1,uc011edj.2,uc011edk.1	2.284	1.16E-10	2.49E-08
LOC100132891	uc003xyy.3,uc011lff.2,uc022avt.1	19.000	1.20E-10	2.55E-08
NCALD	uc003yke.3,uc003ykf.3,uc003ykg.3,uc003ykh.3,uc003yki.3,uc003ykj.3,uc003ykk.3,uc003ykl.3	2.468	1.23E-10	2.58E-08
SMARCA1	uc004eun.4,uc004eup.4,uc011muk.1,uc011mul.1	-4.110	1.26E-10	2.62E-08
PHACTR2	uc003qjq.4,uc003qjr.4,uc010khh.3,uc010khi.3	2.185	1.27E-10	2.62E-08
TNFRSF18	uc001ada.3,uc001adb.3,uc001adc.3,uc001add.3	6.523	1.48E-10	3.01E-08
CD74	uc003lsc.3,uc003lsd.3,uc003lse.3	2.129	1.79E-10	3.61E-08
CR2	uc001hfv.3,uc001hfw.3,uc009xch.3,uc009xci.1	-7.577	1.86E-10	3.71E-08
CXCR3	uc004eaf.3,uc011mpx.2,uc022bys.1	4.903	1.92E-10	3.78E-08
GPR160	uc003fgi.3,uc010hwq.3,uc021xhe.1	-2.729	2.46E-10	4.81E-08
CD82	uc001myc.3,uc001myd.3	2.156	2.61E-10	5.04E-08
LMF1	uc002ckm.1	-3.835	2.81E-10	5.37E-08
FCRL3	uc001fqx.4,uc001fqy.4,uc001fqz.4,uc001fra.3,uc001frb.3,uc001frc.1,uc009wsn.3,uc009wso.3	7.588	2.90E-10	5.50E-08
IL10RA	uc001prv.3,uc001prw.3,uc010rxl.2,uc010rxm.2,uc010rxn.2,uc031qel.1	2.013	3.60E-10	6.76E-08
FGFR2	uc001lfg.4,uc001lfk.1,uc001lfn.5,uc001lfo.1,uc010qtl.2,uc010qtm.2,uc010qtn.2,uc010qto.2,uc010qtp.2,uc010qtq.2,uc021pzv.1,uc021pzw.1,uc021pzx.1,uc021pzy.1,uc021pzz.1,uc021qaa.1,uc021qab.1,uc021qac.1,uc031pxk.1	4.205	3.66E-10	6.81E-08
GCNT1	uc004akf.4,uc004akh.4,uc010mpf.3,uc010mpg.3,uc010mph.3,uc010mpi.3,uc022bif.1	3.052	6.32E-10	1.16E-07
PLAG1	uc003xsq.4,uc003xsr.4,uc010lyi.3,uc010lyj.3,uc022aur.1	-2.945	6.68E-10	1.22E-07
HOPX	uc003hbz.2,uc003hca.2,uc003hcb.2,uc003hcc.2,uc003hcd.2,uc003hce.2,uc011cad.1,uc031sei.1	4.058	6.78E-10	1.22E-07
PPP2R2B	uc003loe.4,uc003lof.5,uc003log.5,uc003loh.5,uc003loi.5,uc003loj.5,uc003lok.5,uc010jgm.4,uc011dbu.3,uc011dbv.3	4.840	8.12E-10	1.45E-07
ZNF667-AS1	uc002qnf.3,uc002qng.3,uc021vcf.1	-3.879	9.10E-10	1.61E-07
GNAI1	uc003uhb.1,uc011kgt.1	-4.518	9.29E-10	1.63E-07

PECAM1	uc002jef.2	-2.839	9.83E-10	1.70E-07
TYMP	uc003bmb.5,uc003bmc.5,uc003bmd.5,uc003bme.5,uc010hbd.4,uc011arz.1	3.578	9.87E-10	1.70E-07
CHST7	uc004dgt.3,uc022bvm.1	2.958	1.10E-09	1.88E-07
UST	uc003qmg.3	3.062	1.29E-09	2.18E-07
PDCD1	uc002wcq.4,uc010fzs.3,uc010fzt.3	7.247	1.32E-09	2.22E-07
FUT7	uc004ckq.2	4.634	1.53E-09	2.55E-07
CACHD1	uc001dbo.1,uc001dbp.1,uc001dbq.1,uc010opa.1,uc021ooi.1	-4.842	1.88E-09	3.08E-07
ARHGAP35	uc010ekv.3	2.294	1.88E-09	3.08E-07
IGF1R	uc002bul.3,uc010bon.3,uc010boo.1,uc010urq.2,uc010urr.1,uc021sxi.1	-2.862	2.07E-09	3.36E-07
CLIC5	uc003oxu.4,uc003oxv.3,uc003oxw.3,uc003oxx.4,uc031sos.1,uc031sot.1	3.081	2.28E-09	3.68E-07
ATXN1	uc003nbt.3,uc003nbu.1,uc003nbv.1,uc003nbw.1,uc010jpi.3,uc010jpj.1	2.349	2.64E-09	4.23E-07
PLXDC1	uc002hrg.2,uc002hrh.2,uc002hri.2,uc002hrj.1,uc002hrk.1,uc010cvr.1	-2.273	3.09E-09	4.89E-07
IGFBP3	uc003tnq.3,uc003tnr.3,uc003tns.3,uc003tnt.3	3.233	3.67E-09	5.77E-07
RIN1	uc001ohn.1,uc009yrd.1,uc010roy.1,uc010roz.1,uc010rpa.1	-3.565	3.84E-09	5.97E-07
MYO15B	uc002jor.1,uc002jos.4,uc010dgi.1	-2.013	3.86E-09	5.97E-07
AIM2	uc001ftj.1	4.256	4.41E-09	6.72E-07
CTSH	uc010unf.1,uc010ung.1,uc021srk.1	2.459	4.42E-09	6.72E-07
OASL	uc001tzj.2,uc001tzk.2,uc031qjs.1	2.831	4.52E-09	6.83E-07
ANXA2	uc002agk.3,uc002agl.3,uc002agm.3,uc002agn.3,uc010bgj.3,uc010uhd.2,uc021snc.1	3.578	4.77E-09	7.15E-07
ANXA5	uc003idu.4,uc003idv.4	2.017	4.93E-09	7.34E-07
TNFRSF1B	uc001att.3,uc001atu.3,uc009vnk.3	2.248	5.89E-09	8.69E-07
BAIAP3	uc002clj.3,uc002clk.2,uc010uuz.2,uc010uva.2,uc010uvb.2,uc010uvc.1,uc021tag.1	3.852	7.38E-09	1.08E-06
KLF6	uc001iha.3,uc001ihb.2,uc010qaj.2,uc010qak.2,uc010qal.2	1.822	8.13E-09	1.18E-06
DPP4	uc002ubz.3,uc002uca.1,uc002ucb.1,uc010fpb.3	-1.860	8.63E-09	1.24E-06
RYR1	uc002oit.3,uc002oiu.3,uc002oiv.2,uc010xuf.1	6.315	9.05E-09	1.30E-06
NETO2	uc002eeq.2,uc002eer.2,uc010vgf.2	3.159	9.16E-09	1.30E-06
ZC2HC1A	uc003ybd.3	2.518	9.69E-09	1.37E-06
ADD2	uc002sgy.3,uc002sgz.3,uc002shc.2,uc010fds.2,uc010fdt.2,uc010fdu.2,uc021vjc.1	-5.113	1.10E-08	1.54E-06
MYO7A	uc001oyb.2,uc001oyc.2,uc001oyd.3,uc001oye.2,uc009yus.1,uc009yut.1,uc010rsl.2,uc010rsm.1	6.601	1.20E-08	1.67E-06
SEMA4A	uc001fnl.3,uc001fnm.3,uc001fnn.3,uc001fno.3,uc009wrq.3	5.282	1.48E-08	2.04E-06
MAN1C1	uc001bkm.2,uc009vry.1	-2.097	1.59E-08	2.19E-06
APOBEC3F	uc003awv.3,uc003aww.3,uc003awx.3,uc003awy.3,uc011aog.1,uc021wpr.1	1.845	1.69E-08	2.30E-06
MICAL2	uc001mjy.3,uc001mjz.3,uc001mka.3,uc001mkb.3,uc001mkc.3,uc001mkd.3,uc001mke.3,uc001mkf.3,uc010rch.1,uc010rci.2,uc010rcj.2	2.838	1.75E-08	2.35E-06
EPHA1	uc003wcz.3	-2.329	1.74E-08	2.35E-06
FAM213A	uc001kcc.4,uc001kcd.4,uc001kce.4,uc001kcf.4,uc021pux.1,uc021puy.1	-3.009	1.78E-08	2.37E-06
CLDND1	uc003dso.2,uc003dsp.3,uc003dsq.3,uc003dsr.3,uc003dss.3,uc003dst.3,uc003dsu.3,uc003dsv.3	1.897	2.03E-08	2.69E-06
ARHGAP18	uc003qbr.3,uc011ebw.2,uc021zfe.1	3.039	2.18E-08	2.86E-06
PDK1	uc002uhq.1,uc002uhs.3,uc010zdz.1,uc010zea.1,uc010zeb.2	-1.977	2.20E-08	2.86E-06
TOX	uc003xtw.1	3.207	2.21E-08	2.86E-06
TGFBI	uc003lbf.4,uc003lbg.4,uc003lbh.4,uc010jee.3,uc011cyb.2	6.817	2.38E-08	3.07E-06

CD300A	uc002jkv.4,uc002jkw.4,uc010dfr.4,uc010dfs.4,uc031rej.1	2.231	2.55E-08	3.27E-06
SPSB1	uc001apv.3,uc010oae.2	2.947	2.84E-08	3.61E-06
LGALS3	uc001xbr.3,uc021rtj.1	2.953	3.11E-08	3.93E-06
LRRN3	uc003vfs.4,uc003vft.4,uc003vfu.4,uc022akc.1	-3.528	3.33E-08	4.18E-06
FAM153B	uc010jjy.1,uc031sma.1,uc031smb.1,uc031smc.1,uc031smd.1	-2.475	3.54E-08	4.42E-06
DTHD1	uc003gsv.4,uc011bxy.3,uc021xne.2	6.611	3.80E-08	4.71E-06
RNF214	uc001pqt.4,uc001pqu.3,uc010rxf.2	1.913	4.06E-08	5.00E-06
ITPRIPL1	uc002svx.3,uc002svy.3,uc010yuk.2,uc010yul.2	2.964	4.80E-08	5.87E-06
GBP5	uc001dnc.3,uc001dnd.3,uc001dne.1	1.941	5.07E-08	6.12E-06
PAQR4	uc002csj.4,uc002csk.4,uc002csl.4,uc010uwm.2	3.797	5.09E-08	6.12E-06
FRMD4B	uc003dnu.2,uc003dnv.2,uc003dnw.2,uc003dnx.1,uc003dny.3,uc011bga.1	2.847	5.08E-08	6.12E-06
ANK1	uc003xoc.3,uc003xod.3,uc003xof.3,uc003xoh.3,uc003xoi.3,uc003xoj.3,uc003xok.3,uc003xol.3,uc003xom.3,uc011lcl.2	2.543	5.47E-08	6.53E-06
NABP1	uc002usw.3,uc002usx.3,uc002usy.3,uc021vuf.1,uc021vug.1	2.084	5.50E-08	6.53E-06
SLAMF6	uc001fwd.2,uc001fwe.2,uc009wtm.2,uc010pjh.2,uc010pji.2,uc010pjj.2	1.836	6.71E-08	7.92E-06
GTDC1	uc002tvo.3,uc002tvp.3,uc002tvr.3,uc002tvs.3,uc002tvt.2,uc010fnn.3,uc010fno.3,uc021vqf.1,uc021vqg.1	2.231	7.34E-08	8.61E-06
SERPINE2	uc002vnu.2,uc002vnv.2,uc010zlr.1,uc031rrk.1	-3.870	7.67E-08	8.93E-06
ANXA4	uc002sfr.4,uc002sfs.4,uc010yqn.1,uc010yqo.2	2.372	7.78E-08	9.01E-06
IL2RB	uc003aqv.1	3.425	8.31E-08	9.56E-06
MB21D1	uc003pgx.1	2.543	8.53E-08	9.76E-06
FBXL8	uc002erk.1	2.296	8.67E-08	9.86E-06
NLGN4Y	uc004fte.2,uc004ftf.2,uc004ftg.2,uc004fth.2,uc004fti.4,uc011nas.1	3.714	1.02E-07	1.16E-05
SLC22A23	uc003mvm.3,uc003mvn.3,uc003mvo.3,uc003mvp.1,uc003mvq.2,uc010jnn.3,uc010jno.2	-2.237	1.15E-07	1.30E-05
PTK2	uc003yvo.3,uc003yvp.3,uc003yvq.3,uc003yvr.3,uc003yvs.3,uc003yvt.3,uc003yvu.3,uc003yvv.3,uc011ljp.2,uc011ljq.2,uc011ljr.2	-2.464	1.27E-07	1.42E-05
GAL3ST4	uc003utt.3,uc003utu.3,uc022aii.1	-2.443	1.28E-07	1.43E-05
PLLP	uc002elg.2	-5.412	1.30E-07	1.43E-05
SLC1A5	uc002pfq.3,uc002pfr.3,uc002pfs.3,uc010xyh.2	2.641	1.31E-07	1.44E-05
SMAD1	uc003ikc.3,uc003ikd.3,uc003ikf.3,uc010iov.3,uc011cic.2	3.051	1.58E-07	1.72E-05
LAIR2	uc002qga.1,uc002qgb.1,uc002qgc.3,uc002qgd.3,uc010erl.3	4.071	1.71E-07	1.85E-05
SGPP2	uc010zlo.2,uc010zlp.2	4.980	1.75E-07	1.89E-05
RAB37	uc002jlc.2,uc002jld.2,uc002jlk.3,uc010dfu.3,uc010wrb.2,uc010wrc.2,uc010wre.2	1.682	1.92E-07	2.07E-05
USP46	uc003gzm.4,uc003gzn.3,uc011bzr.2,uc011bzs.2	2.644	1.96E-07	2.10E-05
PTGDR	uc001wzq.3	4.516	2.24E-07	2.38E-05
BC045668	uc003iet.3	19.000	2.28E-07	2.41E-05
BEND5	uc001crw.4,uc001crx.4	-5.571	2.38E-07	2.49E-05
CD99	uc004fpz.3,uc004fqa.4,uc010nwa.3	1.916	2.38E-07	2.49E-05
CDCA7L	uc003sve.4,uc003svf.4,uc010kuk.3,uc010kul.3	-1.933	2.82E-07	2.93E-05
CPM	uc001sup.3,uc001suq.3,uc001sur.3	2.102	2.98E-07	3.08E-05
CALHM2	uc001kxa.3,uc001kxb.3,uc001kxc.3,uc001kxd.1	2.627	3.64E-07	3.73E-05
NELL2	uc001rof.3,uc001rog.2,uc001roh.2,uc001roi.1,uc001roj.2,uc009zkd.2,uc010skz.1,uc010sla.1,uc010slb.1	-1.889	3.63E-07	3.73E-05
TRADD	uc002erh.1,uc002eri.1,uc010vjb.1	1.667	4.39E-07	4.47E-05

OPTN	uc001ilu.1,uc001ilv.1,uc001ilw.1,uc001ilx.1,uc001ily.1,uc010qbr.1	1.706	4.55E-07	4.58E-05
ELOVL5	uc003pbq.1,uc003pbr.2,uc003pbs.2,uc003pbt.4,uc003pbu.3,uc011dwx.2,uc011dwy.1	1.661	4.55E-07	4.58E-05
WSB2	uc001twr.2,uc009zws.1,uc010sza.1,uc010szb.1	1.832	4.60E-07	4.61E-05
MYO1G	uc003tmg.2,uc003tmh.2,uc003tmi.1,uc003tmj.2,uc010kym.2,uc022acj.1	1.548	4.82E-07	4.78E-05
CDK14	uc003ukt.1,uc003uku.1,uc003ukv.1,uc003ukx.1,uc003uky.2,uc003ukz.1,uc010les.1,uc011khl.1	19.000	4.81E-07	4.78E-05
COTL1	uc002fid.3	1.616	4.87E-07	4.81E-05
TPST2	uc003acw.3,uc003acx.3,uc011akf.1	1.650	4.91E-07	4.82E-05
NMNAT3	uc003etj.3,uc003etk.3,uc003etl.3,uc010hul.3	-3.970	5.21E-07	5.09E-05
SRXN1	uc002wea.4	3.946	5.28E-07	5.13E-05
ACTN1	uc001xkk.3,uc001xkl.3,uc001xkm.3,uc001xkn.3,uc001xko.1,uc010ttb.2,uc010ttc.2,uc010ttd.1	-1.613	5.46E-07	5.28E-05
BCAT1	uc001rgc.3,uc001rgd.4,uc001rge.4,uc010six.2,uc010siy.2	4.254	5.68E-07	5.47E-05
MEOX1	uc002idz.3,uc002iea.3,uc002ieb.3	4.043	5.75E-07	5.51E-05
ACVR1C	uc002tzk.4,uc002tzl.4,uc010foe.3,uc010fof.3	-3.358	5.94E-07	5.66E-05
SSBP4	uc002niy.3,uc002niz.3,uc010ebp.3	1.680	6.80E-07	6.44E-05
ZNF629	uc002dzs.1	-3.353	6.94E-07	6.55E-05
FAR2	uc001ris.5,uc001rit.4,uc009zjm.4	2.421	7.54E-07	7.08E-05
TSPAN5	uc003hub.3,uc011cdz.2	1.812	7.88E-07	7.36E-05
GALM	uc002rqy.3	1.639	7.97E-07	7.41E-05
CD93	uc002wsv.3	6.261	8.14E-07	7.53E-05
SYNE2	uc001xgk.3,uc001xgl.3,uc001xgm.3,uc001xgn.3,uc001xgo.3,uc001xgp.3,uc001xgq.3,uc001xgr.3,uc001xgs.3,uc001xgt.3,uc010apw.1,uc010apx.1,uc010apy.3,uc010apz.1,uc010aqa.3,uc010tsi.2,uc021ruh.1,uc021rui.1	1.708	8.25E-07	7.60E-05
HKDC1	uc001jpf.4,uc009xqb.3,uc010qje.2	-1.719	8.36E-07	7.63E-05
SNX9	uc003qqv.1	-1.850	8.35E-07	7.63E-05
B3GNT9	uc002erf.3,uc021tka.1	2.800	9.56E-07	8.68E-05
STX11	uc003qks.4,uc021zgk.1	2.687	9.68E-07	8.75E-05
CD248	uc001ohm.1	-19.000	9.86E-07	8.83E-05
LACC1	uc001uzf.4,uc010acg.3	3.468	9.86E-07	8.83E-05
ACOT7	uc001amq.3,uc001amr.3,uc001ams.3,uc001amt.3,uc001amu.3	2.409	1.09E-06	9.70E-05
PERP	uc003qht.2	2.766	1.16E-06	1.03E-04
C1orf216	uc001bzh.1,uc021oli.1	1.777	1.25E-06	1.10E-04
NEK6	uc004bof.3,uc004bog.3,uc004boh.3,uc004boi.3,uc010mwk.3,uc022bnf.1,uc022bng.1	3.173	1.32E-06	1.16E-04
ZNF711	uc004eeo.3,uc004eep.3,uc004eeq.3,uc011mqy.1	-3.237	1.41E-06	1.24E-04
AK127555	uc003nar.3	4.002	1.45E-06	1.26E-04
LOC541471	uc002the.2,uc002thf.4,uc010yxl.1	4.224	1.63E-06	1.41E-04
PRF1	uc001jrf.4,uc009xqg.3	2.117	1.69E-06	1.46E-04
CNN3	uc001dqz.4,uc010otv.2,uc010otx.2	-4.247	1.71E-06	1.47E-04
SLC7A5	uc002fkm.3	2.430	1.77E-06	1.52E-04
NPDC1	uc004cks.2,uc004ckt.2	3.528	1.94E-06	1.65E-04
KIF19	uc002jkj.2,uc002jkk.2,uc002jkl.2,uc002jkm.4	3.585	1.96E-06	1.66E-04
GPR68	uc001xzg.3,uc001xzh.3,uc021ryk.1	4.029	2.04E-06	1.72E-04
FAM43A	uc003fuj.3	2.962	2.08E-06	1.75E-04

FHIT	uc003dkx.4,uc003dky.3,uc010hnn.1	-2.693	2.16E-06	1.81E-04
SLC35F2	uc001pjq.3,uc001pjs.3,uc010rvu.2	1.875	2.27E-06	1.89E-04
F5	uc001ggg.1,uc010plr.1	2.235	2.48E-06	2.06E-04
WDR86-AS1	uc003wkd.3,uc022aqd.1	3.818	2.49E-06	2.06E-04
RNF144A	uc002qys.3,uc002qyu.3	-1.687	2.63E-06	2.16E-04
TPX2	uc002wwp.1,uc010gdv.1	3.110	2.66E-06	2.18E-04
C12orf75	uc001tlh.4,uc031qji.1	1.785	2.81E-06	2.29E-04
SPON1	uc001mle.3	2.242	2.84E-06	2.30E-04
PPP4R1	uc002kod.2,uc002koe.2,uc002kof.3,uc010wzo.2	1.805	2.83E-06	2.30E-04
GPRIN1	uc003meo.1,uc021yif.1	4.146	2.90E-06	2.33E-04
RBBP8	uc002ktw.3,uc002ktx.1,uc002kty.3,uc002ktz.3,uc002kua.3,uc010xap.2,uc021uid.1	2.513	2.92E-06	2.34E-04
TXNRD3	uc003ejd.2,uc011bkl.2	-4.849	2.97E-06	2.37E-04
SLC40A1	uc002uqp.4,uc002uqq.2,uc002uqr.1,uc002uqs.1,uc010zfx.1	-1.592	2.98E-06	2.37E-04
RGS3	uc004bhq.4,uc004bhr.4,uc004bhs.4,uc004bht.4,uc004bhu.3,uc004bhv.4,uc004bhw.4,uc004bhy.1,uc004bhz.4,uc004bia.4,uc010muy.4,uc010muz.2,uc011lxh.3,uc031teu.1,uc031tev.1	1.861	2.99E-06	2.37E-04
ZBTB38	uc003etw.3,uc003ety.3,uc010hun.3,uc010huo.3,uc010hup.3,uc021xes.1	1.542	3.25E-06	2.57E-04
GPC3	uc004exe.2,uc010nrn.2,uc010nro.2,uc010nrp.2,uc011mvh.2	-19.000	3.38E-06	2.65E-04
CCL5	uc002hkf.3	3.256	3.44E-06	2.69E-04
DHRS3	uc001aub.3,uc001auc.3,uc001aud.4,uc001aue.1,uc009vnm.3	-1.855	3.77E-06	2.94E-04
KLRB1	uc010sgt.2	3.610	4.00E-06	3.11E-04
PROSER2	uc001ikx.3	-4.559	4.08E-06	3.16E-04
EVC	uc003gil.1,uc003gim.1	4.801	4.13E-06	3.18E-04
RILPL2	uc001uey.1	1.688	4.23E-06	3.24E-04
MMP11	uc002zxx.3,uc002zxy.3	-4.486	4.23E-06	3.24E-04
NME4	uc002cgz.3,uc021szp.1	-1.947	4.36E-06	3.32E-04
C14orf64	uc001yfw.2,uc001yfx.2,uc001yfy.2,uc010avq.2	-1.610	4.59E-06	3.48E-04
TOX2	uc002xle.4,uc002xlf.4,uc010ggo.3,uc010ggp.3,uc010zwk.2,uc021wdz.2,uc031rtm.1	2.716	5.05E-06	3.82E-04
ALCAM	uc003dvv.3,uc003dvw.2,uc003dvx.3,uc003dvy.3,uc003dvz.3,uc010hpp.3,uc011bhh.1	2.142	5.08E-06	3.82E-04
CAPG	uc002spl.2,uc002spm.2,uc010fgi.2,uc010fgj.1	1.709	5.40E-06	4.05E-04
SLAMF1	uc001fwl.4,uc001fwm.3,uc010pjk.2,uc010pjl.2,uc010pjm.2	1.580	5.85E-06	4.37E-04
SOGA3	uc003qbd.3	2.069	5.87E-06	4.37E-04
SLC22A17	uc001wjl.3,uc001wjm.3,uc001wjn.3,uc010akk.3,uc010akl.1	-2.277	6.05E-06	4.48E-04
CCR9	uc003coz.2,uc003cpa.2,uc010hiv.2,uc021wwv.1	3.875	6.12E-06	4.52E-04
MS4A6A	uc001noq.3,uc001not.3,uc009ymv.3,uc010rla.2,uc010rlb.2	6.355	6.39E-06	4.70E-04
ACOT9	uc004dan.3,uc004dao.3,uc004dap.3,uc004dar.3,uc004dat.1,uc011mjt.2	1.690	7.02E-06	5.15E-04
LGR6	uc001gxu.3,uc001gxv.3,uc001gxw.3,uc009xab.3,uc009xac.1	19.000	7.12E-06	5.19E-04
ALDH7A1	uc003ktv.3,uc003ktx.3,uc011cxa.2	-3.876	7.52E-06	5.47E-04
CEP128	uc001xux.2,uc001xuy.1,uc001xva.1,uc010asz.3,uc010ata.1,uc031qpo.1	1.763	7.68E-06	5.56E-04
MS4A1	uc001npp.3,uc001npq.3,uc009ymy.1,uc009ymz.3,uc009yna.3,uc010rlc.2	6.904	7.77E-06	5.61E-04
ANKRD36BP2	uc010fhg.4	-4.703	7.95E-06	5.71E-04
ATP2B1	uc001tbf.3,uc001tbg.3,uc001tbh.3,uc009zsr.3	1.619	8.09E-06	5.79E-04

GLCCI1	uc003srk.4	1.489	8.39E-06	5.99E-04
LOC100506469	uc002oag.3,uc021usq.1	-1.586	8.49E-06	6.04E-04
PLCG2	uc002fgt.3,uc010chg.1	5.948	8.63E-06	6.11E-04
CDC42EP3	uc002rqi.2,uc021vfz.1,uc031rny.1,uc031rnz.1,uc031roa.1	1.573	8.83E-06	6.24E-04
LRFN4	uc001ojq.1,uc001ojr.3	4.317	9.01E-06	6.29E-04
LIMS3	uc002tff.3,uc021vlz.1	2.589	8.95E-06	6.29E-04
MYBL1	uc003xwj.3,uc003xwk.3,uc003xwl.3,uc003xwm.3	2.991	8.99E-06	6.29E-04
CENPV	uc002gpw.3	-2.248	9.07E-06	6.31E-04
CAPN2	uc001hob.4,uc001hoc.3,uc010puy.2	1.425	9.12E-06	6.32E-04
MYO1E	uc002aga.4	19.000	9.93E-06	6.86E-04
PRC1	uc002bqm.4,uc002bqn.4,uc010uqs.3,uc010uqt.2	2.410	1.00E-05	6.89E-04
ACTN4	uc002oja.2,uc010egc.2,uc021uug.1	2.355	1.07E-05	7.32E-04
TIPARP	uc003fav.3,uc003faw.3,uc021xgg.1	2.562	1.07E-05	7.32E-04
LINC00282	uc001vft.1,uc010tgq.1	-3.022	1.16E-05	7.90E-04
GLIPR1	uc001sxs.3,uc009zsb.1	1.576	1.23E-05	8.36E-04
RAB27A	uc002aco.3,uc002acp.3,uc002acq.3,uc002acr.3	1.508	1.25E-05	8.47E-04
FAM124B	uc002vnw.3,uc002vnx.3	19.000	1.26E-05	8.50E-04
AGFG2	uc003uvf.3,uc003uvg.1	1.527	1.31E-05	8.77E-04
FAAH2	uc004dvc.3	1.639	1.40E-05	9.35E-04
GSAP	uc003ugd.3,uc003ugf.3,uc003ugg.1,uc011kgo.2	-1.483	1.45E-05	9.67E-04
AK124066	uc001stv.1	2.628	1.56E-05	1.04E-03
ACSS2	uc002xbc.2,uc002xbd.2,uc002xbe.2,uc010gey.2,uc010zum.1	-1.855	1.61E-05	1.06E-03
EPHX2	uc003xfu.4,uc003xfv.4,uc010lut.2,uc010luw.4,uc011lam.1,uc031tap.1	-1.650	1.63E-05	1.08E-03
PDE9A	uc002zbm.3,uc002zbn.3,uc002zbo.3,uc002zbp.3,uc002zbq.3,uc002zbr.3,uc002zbs.3,uc002zbt.3,uc002zbu.3,uc002zbv.3,uc002zbw.3,uc002zbx.3,uc002zby.3,uc002zbz.3,uc002zca.3,uc002zcb.3,uc002zcc.3,uc002zcd.3,uc002zce.3,uc002zcf.3,uc002zcg.3,uc010gpf.1	-2.717	1.66E-05	1.09E-03
CYP2J2	uc001czq.3	-5.159	1.69E-05	1.10E-03
SYDE2	uc001dku.4,uc009wcm.3	-2.003	1.70E-05	1.11E-03
PHTF2	uc003ugo.4,uc003ugp.3,uc003ugq.4,uc003ugr.4,uc003ugs.4,uc003ugt.4,uc003ugu.4,uc003ugv.3,uc010ldv.3,uc010ldw.2,uc022agp.1	1.588	1.71E-05	1.11E-03
C16orf45	uc002ddo.3,uc002ddp.3	2.614	1.72E-05	1.12E-03
ARHGAP11B	uc001zet.1,uc001zeu.3,uc010azv.1	3.072	1.74E-05	1.13E-03
ANXA1	uc004ajf.1,uc004ajg.1,uc031tee.1	1.431	1.80E-05	1.16E-03
FASLG	uc001gis.3,uc001git.3	5.093	1.83E-05	1.18E-03
FGR	uc001boi.3,uc001boj.3,uc001bok.3,uc001bol.3,uc001bom.3,uc021ojx.1	4.313	1.85E-05	1.18E-03
F2R	uc003ken.4	4.013	1.91E-05	1.22E-03
GPRC5B	uc002dgt.3,uc010vav.2,uc021tef.1	-3.545	1.99E-05	1.27E-03
LOC100132111	uc010pdr.2	4.613	2.09E-05	1.32E-03
OXNAD1	uc003caw.3,uc003cax.3,uc010her.2,uc011awb.2	-1.392	2.09E-05	1.32E-03
LOC100506190	uc004bxv.3,uc004bxw.3,uc004bxx.3,uc004bxy.2,uc004bxz.2	1.837	2.08E-05	1.32E-03
TIFA	uc003ial.3,uc021xqt.1	1.617	2.12E-05	1.33E-03
SMAD5	uc003lbj.1,uc003lbk.1,uc003lbl.1	1.843	2.15E-05	1.35E-03
OBSCN	uc001hsn.4,uc001hso.3,uc001hsp.2,uc001hsq.2,uc001hsr.1,uc009xez.2,uc	-1.456	2.25E-05	1.40E-03

	009xfa.3			
TMIGD2	uc002lzx.2,uc010dtv.2	-3.481	2.36E-05	1.46E-03
CLIC3	uc004ckj.1	3.678	2.37E-05	1.47E-03
LIMS3	uc002tfw.3	2.425	2.56E-05	1.57E-03
MXD4	uc003geu.1,uc003gev.1,uc021xko.1,uc021xkp.1	1.378	2.56E-05	1.57E-03
TMEM64	uc003yen.2,uc003yeo.2,uc011lgf.1	1.595	2.55E-05	1.57E-03
MAML3	uc011chd.1,uc021xsg.1	-3.989	2.70E-05	1.65E-03
MYO5A	uc002abx.3,uc002aby.2,uc002abz.1,uc002aca.1,uc002acb.1,uc002acc.1,uc010ugd.1,uc010uge.1	1.538	2.84E-05	1.73E-03
RCBTB2	uc001vch.3,uc001vci.3,uc001vcj.3,uc010acv.1,uc010tgg.2,uc010tgh.2,uc010tgi.1	1.478	2.86E-05	1.74E-03
HOOK1	uc001czo.3,uc001czp.3,uc009wad.3,uc010oor.2	-1.475	3.11E-05	1.88E-03
EZR	uc003qrt.4,uc003qru.4,uc011efr.2,uc011efs.2	1.305	3.15E-05	1.90E-03
LAG3	uc001qqs.3,uc001qqt.4,uc001qqu.3	3.168	3.20E-05	1.93E-03
PLCD1	uc003chm.3,uc003chn.3	1.442	3.21E-05	1.93E-03
RNF19A	uc003yjj.1,uc003yjk.2,uc003yjl.1	1.497	3.30E-05	1.98E-03
TUBB4B	uc004cmg.1,uc004cmh.1	1.529	3.43E-05	2.05E-03
NDC80	uc002kli.3	2.085	3.52E-05	2.10E-03
MKI67	uc001lke.3,uc001lkf.3,uc009yav.1,uc009yaw.1	2.880	3.54E-05	2.10E-03
SLC2A8	uc004bqu.4,uc010mxj.4,uc031tfc.1,uc031tfd.1	19.000	3.67E-05	2.17E-03
LEF1	uc003hyt.2,uc003hyu.2,uc003hyv.2,uc003hyw.1,uc010imb.2,uc011cfj.1,uc011cfk.2	-1.369	3.77E-05	2.22E-03
GZMH	uc001wpr.2,uc010aly.2,uc010alz.2,uc031qoe.1	4.837	3.79E-05	2.23E-03
TPM4	uc002ndi.2,uc002ndj.2,uc002ndk.1,uc002ndl.1	1.385	3.84E-05	2.25E-03
ROBO3	uc001qbc.3,uc001qbd.2,uc001qbe.3,uc001qbf.1,uc010saq.2,uc010sar.2	-2.183	3.86E-05	2.26E-03
KRT2	uc001sat.3	-5.050	3.95E-05	2.30E-03
NEK3	uc001vgh.3,uc001vgi.3,uc010tgx.2,uc010tgy.2	1.912	4.05E-05	2.35E-03
HLA-DPA1	uc003ocs.2,uc010juk.3,uc021ywg.1,uc021ywh.1	2.377	4.37E-05	2.53E-03
SNX24	uc003ktf.2,uc010jcy.3,uc011cwo.2	2.616	4.49E-05	2.59E-03
BUB1B	uc001zkx.4,uc010ucl.1	4.959	4.55E-05	2.62E-03
LOC100505761	uc001kvp.2,uc001kvq.2	-1.527	4.57E-05	2.62E-03
STAM	uc001ipj.2,uc009xjw.2,uc010qcf.2	1.341	4.64E-05	2.66E-03
MOCS1	uc003opa.3,uc003opb.3,uc003opd.3,uc003ope.3	-5.039	4.71E-05	2.69E-03
LEF1-AS1	uc021xqn.1	-4.740	4.79E-05	2.72E-03
DLG3	uc004dyi.2,uc004dyj.2,uc011mpn.2	1.604	4.78E-05	2.72E-03
DQ658414	uc003lyl.4,uc021yhe.1	4.254	4.93E-05	2.79E-03
IKZF3	uc002hsu.4,uc002hsv.4,uc002hsw.4,uc002hsx.4,uc002hsy.4,uc002hsz.4,uc002hta.4,uc002htb.4,uc002htc.4,uc002htd.4,uc010cwd.4,uc010cwe.4,uc010cwf.4,uc010cwg.4,uc010cwh.4,uc010wel.3	1.428	5.10E-05	2.88E-03
GNA15	uc002lxf.2,uc010xhf.1	4.587	5.14E-05	2.89E-03
ATP6V0A1	uc002hzp.1,uc002hzq.3,uc002hzr.3,uc002hzs.3,uc002hzt.3,uc010cyg.3,uc010wgj.2,uc010wgk.2,uc010wgl.2	-1.697	5.19E-05	2.90E-03
IQGAP2	uc003kek.3,uc003kel.3,uc010izv.2,uc010izw.1,uc011csv.2	1.430	5.18E-05	2.90E-03
VANGL1	uc001efv.1,uc009wgy.1,uc021ose.1	1.988	5.28E-05	2.94E-03
PDE3B	uc001mlm.2,uc001mln.3,uc010rcr.2	-1.277	5.28E-05	2.94E-03
WHAMMP2	uc001zci.1,uc010azf.3,uc010azg.1,uc010azh.1,uc010azi.1,uc010uap.2	-1.569	5.36E-05	2.97E-03

HK2	uc002snd.3	-1.714	5.92E-05	3.27E-03
ITGA4	uc002unu.3,uc002unv.3,uc010frj.1,uc010zfl.1	1.554	6.05E-05	3.34E-03
NIPAL2	uc003yil.1,uc003yim.1,uc011lgw.1	5.002	6.10E-05	3.35E-03
EFHC2	uc004dgb.4,uc022bvg.1	-2.874	6.15E-05	3.37E-03
PAK3	uc004eoy.2,uc004eoz.2,uc004epa.2,uc010npt.1,uc010npu.1,uc010npv.1,uc010npw.1,uc011mst.1	4.239	6.18E-05	3.38E-03
MLF1	uc003fbx.3,uc003fby.3,uc003fbz.3,uc003fca.3,uc003fcb.3,uc003fcc.3,uc010hvx.3	3.325	6.20E-05	3.38E-03
PLEKHG1	uc003qny.1,uc003qnz.2,uc011eel.1,uc011eem.1	4.006	6.22E-05	3.38E-03
CD55	uc001hfq.4,uc001hfr.4,uc009xce.3,uc009xcf.3,uc010psf.2,uc031prz.1	-1.422	6.42E-05	3.47E-03
LRP6	uc001rah.4,uc010shl.1	-3.442	6.43E-05	3.47E-03
IL7	uc003ybg.3,uc003ybh.3,uc003ybi.3,uc022awh.1,uc022awi.1,uc022awj.1	6.782	6.56E-05	3.53E-03
ZNRF1	uc002fdk.3,uc002fdl.1,uc010cgr.1,uc010vmz.1	2.044	6.58E-05	3.54E-03
SRGAP2	uc001hdy.3,uc009xbt.4,uc010prt.1,uc010pru.2,uc010prv.1,uc031pry.1	1.644	6.63E-05	3.55E-03
ARHGAP11A	uc001zgw.3,uc001zgy.1,uc010ubw.1,uc010ubx.1	1.786	6.64E-05	3.55E-03
EVPL	uc002jqi.2,uc010wss.1,uc010wst.1	-19.000	6.66E-05	3.55E-03
REG4	uc001eif.3,uc001eig.3,uc001eih.1	-19.000	6.70E-05	3.55E-03
S100A4	uc001fby.3,uc001fbz.3	1.908	6.70E-05	3.55E-03
TMX4	uc002wmx.1	1.281	6.72E-05	3.55E-03
REEP3	uc001jmt.3,uc009xpl.2	1.475	6.76E-05	3.56E-03
GSE1	uc002fiw.3,uc002fix.4,uc002fiy.4,uc002fiz.3,uc010cho.3	1.687	7.00E-05	3.68E-03
PSEN2	uc001hqk.2,uc009xeo.1,uc009xep.1	3.798	7.21E-05	3.77E-03
ADPRH	uc003ecs.3,uc003ect.3,uc010hqv.3,uc011bjb.2	4.433	7.21E-05	3.77E-03
EFCAB13	uc002ill.1,uc002ilm.3,uc002iln.3,uc010daz.1	-2.328	7.31E-05	3.81E-03
CEP55	uc001kiq.4,uc009xug.3	19.000	7.38E-05	3.84E-03
ZSCAN18	uc002qrh.2,uc002qri.2,uc002qrj.3,uc002qrk.1,uc002qrl.2,uc002qrm.2,uc010yhs.1,uc010yht.1	-1.598	7.43E-05	3.85E-03
KIF11	uc001kic.3	1.804	7.52E-05	3.86E-03
CHST11	uc001tky.3,uc001tkz.3	1.394	7.49E-05	3.86E-03
MCF2L2	uc003fli.1,uc003flj.1,uc003flp.1,uc011bqr.1,uc011bqs.1	2.120	7.52E-05	3.86E-03
RCAN2	uc003oyb.2,uc003oyc.2,uc003oyd.2	19.000	7.47E-05	3.86E-03
MYO6	uc003pig.1,uc003pih.1,uc003pii.1,uc003pij.1	1.930	7.96E-05	4.07E-03
TNK1	uc002ggi.4,uc002ggj.4,uc010cmf.3	-1.727	8.26E-05	4.21E-03
RAB34	uc002hce.2,uc002hcg.2,uc002hch.2,uc010was.1,uc010wat.1,uc010wau.1,uc010wav.1,uc031qzp.1	-2.951	8.40E-05	4.27E-03
FAM63A	uc001ewc.3,uc001ewd.3,uc001ewf.3,uc001ewg.3,uc010pcm.2,uc010pcn.2	-1.618	8.58E-05	4.36E-03
SH3RF3	uc010ywt.1	-1.809	8.68E-05	4.39E-03
ARAP2	uc003gso.3,uc003gsq.2,uc003gsr.1	1.721	8.79E-05	4.44E-03
ME3	uc001pbz.3,uc001pca.3,uc009yvk.3,uc010rtr.1	-2.862	8.83E-05	4.45E-03
RNF144B	uc003ncs.3	-2.400	8.97E-05	4.50E-03
FAM45B	uc001ldw.3,uc010qsv.2,uc010qsw.2,uc010qsx.2,uc010qsy.2	1.418	9.07E-05	4.54E-03
KLRG1	uc001qvg.3,uc001qvh.3	1.682	9.10E-05	4.55E-03
TESC	uc001twh.3,uc001twi.3,uc021rem.1	3.397	9.24E-05	4.59E-03
JX073282	uc031qqg.1	-3.241	9.23E-05	4.59E-03

CD226	uc002lkm.4,uc010dqo.3,uc021uli.1	1.470	9.31E-05	4.62E-03
CCR7	uc002huw.3	-1.425	9.68E-05	4.79E-03
FAM179A	uc002rmr.4,uc002rms.1,uc010ezl.3,uc010ymm.2	3.332	1.00E-04	4.94E-03
PFKP	uc001igp.3,uc001igq.3,uc009xhr.3,uc009xhs.1,uc009xht.3,uc009xhu.3	1.334	1.01E-04	4.96E-03
RCN3	uc002poj.3	-2.134	1.01E-04	4.96E-03
PLEKHA5	uc001rdz.4,uc001rea.3,uc001reb.3,uc001rec.1,uc009zin.3,uc009zio.3,uc010sie.2,uc010sig.2,uc010sih.1,uc031qgo.1	1.797	1.01E-04	4.96E-03
NCAPH	uc002svz.1,uc010fhu.1,uc010fhv.1,uc010yum.1,uc010yun.1	5.920	1.05E-04	5.12E-03
MPZL2	uc001psn.3,uc001pso.3	-3.937	1.05E-04	5.13E-03
TUBA1C	uc001rtt.1,uc010smh.1,uc021qxo.1	1.656	1.08E-04	5.23E-03
PTTG1	uc003lyj.3,uc003lyk.3	1.828	1.08E-04	5.23E-03
AKAP6	uc001wrq.3,uc010aml.3	-3.956	1.10E-04	5.32E-03
RELB	uc021uvp.1,uc021uvq.1	1.587	1.15E-04	5.55E-03
KIF21A	uc001rlt.3,uc001rlu.3,uc001rlv.3,uc001rlw.3,uc001rlx.3,uc001rly.3,uc001rlz.3,uc010skl.2	1.592	1.17E-04	5.58E-03
ATP9A	uc002xwf.1,uc002xwg.1,uc010gih.1	19.000	1.16E-04	5.58E-03
APOBEC3C	uc003awr.3	1.986	1.17E-04	5.58E-03
GALNT6	uc001ryj.1,uc001ryk.2,uc001ryl.1,uc009zma.1,uc010snh.1	-1.752	1.20E-04	5.72E-03
DYRK1B	uc002omi.3,uc002omj.3,uc002omk.3,uc002oml.3	1.669	1.22E-04	5.79E-03
BLVRA	uc003tir.3,uc010kxv.3	1.422	1.22E-04	5.79E-03
SKAP2	uc003syc.3,uc011jzi.2,uc011jzj.2	1.886	1.25E-04	5.94E-03
KIAA1671	uc003abl.3,uc003abn.3	2.082	1.26E-04	5.97E-03
SERPINB8	uc002ljs.1,uc002ljt.3,uc002lju.3,uc002ljv.3,uc010xex.2	1.609	1.28E-04	6.05E-03
PRSS23	uc001pcb.3,uc001pcc.1,uc010rts.1,uc021qok.1	19.000	1.33E-04	6.26E-03
C1orf21	uc001gqv.1	19.000	1.35E-04	6.28E-03
PDIA6	uc002rau.3,uc002rav.3,uc002raw.3,uc010yjg.2,uc010yjh.2	1.249	1.34E-04	6.28E-03
CXCL10	uc003hjl.4	19.000	1.34E-04	6.28E-03
OGDH	uc003tlm.3,uc003tln.3,uc003tlo.1,uc003tlp.3,uc011kbx.2,uc011kby.2,uc011kbz.2	1.951	1.37E-04	6.37E-03
ARHGAP10	uc003ile.1,uc003ilf.3,uc003ilg.3,uc003ilh.3,uc003ili.3	1.901	1.38E-04	6.43E-03
PHTF1	uc001edm.2,uc001edn.3,uc001edo.1,uc009wgp.1	2.992	1.41E-04	6.55E-03
TSPAN2	uc001eft.3,uc021osc.1	1.622	1.42E-04	6.55E-03
SH2D2A	uc001fqc.1,uc001fqd.2,uc001fqe.2,uc009wsh.2,uc010phs.1	1.635	1.42E-04	6.55E-03
TRIB1	uc003yrx.3,uc010mdn.3,uc011lis.2,uc022bay.1	3.816	1.42E-04	6.55E-03
NBEA	uc001uvd.3,uc010abi.3,uc010tee.1,uc010tef.2,uc010teg.1,uc021ric.1,uc021rid.1	-3.947	1.48E-04	6.81E-03
DQ572107	uc002jdy.1	1.364	1.53E-04	7.00E-03
PLEKHA1	uc001lge.2,uc001lgf.2,uc001lgg.2,uc021qae.1	-1.278	1.53E-04	7.00E-03
SLC9A3R1	uc002jln.1,uc002jlo.4,uc021ucr.1	1.150	1.54E-04	7.02E-03
B4GALT4	uc003ece.1,uc003ecg.3,uc003ech.3,uc003eci.3,uc011biy.2	-1.519	1.57E-04	7.13E-03
HRH2	uc003mdc.4,uc003mdd.2	-2.254	1.57E-04	7.13E-03
APOBR	uc002dqb.2,uc010byg.2	1.843	1.65E-04	7.49E-03
DPYSL2	uc003xfa.3,uc003xfb.2,uc011lag.2,uc011lah.2	1.439	1.73E-04	7.83E-03
FAM134B	uc003jfr.3,uc003jfs.3	-1.403	1.78E-04	8.00E-03

HLF	uc002iug.1,uc002iuh.2,uc010dce.1,uc010wni.1	3.194	1.79E-04	8.07E-03
SLAMF7	uc001fwq.3,uc001fwr.3,uc001fws.3,uc010pjn.2,uc010pjo.2,uc010pjp.2,uc010pjq.2,uc010pjr.2	4.023	1.80E-04	8.09E-03
PRKCA	uc002jfo.1,uc002jfp.1	-1.321	1.82E-04	8.15E-03
DACH1	uc021rkj.1,uc021rkk.1,uc021rkl.1	-19.000	1.91E-04	8.54E-03
PLEKHO2	uc002anv.3,uc002anw.3	1.211	1.94E-04	8.66E-03
VIPR1	uc003clf.2,uc003clg.2,uc011azl.1,uc011azm.1,uc011azn.2,uc021wwl.1	-1.291	1.98E-04	8.81E-03
LOC642852	uc002zhf.3,uc031rvz.1	-1.673	2.00E-04	8.87E-03
KIAA1324	uc001dwr.3,uc001dws.1,uc009wex.2,uc009wey.3,uc009wez.1,uc010ovg.3,uc021orb.1,uc031pnl.1	1.437	2.06E-04	9.09E-03
CCDC64	uc001txk.2,uc001txl.1,uc009zwv.1,uc010sze.1,uc010szf.1	1.321	2.06E-04	9.09E-03
LOC100507387	uc003mdl.2,uc003mdm.1	-2.084	2.06E-04	9.09E-03
TTC38	uc003bhi.3,uc011aqx.2	1.764	2.10E-04	9.22E-03
TNFRSF10D	uc003xcz.2	-1.310	2.13E-04	9.35E-03
IFNGR2	uc002yrp.4	-1.303	2.23E-04	9.75E-03
SOCS2	uc001tcw.2,uc001tcy.2,uc021rbx.2,uc031qja.1,uc031qjb.1,uc031qjc.1	-1.420	2.23E-04	9.75E-03
GP5	uc003ftv.1,uc021xiz.1	-2.156	2.28E-04	9.90E-03
OCRL	uc004euq.3,uc004eur.3,uc010nrb.3	-2.411	2.28E-04	9.90E-03
MAP4	uc003crw.2,uc003crx.2,uc003crz.4,uc003csa.3,uc003csb.2,uc003csc.3,uc003csd.2,uc003cse.1,uc003csf.3,uc003csg.3,uc011bbe.1,uc011bbf.1	1.185	2.30E-04	9.97E-03
IQGAP1	uc002bpl.1,uc010uqg.1	1.263	2.34E-04	1.01E-02
COL5A3	uc002mmq.1	19.000	2.34E-04	1.01E-02
FABP5	uc003yca.2	1.700	2.36E-04	1.02E-02
CHST12	uc003smc.3,uc003smd.3,uc021zyu.1,uc021zyv.1	1.586	2.38E-04	1.02E-02
B4GALT5	uc002xuu.4	2.464	2.40E-04	1.03E-02
TRIM16	uc002gor.1,uc002gou.2,uc002gov.4,uc002gow.3,uc002gox.3,uc002goy.3,uc010vvy.2,uc010vvz.2	1.678	2.42E-04	1.04E-02
LINC00880	uc003fbc.3	3.246	2.44E-04	1.04E-02
CD200	uc003dyw.3,uc003dyx.3,uc003dyy.3,uc003dyz.3,uc010hqd.1	1.931	2.45E-04	1.04E-02
LOC286467	uc004ewi.3,uc004ewj.1	-2.343	2.45E-04	1.04E-02
PDCD1LG2	uc003zjg.4,uc010mho.1,uc010mhp.1,uc011lmc.2,uc011lmd.2	19.000	2.51E-04	1.07E-02
PTGIR	uc002pex.3	-5.702	2.52E-04	1.07E-02
MRC1	uc001ipk.4,uc001ipm.4,uc031ptj.1	19.000	2.53E-04	1.07E-02
PHF21A	uc001nca.1,uc001ncb.4,uc001ncc.4,uc001nce.2,uc009ykx.3	1.359	2.63E-04	1.10E-02
RIN3	uc001yap.3,uc001yaq.3,uc001yar.1,uc001yas.1,uc010auk.3	-1.477	2.63E-04	1.10E-02
GSR	uc003xih.2,uc022ato.1,uc022atp.1,uc022atq.1	1.223	2.70E-04	1.13E-02
LLGL2	uc002jog.1,uc002joh.3,uc002joi.3,uc002joj.3,uc010dgf.1,uc010dgg.2,uc010wsd.2	1.174	2.70E-04	1.13E-02
SORCS3	uc001kyi.1,uc010qqz.1	-4.311	2.72E-04	1.14E-02
KBTBD8	uc003dmy.3,uc011bfv.2	2.037	2.73E-04	1.14E-02
LPHN1	uc010xnn.2,uc010xno.2	-2.205	2.77E-04	1.15E-02
MAP4K1	uc002oix.1,uc002oiy.1,uc010xug.2	1.124	2.79E-04	1.15E-02
CD274	uc003zje.3,uc003zjf.4,uc010mhn.4,uc011lmb.2	1.724	2.78E-04	1.15E-02
GLB1	uc003cfh.1,uc003cfi.1,uc003cfj.1,uc011axk.1	1.268	2.82E-04	1.16E-02
NPCR	uc003dkz.3	-4.166	2.91E-04	1.20E-02

PPP1R26	uc004cfr.1,uc022bpi.1	5.864	2.93E-04	1.20E-02
GPR183	uc001vog.3,uc021rma.1	1.178	2.95E-04	1.21E-02
ZNF516	uc002lmd.3,uc021ulp.1	-2.938	3.01E-04	1.23E-02
CLIC1	uc003nwr.3	2.303	3.05E-04	1.25E-02
CCR5	uc003cpo.4,uc010hjd.3,uc021wxb.1	4.775	3.09E-04	1.26E-02
PPAP2A	uc003jpz.4,uc003jqa.4,uc003jqb.4	-1.547	3.17E-04	1.29E-02
LGALS9	uc002gzp.3,uc002gzq.3,uc002gzr.3,uc010waa.2	1.809	3.20E-04	1.30E-02
SQRDL	uc001zvu.4,uc001zvv.4	1.201	3.24E-04	1.31E-02
PLEKHG3	uc001xhn.1,uc001xho.1,uc001xhp.2,uc001xhq.1,uc010aqh.1	3.991	3.26E-04	1.32E-02
GRB10	uc003tph.3,uc003tpi.2,uc003tpj.2,uc003tpk.2,uc003tpl.2,uc003tpm.2,uc010kzb.2	-1.580	3.30E-04	1.33E-02
NUAK2	uc001hce.3,uc009xbj.1	-1.234	3.32E-04	1.34E-02
VCL	uc001jwd.3,uc001jwe.3,uc009xrr.3,uc010qky.1,uc010qkz.2	1.178	3.33E-04	1.34E-02
NAGA	uc003bbw.4	1.165	3.37E-04	1.35E-02
HHAT	uc001hhz.4,uc001hia.4,uc009xcx.3,uc009xcy.3,uc010psq.2,uc010psr.2,uc010pss.2,uc010pst.2,uc010psu.2,uc021pip.1	1.638	3.42E-04	1.37E-02
FAM53B	uc001lhu.1,uc001lhv.1,uc001lhw.3	1.378	3.43E-04	1.37E-02
RAD51	uc001zmi.4,uc001zml.4,uc001zmm.1,uc001zmn.1,uc010bbw.3,uc010bbx.3	2.499	3.43E-04	1.37E-02
EPS15	uc001csp.3,uc001csq.1,uc009vyz.1	1.151	3.44E-04	1.37E-02
LOC729041	uc021ona.1	3.486	3.47E-04	1.38E-02
APBB1	uc001mdb.2,uc001mdc.1,uc001mdd.4,uc001mde.3,uc009yey.3,uc009yfa.3,uc009yfb.3,uc010rab.2,uc010rad.2,uc010rag.2,uc010rah.2,uc031pyt.1,uc031pyu.1,uc031pyv.1	-1.268	3.50E-04	1.38E-02
AX748314	uc002frx.1	-1.939	3.49E-04	1.38E-02
TTL	uc002thu.3	1.288	3.50E-04	1.38E-02
ATHL1	uc001lor.4,uc001los.1,uc001lou.4,uc001lov.4,uc010qvu.2	-1.263	3.52E-04	1.39E-02
AMN	uc001ymg.4,uc001ymh.4	-6.261	3.53E-04	1.39E-02
TCEA3	uc009vqm.2,uc010ody.1,uc021oig.1,uc021oih.1	-1.318	3.56E-04	1.40E-02
RNF157	uc002jqz.3,uc002jra.3	-1.455	3.58E-04	1.40E-02
CITED4	uc001cgj.3	-2.207	3.74E-04	1.46E-02
MAP3K8	uc001ivi.2,uc001ivj.2,uc009xlf.2	3.644	3.75E-04	1.46E-02
DUSP6	uc001tay.3,uc001taz.3	2.268	3.78E-04	1.47E-02
DEPDC1B	uc003jsh.3,uc011cqm.2,uc011cqn.2	5.493	3.80E-04	1.48E-02
LMNA	uc001fnf.1,uc001fng.2,uc001fnh.3,uc001fni.3,uc001fnj.3,uc001fnk.3,uc009wro.2,uc010pgz.2,uc010pha.1	3.808	3.82E-04	1.48E-02
UCK2	uc001gdp.3,uc010plb.2,uc021pec.1	2.430	3.82E-04	1.48E-02
EFNA1	uc001fhh.3,uc001fhi.3,uc009wpd.1	-2.419	3.85E-04	1.48E-02
KBTBD11	uc003wpw.4,uc022aqq.1	-2.107	3.85E-04	1.48E-02
LOC100134368	uc002cgw.1	-3.695	3.87E-04	1.49E-02
CDK1	uc001jld.3,uc001jle.3,uc001jlg.3,uc010qii.2,uc021prh.1,uc031pvg.1	4.443	3.91E-04	1.50E-02
LZTS3	uc002wia.1,uc002wib.1	-2.121	3.91E-04	1.50E-02
PLEKHB1	uc001oua.3,uc001oub.3,uc001ouc.3,uc001oud.3,uc009ytq.3,uc010rrh.1	-1.321	3.96E-04	1.51E-02
PROK2	uc003doz.4,uc003dpa.4	19.000	3.97E-04	1.52E-02
PARPBP	uc001tjd.3,uc001tje.3,uc001tjf.3,uc001tjg.3,uc001tjh.3,uc001tji.3,uc001tjj.3,uc001tjk.3,uc009zuc.3,uc009zud.3,uc010swa.2,uc010swb.2	1.924	4.01E-04	1.53E-02

NFKBIE	uc003oxe.1	1.309	4.08E-04	1.55E-02
ZFP69B	uc001cfl.2,uc001cfm.2,uc001cfn.2	-2.276	4.10E-04	1.55E-02
PDCD4-AS1	uc010qrd.1	-1.623	4.14E-04	1.57E-02
TSHR	uc001xvb.1,uc001xvc.3,uc001xvd.1,uc010tvs.2	4.938	4.15E-04	1.57E-02
MXRA8	uc001aew.3,uc001aex.4,uc001aey.4,uc001aez.3,uc001afa.3	-19.000	4.22E-04	1.59E-02
EPHA4	uc002vmq.3,uc002vmr.2,uc010zlm.1	1.758	4.24E-04	1.59E-02
LOC643733	uc001phz.3,uc021qpn.1,uc021qpo.1	2.324	4.31E-04	1.61E-02
CITED2	uc003qip.2,uc021zfz.2,uc021zga.2,uc021zgb.1	1.343	4.30E-04	1.61E-02
SPPL2A	uc001zyv.3	1.229	4.37E-04	1.64E-02
DPPA4	uc003dxq.4,uc011bho.2,uc011bhp.1	-3.640	4.39E-04	1.64E-02
REEP5	uc003kqe.1,uc011cvw.1,uc011cvx.1,uc011cvy.1,uc011cvz.1	1.123	4.39E-04	1.64E-02
AUTS2	uc003tvv.4,uc003tvw.4,uc003tvx.4,uc011keg.2	1.984	4.40E-04	1.64E-02
PRKCQ-AS1	uc001ijl.3,uc021pmt.1	-1.585	4.44E-04	1.64E-02
DEPDC7	uc001mub.3,uc001muc.3,uc010reg.1,uc010reh.1	-19.000	4.44E-04	1.64E-02
CYFIP1	uc001yus.3,uc001yut.3,uc001yuu.3,uc001yuv.3,uc010aya.1	1.791	4.44E-04	1.64E-02
EDAR	uc002teq.4,uc010fjn.3,uc010yws.2	-2.490	4.50E-04	1.66E-02
NCAPG2	uc003wnv.1,uc003wnw.1,uc003wnx.1,uc010lqu.1,uc011kwc.1,uc011kwd.1,uc011kwe.1	1.532	4.51E-04	1.66E-02
C4orf32	uc003iah.2,uc003iai.3	-1.631	4.61E-04	1.69E-02
LAPTM4B	uc003yia.3,uc010mbg.3	-1.564	4.65E-04	1.71E-02
TRGC2	uc003tfu.3,uc003tfv.3,uc003tfw.3,uc003tfx.1,uc003tfz.1,uc003tga.1,uc003tgb.2,uc003tgc.1,uc003tgd.1,uc003tge.1,uc003tgf.1,uc003tgg.1,uc003tgh.1,uc003tgi.2,uc003tgj.1,uc003tgk.1,uc010kxi.1,uc022aby.1,uc022abz.1	2.505	4.75E-04	1.73E-02
CTSB	uc003wul.3,uc003wum.3,uc003wun.3,uc003wuo.3,uc003wup.3,uc003wuq.3,uc003wuu.3,uc010lsc.3,uc011kxl.2	1.131	4.74E-04	1.73E-02
NPC1	uc002kum.4,uc010dlu.1,uc010xaz.2,uc010xba.1	1.424	4.90E-04	1.79E-02
PCED1B-AS1	uc001rpp.2	-1.225	5.16E-04	1.88E-02
CBLL1	uc003veq.3,uc011kme.2,uc011kmf.2	1.160	5.17E-04	1.88E-02
PLS3	uc004eqd.3,uc004eqe.3,uc010nqf.3,uc010nqg.3,uc011mtf.2,uc011mtg.2,uc011mth.2,uc011mti.2,uc011mtj.2,uc011mtl.2	4.685	5.33E-04	1.93E-02
ACSF2	uc002iqu.2,uc010dbt.1,uc010wml.1,uc010wmm.1,uc010wmn.1,uc010wmo.1	2.694	5.36E-04	1.94E-02
RPH3AL	uc002fre.2,uc002frf.2,uc010cjl.2,uc010vpy.2,uc021tmx.1	-2.655	5.38E-04	1.95E-02
ZBTB32	uc002oay.3,uc002oaz.3	3.337	5.44E-04	1.96E-02
PPP1R3E	uc001wjc.2,uc031qns.1	-1.518	5.57E-04	2.00E-02
NPAS2	uc002tap.1,uc010fit.1,uc010yvt.1	-2.318	5.56E-04	2.00E-02
EOMES	uc003cdx.4,uc003cdy.4,uc010hfn.3,uc011axc.2	4.367	5.60E-04	2.01E-02
CYHR1	uc003zcv.2,uc003zcw.2,uc003zcx.2,uc003zcy.2	-1.114	5.62E-04	2.01E-02
BIK	uc003bdk.3	1.933	5.64E-04	2.02E-02
TLE2	uc002lww.3,uc010dth.3,uc010dti.3,uc010xhb.2,uc010xhc.2,uc010xhd.1	-2.134	5.67E-04	2.02E-02
S100A10	uc001ezl.3	1.084	5.71E-04	2.03E-02
E2F3	uc003ncz.2,uc003nda.2,uc021ymj.2	1.354	5.78E-04	2.05E-02
PLEKHG4	uc002eso.4,uc002esp.4,uc002esq.4,uc002esr.1,uc002ess.4,uc010cef.3,uc010ceg.3	-1.447	5.85E-04	2.08E-02
SH3TC1	uc003gkv.4,uc003gkw.4,uc003gkx.4,uc003gky.3	1.106	5.90E-04	2.09E-02
EHD4	uc001zot.3,uc001zou.2	1.344	5.98E-04	2.11E-02

GGT7	uc002xay.3,uc002xaz.1,uc002xba.1,uc010gex.3	-1.169	5.97E-04	2.11E-02
PREX1	uc002xtv.1,uc002xtw.1,uc021wer.1	3.348	5.97E-04	2.11E-02
NCOA7	uc003qae.4,uc003qaf.3,uc003qag.3,uc003qah.3,uc003qai.3,uc003qaj.3,uc003qak.3,uc010kes.3,uc010ket.3	1.261	6.12E-04	2.15E-02
SLC15A3	uc001nqn.2,uc001nqo.2	-19.000	6.30E-04	2.21E-02
PFKFB2	uc001hfg.3,uc001hfh.3,uc009xcc.3,uc010psc.2,uc010psd.2	-2.223	6.38E-04	2.24E-02
ARMCX4	uc004ehp.3,uc031tkc.1,uc031tkd.1	-1.955	6.46E-04	2.26E-02
PTPN13	uc003hpy.3,uc003hpz.3,uc003hqa.3,uc003hqb.3,uc003hqc.1	4.474	6.53E-04	2.28E-02
EEPD1	uc003tfa.3	1.522	6.53E-04	2.28E-02
SRGN	uc001joz.3,uc001jpa.1,uc021prx.1	1.368	6.58E-04	2.29E-02
SBF2	uc001mib.2,uc001mic.2,uc001mid.2,uc001mif.3,uc001mih.4	-1.214	6.61E-04	2.29E-02
LGMN	uc001yat.3,uc001yau.3,uc001yav.3,uc001yaw.3	-1.509	6.61E-04	2.29E-02
PPP1CA	uc001oku.1,uc001okv.1,uc001okw.1,uc001okx.1	1.068	6.65E-04	2.29E-02
CDCA4	uc001yqa.2,uc001yqb.2,uc021sep.1	1.245	6.65E-04	2.29E-02
TSPAN6	uc004ega.1,uc010nna.1	-2.562	6.65E-04	2.29E-02
TP53INP1	uc003yhg.3,uc003yhh.3	2.337	6.75E-04	2.32E-02
UHRF1	uc002mbo.3,uc002mbp.3,uc010duf.3,uc010xik.2	4.722	6.88E-04	2.36E-02
KCNQ1	uc001lwn.3,uc001lwo.4,uc009ydo.1,uc009ydp.2	-1.363	6.93E-04	2.37E-02
ZNF831	uc002yan.3	1.691	6.93E-04	2.37E-02
TBX21	uc002ilv.1	4.197	6.99E-04	2.38E-02
PAPSS1	uc003hyk.3,uc011cfh.1	1.051	6.98E-04	2.38E-02
DKK3	uc001mju.3,uc001mjv.3,uc001mjw.3,uc010rcf.2,uc010rcg.1	5.346	7.05E-04	2.40E-02
PCBP4	uc003dcb.2,uc003dcc.2,uc003dce.2,uc003dcf.2,uc003dcg.2,uc003dch.2,uc003dci.2,uc003dcj.2,uc003dck.2	1.043	7.04E-04	2.40E-02
SPATS2L	uc002uvn.4,uc002uvo.4,uc002uvp.4,uc002uvq.4,uc002uvr.4,uc010fst.3,uc010zhc.2	1.722	7.08E-04	2.40E-02
LIF	uc003agz.3,uc011aks.2	19.000	7.16E-04	2.42E-02
PLS1	uc003euz.3,uc003eva.3,uc010huv.3	-1.557	7.37E-04	2.49E-02
CD2	uc001egu.4,uc010owz.1,uc010oxa.1	1.061	7.42E-04	2.51E-02
CEP112	uc002jfk.3,uc002jfl.3,uc002jfm.3,uc010deo.3,uc010dep.2	5.414	7.48E-04	2.52E-02
MSC	uc003xyx.1	4.678	7.50E-04	2.52E-02
ZNF532	uc002lho.3,uc002lhp.3,uc002lhr.3,uc002lhs.3,uc010xeg.2,uc010xeh.2	2.682	7.70E-04	2.58E-02
STON1	uc002rwo.4,uc002rwp.2,uc002rws.2,uc010fbm.3,uc010yol.2,uc010yom.2,uc021vhf.1	3.674	7.83E-04	2.62E-02
OSER1-AS1	uc002xll.3,uc002xlm.3,uc002xln.3,uc021wec.1	-1.643	7.94E-04	2.66E-02
ZNF365	uc001jly.4,uc001jlz.4,uc001jma.4,uc001jmb.4,uc001jmc.2,uc001jmd.1,uc001jme.1,uc001jmf.1,uc009xpg.1	4.775	7.99E-04	2.66E-02
BATF	uc001xrr.3	1.425	7.98E-04	2.66E-02
STK39	uc002uea.3	1.602	8.03E-04	2.67E-02
CHN2	uc003szz.3,uc003taa.3,uc010kva.3,uc010kvb.3,uc010kvc.3,uc010kvd.3,uc010kve.3,uc010kvf.3,uc010kvg.3,uc010kvh.3,uc010kvi.3,uc010kvj.3,uc010kvk.3,uc010kvl.3,uc010kvm.3,uc011jzs.2,uc011jzt.2,uc011jzu.2,uc011jzv.2	2.918	8.08E-04	2.69E-02
APOBEC3H	uc021wps.1,uc021wpt.1,uc021wpu.1,uc021wpv.1	3.100	8.13E-04	2.70E-02
SPG20	uc001uvm.3,uc001uvn.3,uc001uvo.3,uc001uvp.2,uc001uvq.3,uc010ten.2	-1.253	8.18E-04	2.71E-02
DNAH6	uc002soo.3,uc002sop.3,uc002sor.3,uc002sot.3,uc010fgb.3	-2.164	8.31E-04	2.74E-02
GZMK	uc003jpl.1	4.976	8.31E-04	2.74E-02

RASSF1	uc003daa.1,uc003dab.1,uc003dac.2,uc003dad.1,uc003dae.1,uc003daf.1,uc010hlk.1,uc011bdq.1	1.080	8.49E-04	2.80E-02
CFP	uc004dig.4,uc004dih.3,uc004dii.1,uc010nhu.2	-1.202	8.51E-04	2.80E-02
PAM16	uc002cwd.3,uc002cwe.3,uc002cwf.3,uc002cwg.4,uc002cwh.4,uc002cwi.1,uc010btp.1,uc010uxh.2,uc010uxi.2,uc010uxj.1	1.099	8.58E-04	2.82E-02
MYO15B	uc010dgj.1	-2.142	8.59E-04	2.82E-02
CLECL1	uc001qwi.3,uc001qwj.3,uc031qgf.1	3.860	8.70E-04	2.85E-02
SIAH2	uc003eyi.3	1.131	8.75E-04	2.86E-02
ADHFE1	uc003xwb.4,uc003xwc.4,uc003xwd.4,uc003xwe.4,uc003xwf.4,uc011leq.1,uc011ler.1,uc011les.2	1.212	8.86E-04	2.89E-02
PTPRK	uc003qbj.3,uc003qbk.3,uc003qbl.1,uc003qbm.4,uc010kfc.3,uc010kfd.1,uc011ebu.2,uc011ebv.1	-3.525	9.05E-04	2.95E-02
BLM	uc002bpr.3,uc010bnx.3,uc010uqh.2,uc010uqi.2	1.397	9.23E-04	3.00E-02
DOK2	uc003wzx.1,uc003wzy.1,uc003wzz.1,uc010lth.1	1.063	9.24E-04	3.00E-02
DEPTOR	uc003yow.4,uc011lid.2	-19.000	9.29E-04	3.01E-02
PDGFB	uc003axe.3,uc003axf.3	1.458	9.38E-04	3.03E-02
TOP1MT	uc003yxz.4,uc003yya.4,uc010mfd.2,uc011lkd.3,uc011lke.3,uc011lkf.2	-1.193	9.50E-04	3.07E-02
CENPE	uc003hxb.1,uc003hxc.1,uc003hxd.1	2.944	9.68E-04	3.12E-02
CDC25A	uc003csh.1,uc003csi.1,uc021wxk.1,uc031rzi.1	19.000	9.71E-04	3.12E-02
ZBTB20-AS1	uc003ebo.2	19.000	9.83E-04	3.16E-02
ADAM8	uc009ybi.3,uc009ybj.2,uc010qva.2,uc010qvb.1,uc021qbe.1	1.167	9.89E-04	3.17E-02
LMO7	uc001vjt.1,uc001vju.1,uc001vjv.3,uc001vjw.1,uc001vjx.1,uc010thv.2,uc010thw.2,uc021rkq.1	-1.040	9.98E-04	3.20E-02
PKIG	uc002xmg.3,uc002xmh.3,uc002xmi.3	-2.335	1.04E-03	3.32E-02
SNTB2	uc002ewu.3,uc021tkg.1	1.194	1.04E-03	3.33E-02
SEMA4B	uc002boy.3,uc002boz.3,uc002bpa.3,uc010uqd.2	1.266	1.05E-03	3.35E-02
CADM1	uc001ppf.4,uc001pph.5,uc001ppi.4,uc001ppj.4,uc001ppk.4,uc001ppl.3,uc031qeh.1,uc031qei.1,uc031qej.1,uc031qek.1	5.169	1.05E-03	3.35E-02
CTSC	uc001pck.4,uc001pcl.4,uc001pcm.4,uc001pcn.4	1.108	1.06E-03	3.37E-02
CASP8	uc002uxn.3,uc002uxo.1,uc002uxp.1,uc002uxq.1,uc002uxr.1,uc002uxs.1,uc002uxt.1,uc002uxu.1,uc002uxv.1,uc002uxw.1,uc010ftc.1,uc010ftd.1,uc010fte.1,uc010ftf.2,uc021vuy.1,uc021vuz.1,uc021vva.1,uc031rqq.1	1.117	1.07E-03	3.38E-02
EEA1	uc001tck.3	-1.133	1.07E-03	3.38E-02
GPR56	uc002elz.1,uc002ema.1,uc002emb.2,uc002emc.2,uc002emd.2,uc002eme.2,uc002emf.2,uc002emg.3,uc010vhs.1,uc010vht.1,uc010vhu.1	3.959	1.10E-03	3.47E-02
ITGA6	uc002uho.1,uc002uhp.1,uc010fqk.1,uc010fql.2,uc010fqm.1,uc010zdy.1	-1.012	1.10E-03	3.47E-02
C17orf66	uc002hke.1,uc010wck.1,uc010wcl.1,uc010wcm.1,uc031qzv.1	19.000	1.10E-03	3.47E-02
ZC3H12D	uc003qmn.1,uc010kid.3	1.361	1.11E-03	3.48E-02
TOR3A	uc001gmd.3,uc010pnd.2	1.097	1.12E-03	3.51E-02
ZNF204P	uc011dkv.2	-1.450	1.12E-03	3.51E-02
SHMT2	uc001snf.2,uc001sng.2,uc001snh.2,uc001sni.2,uc001snj.2,uc001snk.2,uc009zpk.2,uc010srg.2,uc010srh.2,uc010sri.2,uc010srj.2	1.262	1.12E-03	3.52E-02
YPEL1	uc002zvl.3,uc002zvm.3	1.290	1.13E-03	3.53E-02
RABGAP1L	uc001gjw.3,uc001gjx.3,uc001gjy.3,uc001gjz.3,uc001gkb.4,uc001gkc.4,uc001gkd.4,uc001gke.4,uc001gkf.3,uc001gkg.3,uc001gkh.4,uc009wwq.2,uc010pmv.2	1.099	1.14E-03	3.54E-02
TTC28	uc003adp.4	-2.165	1.14E-03	3.54E-02
CLCF1	uc001okq.3,uc010rpp.2	5.179	1.15E-03	3.57E-02
GAPDH	uc001qop.2,uc021qtv.1,uc031qfw.1	0.980	1.15E-03	3.57E-02
SLCO3A1	uc002bqx.2,uc002bqy.2,uc002bqz.1,uc010boc.1	1.075	1.15E-03	3.57E-02

SMS	uc004dag.4,uc011mjq.3,uc031tgx.1	1.047	1.15E-03	3.57E-02
ZNF609	uc002ann.3,uc010bgy.3	-1.226	1.16E-03	3.59E-02
P2RY10	uc004ede.3,uc004edf.3,uc022bzl.1	1.190	1.17E-03	3.61E-02
P2RY8	uc004fpm.2,uc022cit.1	1.020	1.18E-03	3.63E-02
ADPRM	uc002gmt.3,uc002gmu.3,uc002gmv.3,uc010vvg.1	-1.166	1.21E-03	3.71E-02
ALS2CL	uc003cpx.2,uc003cpy.2,uc003cpz.2,uc003cqa.2,uc003cqb.2,uc003cqc.2	-1.546	1.22E-03	3.74E-02
LINC00263	uc001kqz.4,uc001kra.4	-3.092	1.23E-03	3.76E-02
SLC9A9	uc003evn.3,uc011bnk.2	1.241	1.23E-03	3.76E-02
TGIF1	uc002klu.3,uc002klv.3,uc002klw.3,uc002klx.3,uc002kly.3,uc002klz.3,uc0 02kma.3,uc002kmb.3,uc002kmc.3,uc010dkm.1	0.993	1.23E-03	3.76E-02
NAA16	uc001uyd.4,uc001uye.4,uc001uyf.2,uc010tfg.1	-1.022	1.24E-03	3.77E-02
ABLIM1	uc001lbz.1,uc021pyu.1,uc021pyv.1,uc021pyw.1,uc021pyx.1,uc021pyy.1, uc021pyz.1,uc021pza.1,uc021pzb.1,uc021pzc.1,uc021pzd.1,uc021pze.1,u c021pzf.1	-1.028	1.27E-03	3.86E-02
CHMP1A	uc002fnt.4,uc002fnu.4,uc002fnv.4,uc031qxm.1	1.117	1.27E-03	3.87E-02
ZNF618	uc004bib.1,uc004bic.3,uc004bid.3,uc010mvb.3,uc011lxi.2,uc011lxj.2	-4.399	1.27E-03	3.87E-02
SLC35D2	uc004awc.3,uc010msd.3,uc010msf.3	1.252	1.28E-03	3.88E-02
NUGGC	uc003xgm.4	1.575	1.28E-03	3.89E-02
RNF130	uc003mll.1,uc003mlm.1	-1.353	1.29E-03	3.89E-02
KEAP1	uc002mop.1,uc002moq.1,uc002mor.1	1.042	1.30E-03	3.92E-02
DCBLD2	uc003dtd.3,uc003dte.3,uc003dtf.1	-3.319	1.30E-03	3.92E-02
DNMT3B	uc002wyc.3,uc002wyd.3,uc002wye.3,uc002wyf.3,uc002wyg.3,uc010gee. 3,uc010gef.3,uc010geg.3,uc010geh.3,uc010ztx.1,uc010zty.1,uc010ztz.2,u c010zua.2	-4.446	1.31E-03	3.94E-02
AX747706	uc004cib.1	-4.207	1.31E-03	3.94E-02
TAGLN2	uc001fun.2,uc010piy.2,uc031pqt.1,uc031pqu.1	0.959	1.34E-03	4.01E-02
LTBP3	uc001oef.3,uc001oeg.3,uc001oeh.3,uc001oei.3,uc001oej.3,uc010roi.2,uc0 10roj.3,uc010rok.1	-1.232	1.35E-03	4.02E-02
CD7	uc002kel.1,uc010din.3,uc010wvk.1	-1.232	1.35E-03	4.02E-02
PTPRM	uc002knn.4,uc010dkv.3,uc010wzl.2	1.650	1.35E-03	4.02E-02
TPST1	uc003tuw.3,uc010kzy.2	-2.954	1.34E-03	4.02E-02
GLIPR2	uc003zyy.1,uc003zyz.3,uc003zza.3,uc010mlf.1,uc011lpj.1	0.965	1.36E-03	4.04E-02
ARHGAP32	uc001qez.3,uc001qfb.3,uc009zco.3,uc009zcp.3,uc009zcq.2	-3.741	1.36E-03	4.04E-02
EPS8	uc001rdb.3,uc009zif.3,uc009zig.3,uc010shv.2	-2.383	1.36E-03	4.04E-02
STARD4-AS1	uc021ych.1	-19.000	1.36E-03	4.04E-02
THNSL1	uc001isi.4,uc021pol.1	-1.130	1.37E-03	4.04E-02
IFI16	uc001ftf.1,uc001ftg.3,uc010pis.2,uc010pit.2	1.107	1.38E-03	4.06E-02
CHGB	uc002wmg.3,uc010zqz.2	5.258	1.39E-03	4.10E-02
YWHAQ	uc002qzx.3	1.138	1.40E-03	4.12E-02
LPCAT1	uc003jcl.3,uc003jcm.3	1.161	1.41E-03	4.14E-02
RC3H2	uc004bnc.2,uc004bnd.1,uc004bne.4,uc004bng.1,uc010mwc.1,uc011lzf.2,u c011lzg.2	1.251	1.41E-03	4.14E-02
MTHFD2	uc002skj.3,uc002skk.3,uc010yro.2,uc010yrp.3	1.149	1.42E-03	4.17E-02
KANK3	uc010dwa.3	1.466	1.43E-03	4.17E-02
ZNF780B	uc002omu.3,uc002omv.3	-1.365	1.43E-03	4.17E-02
TMEM116	uc001ttc.2,uc001ttd.2,uc001tte.2,uc001ttf.2,uc001tti.2,uc001ttj.1	1.316	1.44E-03	4.21E-02

SERTAD3	uc002onu.4,uc002onv.4,uc021uut.1	1.609	1.44E-03	4.21E-02
ERN1	uc002jdz.2	2.532	1.46E-03	4.25E-02
CST7	uc002wtx.2	5.514	1.46E-03	4.25E-02
ITGAL	uc002dyi.4,uc002dyj.4,uc010veu.1,uc010vev.2	1.021	1.47E-03	4.27E-02
C2orf81	uc010yrq.1	-2.622	1.48E-03	4.28E-02
N6AMT1	uc002ymo.2,uc002ymp.2,uc002ymq.2	-1.522	1.49E-03	4.31E-02
RAI2	uc004cyf.3,uc004cyg.3,uc004cyh.4,uc010nfa.3,uc011miy.2,uc022btl.1,uc022btm.1	-4.839	1.49E-03	4.31E-02
HELLS	uc001kjs.3,uc001kjt.3,uc001kju.3,uc009xul.3,uc009xum.3,uc009xun.3,uc009xuo.3,uc009xup.3,uc009xuq.3,uc009xur.3	2.232	1.51E-03	4.34E-02
RBM11	uc002yjn.4,uc002yjo.4,uc002yjp.4	-3.205	1.51E-03	4.34E-02
ZNF491	uc002mso.1,uc021upj.1	-1.895	1.52E-03	4.36E-02
SPON2	uc003gcm.1,uc003gco.4,uc003gcp.3,uc010ibr.3,uc021xkj.1	1.513	1.52E-03	4.36E-02
GGT1	uc003aan.1,uc003aas.1,uc003aat.1,uc003aau.2,uc003aav.2,uc003aaw.2,uc003aax.2,uc003aay.1	1.933	1.53E-03	4.38E-02
SMCO4	uc001pds.4,uc021qos.1	4.272	1.54E-03	4.40E-02
ATP1B3	uc003eug.1,uc011bne.1	1.038	1.61E-03	4.60E-02
ABHD17C	uc002bft.3,uc002bfu.3,uc021ssb.1	1.721	1.62E-03	4.60E-02
ZNF594	uc010cla.1,uc021tol.1	-1.864	1.62E-03	4.60E-02
RALGDS	uc004ccn.3,uc004cco.4,uc004ccp.4,uc004ccq.4,uc004ccr.3,uc004ccs.4,uc004cct.1,uc004ccu.1,uc004ccv.1,uc004ccw.3,uc004ccy.1,uc010nab.3,uc010nac.1,uc011mcv.3,uc011mcw.2,uc011mcx.2	1.329	1.62E-03	4.60E-02
ITM2A	uc004edh.3,uc011mqr.2	1.030	1.62E-03	4.60E-02
DUSP16	uc001ran.2,uc001rao.2	1.337	1.64E-03	4.65E-02
LRIG1	uc003dmw.3,uc003dmx.3,uc010hnz.3,uc010hoa.3,uc011bfu.2	1.050	1.65E-03	4.67E-02
LRP8	uc001cvh.2,uc001cvi.2,uc001cvj.2,uc001cvk.2,uc001cvl.2,uc001cvm.1	1.323	1.65E-03	4.68E-02
IKBKE	uc001hdz.2,uc001hea.2,uc009xbu.2,uc009xbv.2	1.029	1.66E-03	4.68E-02
PIM3	uc003bjb.3,uc011arj.2	1.105	1.66E-03	4.68E-02
INTS7	uc001hiw.2,uc001hix.2,uc001hiy.2,uc009xdb.2,uc010pta.2	1.155	1.67E-03	4.71E-02
PLXND1	uc003emw.2,uc003emx.2,uc011blb.1	3.906	1.68E-03	4.71E-02
PACSIN1	uc003ojo.4,uc003ojp.4	2.409	1.69E-03	4.75E-02
FAM229B	uc003pvs.3	-2.463	1.70E-03	4.77E-02
LINC00565	uc031qnq.1	-1.469	1.71E-03	4.77E-02
AIF1	uc003nuy.3,uc003nva.3,uc010jsy.3,uc031snn.1	-6.840	1.71E-03	4.77E-02
LAIR1	uc002qfk.1,uc002qfl.1,uc002qfm.1,uc002qfn.1,uc002qfo.3,uc010yex.2	-1.307	1.71E-03	4.77E-02
TNFSF9	uc002mfh.2	19.000	1.75E-03	4.89E-02
LOC100996255	uc001zgs.2	-1.498	1.76E-03	4.90E-02
GINS2	uc002fja.3,uc002fjb.2	1.807	1.76E-03	4.90E-02
SLC1A4	uc002sdh.3,uc010ypz.2,uc010yqa.2	3.223	1.77E-03	4.90E-02
ARID5B	uc001jlt.2,uc001jlu.2,uc010qil.2	1.450	1.77E-03	4.90E-02
AMIGO1	uc001dxx.4,uc021org.1	-1.611	1.79E-03	4.94E-02
BTG3	uc002ykk.3,uc002ykl.3	1.251	1.78E-03	4.94E-02
PTPN7	uc001gxl.2,uc001gxm.2,uc001gxn.3,uc001gxo.1,uc010ppw.2,uc010ppx.2,uc010ppy.2	0.932	1.80E-03	4.96E-02
GPR137B	uc001hxq.3	1.499	1.80E-03	4.96E-02
SH3BP5	uc003bzp.2,uc003bzq.2,uc003bzr.2	1.027	1.80E-03	4.96E-02

NFATC2	uc002xwc.4,uc002xwd.4,uc002xwe.4,uc010zyv.3,uc010zyw.3,uc010zyx.3,uc010zyy.3,uc010zyz.3	1.006	1.81E-03	4.97E-02
AF070581	uc004epb.3	3.515	1.82E-03	4.98E-02
