# Supplementary material for: Functional differences between PD-1+ and PD-1- CD4+ effector T cells in healthy donors and patients with glioblastoma multiforme
Source: PLoS One. 2017 Sep 7;12(9):e0181538. doi: 10.1371/journal.pone.0181538 (PMC5589094; doi:10.1371/journal.pone.0181538)
Supplement: S4 Table — Enrichment scores are shown for each group. (PDF) [file pone.0181538.s011.pdf]

S4 Table. DAVID Gene Classification for PD-1^{+} healthy donors. Enrichment scores are shown for each group.

Group ID	Gene Symbol	Gene Name
**Group 1 (2.5886)**	TMX4	thioredoxin-related transmembrane protein 4
	**LAG3**	lymphocyte-activation gene 3
	GPR137B	G protein-coupled receptor 137B
	ITPRIPL1	inositol 1,4,5-triphosphate receptor interacting protein-like 1
	ITGA4	integrin, alpha 4 (antigen CD49D, alpha 4 subunit of VLA-4 receptor)
	B3GNT9	UDP-GlcNAc:betaGal beta-1,3-N-acetylglucosaminyltransferase 9
	PAQR4	progestin and adipoQ receptor family member IV
	GPR56	G protein-coupled receptor 56
	**TNFRSF1B**	tumor necrosis factor receptor superfamily, member 1B
	CD300A	CD300a molecule
	KLRB1	killer cell lectin-like receptor subfamily B, member 1
	SLAMF6	SLAM family member 6
	LGR6	leucine-rich repeat-containing G protein-coupled receptor 6
	ITGAL	integrin, alpha L (antigen CD11A (p180), lymphocyte function-associated antigen 1; alpha polypeptide)
	MCOLN2	mucolipin 2
	SLCO3A1	solute carrier organic anion transporter family, member 3A1
	MS4A1	membrane-spanning 4-domains, subfamily A, member 1
	LAIR2	leukocyte-associated immunoglobulin-like receptor 2
	SLAMF7	SLAM family member 7
	FCRL6	Fc receptor-like 6
	NKG7	natural killer cell group 7 sequence
	**SLAMF1**	signaling lymphocytic activation molecule family member 1
	**PDCD1**	programmed cell death 1
	**IL10RA**	interleukin 10 receptor, alpha
	TSHR	thyroid stimulating hormone receptor
	MRC1	mannose receptor, C type 1
	ADAM8	ADAM metallopeptidase domain 8
	SGPP2	sphingosine-1-phosphate phosphotase 2
	**TNFSF9**	tumor necrosis factor (ligand) superfamily, member 9
	VANGL1	vang-like 1 (van gogh, Drosophila)
	GPR25	G protein-coupled receptor 25
	**TNFRSF18**	tumor necrosis factor receptor superfamily, member 18
	**CD2**	CD2 molecule
	LRFN4	leucine rich repeat and fibronectin type III domain containing 4
	SPPL2A	signal peptide peptidase-like 2A
	**PTGDR**	prostaglandin D2 receptor (DP)
	CD93	CD93 molecule

	REEP3	receptor accessory protein 3
	SEMA4A	sema domain, immunoglobulin domain (Ig), transmembrane domain (TM) and short cytoplasmic domain, (semaphorin) 4A
	SEMA4B	sema domain, immunoglobulin domain (Ig), transmembrane domain (TM) and short cytoplasmic domain, (semaphorin) 4B
	KIAA1324	KIAA1324
	**CD226**	CD226 molecule
	**CLECL1**	C-type lectin-like 1
	SLC35F2	solute carrier family 35, member F2
	**CTLA4**	cytotoxic T-lymphocyte-associated protein 4
	FCRL3	Fc receptor-like 3
	KLRG1	killer cell lectin-like receptor subfamily G, member 1
	CALHM2	calcium homeostasis modulator 2
	GPR68	G protein-coupled receptor 68
	**CD58**	CD58 molecule
	B4GALT5	UDP-Gal:betaGlcNAc beta 1,4- galactosyltransferase, polypeptide 5
	MS4A6A	membrane-spanning 4-domains, subfamily A, member 6A
	NETO2	neuropilin (NRP) and tolloid (TLL)-like 2
	TMEM116	transmembrane protein 116
	GPR183	G protein-coupled receptor 183
	CD84	CD84 molecule
	TSPAN2	tetraspanin 2
	**CXCR5**	chemokine (C-X-C motif) receptor 5
	GLIPR1	GLI pathogenesis-related 1
	CD82	CD82 molecule
**Group 2 (2.4598)**	PRC1	protein regulator of cytokinesis 1
	CEP55	centrosomal protein 55kDa
	NUSAP1	nucleolar and spindle associated protein 1
	NDC80	NDC80 homolog, kinetochore complex component (S. cerevisiae)
	KIF11	kinesin family member 11
	TPX2	TPX2, microtubule-associated, homolog (Xenopus laevis)
	NCAPH	non-SMC condensin I complex, subunit H
**Group 3 (2.3649)**	CDK1	cell division cycle 2, G1 to S and G2 to M
	WEE1	WEE1 homolog (S. pombe)
	BUB1B	budding uninhibited by benzimidazoles 1 homolog beta (yeast)
	NEK3	NIMA (never in mitosis gene a)-related kinase 3
	STK39	serine threonine kinase 39 (STE20/SPS1 homolog, yeast)
	KIF11	kinesin family member 11
	TPX2	TPX2, microtubule-associated, homolog (Xenopus laevis)
**Group 4 (2.3059)**	WEE1	WEE1 homolog (S. pombe)
	KIF19	kinesin family member 19
	MAP4K1	mitogen-activated protein kinase kinase kinase kinase 1
	ACSF2	acyl-CoA synthetase family member 2

	SRXN1	sulfiredoxin 1 homolog (S. cerevisiae)
	TTL	tubulin tyrosine ligase
	EPHA4	EPH receptor A4
	ERN1	endoplasmic reticulum to nucleus signaling 1
	TBK1	TANK-binding kinase 1
	MAP3K8	mitogen-activated protein kinase kinase kinase 8
	EHD4	EH-domain containing 4
	TOR3A	torsin family 3, member A
	UCK2	uridine-cytidine kinase 2
	IKBKE	inhibitor of kappa light polypeptide gene enhancer in B-cells, kinase epsilon
	NEK3	NIMA (never in mitosis gene a)-related kinase 3
	DYRK1B	dual-specificity tyrosine-(Y)-phosphorylation regulated kinase 1B
	FGR	Gardner-Rasheed feline sarcoma viral (v-fgr) oncogene homolog
	STK39	serine threonine kinase 39 (STE20/SPS1 homolog, yeast)
	FGFR2	fibroblast growth factor receptor 2
**Group 5 (0.9631)**	PRSS23	protease, serine, 23
	CTSH	cathepsin H
	GZMH	granzyme H (cathepsin G-like 2, protein h-CCPX)
	CTSC	cathepsin C
**Group 6 (0.4736)**	FBXL8	F-box and leucine-rich repeat protein 8
	WSB2	WD repeat and SOCS box-containing 2
	ASB2	ankyrin repeat and SOCS box-containing 2
	SPSB1	splA/ryanodine receptor domain and SOCS box containing 1
**Group 7 (0.4686)**	BATF	basic leucine zipper transcription factor, ATF-like
	IKZF3	IKAROS family zinc finger 3 (Aiolos)
	RORC	RAR-related orphan receptor C
	HLF	hepatic leukemia factor
**Group 8 (0.3519)**	KLF6	Kruppel-like factor 6
	IKZF3	IKAROS family zinc finger 3 (Aiolos)
	PHF21A	PHD finger protein 21A
	ZBTB32	zinc finger and BTB domain containing 32
	ZNF532	similar to zinc finger protein 347; zinc finger protein 532
**Group 9 (0.2459)**	MAF	v-maf musculoaponeurotic fibrosarcoma oncogene homolog (avian)
	KLF6	Kruppel-like factor 6
	MEOX1	mesenchyme homeobox 1
	SERTAD3	SERTA domain containing 3
	NFATC2	nuclear factor of activated T-cells, cytoplasmic, calcineurin-dependent 2
**Group 10 (0.1544)**	TOX2	TOX high mobility group box family member 2
	BATF	basic leucine zipper transcription factor, ATF-like
	CDCA7	cell division cycle associated 7
	PHTF1	putative homeodomain transcription factor 1

**Group 11 (0.0763)**	ZNF365	zinc finger protein 365
	RNF214	ring finger protein 214
	ZNF831	zinc finger protein 831
	MICAL2	microtubule associated monoxygenase, calponin and LIM domain containing 2
	LIMS3	LIM and senescent cell antigen-like domains 3; LIMS3-LOC440895 read-through
