# Supplementary material for: Functional differences between PD-1+ and PD-1- CD4+ effector T cells in healthy donors and patients with glioblastoma multiforme
Source: PLoS One. 2017 Sep 7;12(9):e0181538. doi: 10.1371/journal.pone.0181538 (PMC5589094; doi:10.1371/journal.pone.0181538)
Supplement: S5 Table — (PDF) [file pone.0181538.s012.pdf]

S5 Table. Gene set enrichment results for PD1 positive and negative Teff from healthy donors (FDR<0.05).

Enriched In	Gene Set	Category (Cell cycle/prolif, Immune system/ signalling, Metabolism, Other)	SIZE	ES	NES	FDR q-val	FWER p-val
PD-1 positive	REACTOME_REGULATION_OF_APOPTOSIS	**CellCycle/Prolif**	56	0.292	2.564	7.09E-04	0.043
	REACTOME_APC_C_CDC20_MEDIATED_DEGRADATION_OF_MITOTIC_PROTEINS	**CellCycle/Prolif**	65	0.268	2.549	7.68E-04	0.048
	NABA_CORE_MATRISOME	**ECM / adhesion / matrix**	274	0.288	5.593	<0.0001	<0.0001
	NABA_ECM_REGULATORS	**ECM / adhesion / matrix**	226	0.305	5.270	<0.0001	<0.0001
	NABA_ECM_AFFILIATED	ECM / adhesion / matrix	164	0.320	4.835	<0.0001	<0.0001
	NABA_ECM_GLYCOPROTEINS	ECM / adhesion / matrix	195	0.285	4.655	<0.0001	<0.0001
	REACTOME_EXTRACELLULAR_MATRIX_ORGANIZATION	**ECM / adhesion / matrix**	83	0.361	3.842	<0.0001	<0.0001
	KEGG_CELL_ADHESION_MOLECULES_CAMS	**ECM / adhesion / matrix**	128	0.266	3.470	<0.0001	<0.0001
	KEGG_REGULATION_OF_ACTIN_CYTOSKELETON	**ECM / adhesion / matrix**	208	0.190	3.176	<0.0001	<0.0001
	KEGG_FOCAL_ADHESION	ECM / adhesion / matrix	194	0.189	3.064	<0.0001	<0.0001
	KEGG_ECM_RECEPTOR_INTERACTION	ECM / adhesion / matrix	82	0.290	3.040	3.06E-05	0.001
	REACTOME_KINESINS	**ECM / adhesion / matrix**	24	0.522	3.030	2.99E-05	0.001
	REACTOME_COLLAGEN_FORMATION	ECM / adhesion / matrix	55	0.330	2.921	4.63E-05	0.002
	REACTOME_DEGRADATION_OF_THE_EXTRACELLULAR_MATRIX	ECM / adhesion / matrix	28	0.460	2.917	4.55E-05	0.002
	NABA_BASEMENT_MEMBRANES	ECM / adhesion / matrix	40	0.394	2.904	6.51E-05	0.003
	PID_INTEGRIN_A9B1_PATHWAY	**ECM / adhesion / matrix**	25	0.473	2.898	6.40E-05	0.003
	PID_INTEGRIN3_PATHWAY	**ECM / adhesion / matrix**	42	0.374	2.850	8.10E-05	0.004
	PID_INTEGRIN1_PATHWAY	ECM / adhesion / matrix	66	0.297	2.811	1.39E-04	0.007
	REACTOME_INTEGRIN_CELL_SURFACE_INTERACTIONS	ECM / adhesion / matrix	78	0.250	2.617	4.31E-04	0.026
	NABA_COLLAGENS	ECM / adhesion / matrix	44	0.320	2.550	7.78E-04	0.048
	REACTOME_GPCR_LIGAND_BINDING	**Immune System / Signalling**	390	0.314	7.335	<0.0001	<0.0001
	NABA_SECRETED_FACTORS	**Immune System / Signalling**	335	0.342	7.246	<0.0001	<0.0001
	REACTOME_CLASS_A1_RHODOPSIN_LIKE_RECEPTORS	**Immune System / Signalling**	290	0.348	6.834	<0.0001	<0.0001
	REACTOME_PEPTIDE_LIGAND_BINDING_RECEPTORS	**Immune System / Signalling**	180	0.360	5.739	<0.0001	<0.0001
	KEGG_CYTOKINE_CYTOKINE_RECEPTOR_INTERACTION	**Immune System / Signalling**	252	0.300	5.452	<0.0001	<0.0001
	REACTOME_G_ALPHA_I_SIGNALLING_EVENTS	Immune System / Signalling	189	0.331	5.271	<0.0001	<0.0001
	REACTOME_G_ALPHA_Q_SIGNALLING_EVENTS	Immune System / Signalling	177	0.283	4.547	<0.0001	<0.0001
	REACTOME_ADAPTIVE_IMMUNE_SYSTEM	**Immune System / Signalling**	495	0.172	4.424	<0.0001	<0.0001
	REACTOME_GASTRIN_CREB_SIGNALLING_PATHWAY_VIA_PKC_AND_MAPK	Immune System / Signalling	198	0.251	4.043	<0.0001	<0.0001
	REACTOME_HEMOSTASIS	**Immune System / Signalling**	443	0.165	3.952	<0.0001	<0.0001
	REACTOME_CHEMOKINE_RECEPTORS_BIND_CHEMOKINES	**Immune System / Signalling**	54	0.427	3.758	<0.0001	<0.0001
	KEGG_COMPLEMENT_AND_COAGULATION_CASCADES	Immune System / Signalling	65	0.396	3.711	<0.0001	<0.0001
	KEGG_AUTOIMMUNE_THYROID_DISEASE	**Immune System / Signalling**	48	0.452	3.689	<0.0001	<0.0001
	REACTOME_DEFENSINS	Immune System / Signalling	43	0.469	3.583	<0.0001	<0.0001
	REACTOME_INNATE_IMMUNE_SYSTEM	Immune System / Signalling	258	0.192	3.570	<0.0001	<0.0001
	REACTOME_G_ALPHA_S_SIGNALLING_EVENTS	Immune System / Signalling	118	0.274	3.476	<0.0001	<0.0001
	KEGG_SYSTEMIC_LUPUS_ERYTHEMAT	Immune System / Signalling	123	0.271	3.443	<0.0001	<0.0001

	OSUS						
	REACTOME_BETA_DEFENSINS	Immune System / Signalling	36	0.479	3.438	<0.0001	<0.0001
	KEGG_CALCIUM_SIGNALING_PATHWAY	**Immune System / Signalling**	172	0.212	3.250	<0.0001	<0.0001
	KEGG_TOLL_LIKE_RECEPTOR_SIGNALING_PATHWAY	Immune System / Signalling	100	0.268	3.167	<0.0001	<0.0001
	KEGG_TYPE_I_DIABETES_MELLITUS	Immune System / Signalling	38	0.430	3.161	<0.0001	<0.0001
	KEGG_MAPK_SIGNALING_PATHWAY	**Immune System / Signalling**	258	0.167	3.114	<0.0001	<0.0001
	REACTOME_SIGNALING_BY_THE_B_CELL_RECEPTOR_BCR	Immune System / Signalling	121	0.238	3.108	<0.0001	<0.0001
	KEGG_CHEMOKINE_SIGNALING_PATHWAY	Immune System / Signalling	184	0.187	2.997	2.92E-05	0.001
	REACTOME_ANTIGEN_PROCESSING_CROSS_PRESENTATION	**Immune System / Signalling**	70	0.313	2.991	2.85E-05	0.001
	KEGG_GAP_JUNCTION	Immune System / Signalling	86	0.274	2.963	2.72E-05	0.001
	KEGG_MATURITY_ONSET_DIABETES_OF_THE_YOUNG	Immune System / Signalling	24	0.507	2.961	2.66E-05	0.001
	REACTOME_PLATELET_ACTIVATION_SIGNALING_AND_AGGREGATION	Immune System / Signalling	195	0.178	2.939	5.20E-05	0.002
	REACTOME_CROSS_PRESENTATION_OF_SOLUBLE_EXOGENOUS_ANTIGENS_ENDOSOMES	Immune System / Signalling	47	0.356	2.938	5.10E-05	0.002
	REACTOME_CYTOKINE_SIGNALING_IN_IMMUNE_SYSTEM	**Immune System / Signalling**	255	0.159	2.923	4.81E-05	0.002
	REACTOME_ACTIVATION_OF_NF_KAPPAB_IN_B_CELLS	Immune System / Signalling	62	0.314	2.922	4.72E-05	0.002
	KEGG_GRAFT_VERSUS_HOST_DISEASE	Immune System / Signalling	32	0.427	2.869	8.37E-05	0.004
	KEGG_INTESTINAL_IMMUNE_NETWORK_FOR_IGA_PRODUCTION	Immune System / Signalling	44	0.357	2.788	1.76E-04	0.009
	KEGG_JAK_STAT_SIGNALING_PATHWAY	Immune System / Signalling	150	0.192	2.772	1.73E-04	0.009
	PID_AP1_PATHWAY	Immune System / Signalling	70	0.279	2.751	1.68E-04	0.009
	PID_PDGFRB_PATHWAY	Immune System / Signalling	128	0.199	2.736	1.84E-04	0.01
	REACTOME_ER_PHAGOSOME_PATHWAY	Immune System / Signalling	56	0.310	2.713	1.98E-04	0.011
	BIOCARTA_INFLAM_PATHWAY	**Immune System / Signalling**	28	0.423	2.684	2.47E-04	0.014
	REACTOME_INTERFERON_GAMMA_SIGNALING	**Immune System / Signalling**	57	0.294	2.662	3.30E-04	0.019
	BIOCARTA_CYTOKINE_PATHWAY	**Immune System / Signalling**	21	0.475	2.653	3.60E-04	0.021
	KEGG_ALLOGRAFT_REJECTION	**Immune System / Signalling**	33	0.384	2.608	4.37E-04	0.027
	REACTOME_BIOLOGICAL_OXIDATIONS	Immune System / Signalling	126	0.197	2.602	4.79E-04	0.029
	REACTOME_AMINE_LIGAND_BINDING_RECEPTORS	**Metabolism**	37	0.454	3.282	<0.0001	<0.0001
	REACTOME_PHASE1_FUNCTIONALIZATION_OF_COMPOUNDS	**Metabolism**	67	0.297	2.876	8.51E-05	0.004
	REACTOME_TRANSMEMBRANE_TRANSPORT_OF_SMALL_MOLECULES	**Metabolism**	402	0.123	2.864	8.24E-05	0.004
	REACTOME_REGULATION_OF_ORNITHINE_DECARBOXYLASE_ODC	**Metabolism**	48	0.336	2.760	1.70E-04	0.009
	KEGG_RETINOL_METABOLISM	**Metabolism**	54	0.303	2.696	2.14E-04	0.012
	REACTOME_NUCLEOTIDE_LIKE_PURINERGIC_RECEPTORS	**Metabolism**	15	0.554	2.633	3.88E-04	0.023
	KEGG_OLFACTORY_TRANSDUCTION	**Other**	377	0.592	13.171	<0.0001	<0.0001
	REACTOME_OLFACTORY_SIGNALING_PATHWAY	**Other**	313	0.621	12.692	<0.0001	<0.0001
	KEGG_NEUROACTIVE_LIGAND_RECEPTOR_INTERACTION	**Other**	267	0.362	6.839	<0.0001	<0.0001
	REACTOME_NEURONAL_SYSTEM	**Other**	273	0.192	3.644	<0.0001	<0.0001
	REACTOME_DEVELOPMENTAL_BIOLOGY	**Other**	382	0.153	3.400	<0.0001	<0.0001
	REACTOME_POTASSIUM_CHANNELS	**Other**	97	0.287	3.274	<0.0001	<0.0001
	REACTOME_AXON_GUIDANCE	Other	242	0.173	3.119	<0.0001	<0.0001
	KEGG_AXON_GUIDANCE	Other	127	0.228	2.974	2.78E-05	0.001

	KEGG_VIRAL_MYOCARDITIS	Other	65	0.310	2.945	2.61E-05	0.001
	KEGG_HYPERTROPHIC_CARDIOMYOPATHY_HCM	Other	82	0.277	2.944	2.55E-05	0.001
	REACTOME_TRANSMISSION_ACROSS_CHEMICAL_SYNAPSES	Other	182	0.187	2.936	5.00E-05	0.002
	KEGG_DILATED_CARDIOMYOPATHY	Other	88	0.269	2.933	4.90E-05	0.002
	KEGG_ENDOCYTOSIS	Other	174	0.190	2.912	6.63E-05	0.003
	REACTOME_NEUROTRANSMITTER_RECEPTOR_BINDING_AND_DOWNSTREAM_TRANSMISSION_IN_THE_POSTSYNAPTIC_CELL	Other	133	0.210	2.814	1.41E-04	0.007
	REACTOME_VOLTAGE_GATED_POTASSIUM_CHANNELS	Other	43	0.342	2.612	4.42E-04	0.027
**PD-1 negative**	REACTOME_P53_DEPENDENT_G1_DNA_DAMAGE_RESPONSE	CellCycle/Prolif	53	-0.307	-2.660	2.95E-04	0.027
	REACTOME_P53_INDEPENDENT_G1_S_DNA_DAMAGE_CHECKPOINT	CellCycle/Prolif	48	-0.318	-2.630	3.55E-04	0.033
	REACTOME_CELL_CYCLE_MITOTIC	CellCycle/Prolif	306	-0.130	-2.629	3.50E-04	0.033
	REACTOME_CELL_CYCLE	CellCycle/Prolif	394	-0.113	-2.622	3.86E-04	0.037
	REACTOME_S_PHASE	**CellCycle/Prolif**	106	-0.222	-2.605	4.39E-04	0.044
	REACTOME_INFLUENZA_LIFE_CYCLE	Immune System / Signalling	132	-0.452	-6.079	<0.0001	<0.0001
	REACTOME_HIV_INFECTION	Immune System / Signalling	187	-0.292	-4.578	<0.0001	<0.0001
	REACTOME_HOST_INTERACTIONS_OF_HIV_FACTORS	Immune System / Signalling	118	-0.314	-4.010	<0.0001	<0.0001
	REACTOME_ANTIGEN_PROCESSING_UBIQUITINATION_PROTEASOME_DEGRADATION	Immune System / Signalling	194	-0.246	-3.999	<0.0001	<0.0001
	REACTOME_HIV_LIFE_CYCLE	Immune System / Signalling	109	-0.316	-3.909	<0.0001	<0.0001
	REACTOME_CLASS_I_MHC_MEDIATED_ANTIGEN_PROCESSING_PRESENTATION	Immune System / Signalling	229	-0.207	-3.590	<0.0001	<0.0001
	REACTOME_SIGNALING_BY_WNT	Immune System / Signalling	61	-0.302	-2.774	1.85E-04	0.015
	REACTOME_DOWNSTREAM_SIGNALING_EVENTS_OF_B_CELL_RECEPTOR_BCR	Immune System / Signalling	92	-0.246	-2.683	2.71E-04	0.024
	REACTOME_METABOLISM_OF_RNA	Metabolism	247	-0.354	-6.427	<0.0001	<0.0001
	REACTOME_METABOLISM_OF_MRNA	Metabolism	204	-0.345	-5.622	<0.0001	<0.0001
	REACTOME_METABOLISM_OF_PROTEINS	Metabolism	418	-0.182	-4.302	<0.0001	<0.0001
	REACTOME_METABOLISM_OF_NON_CODING_RNA	Metabolism	46	-0.409	-3.309	<0.0001	<0.0001
	KEGG_PYRIMIDINE_METABOLISM	Metabolism	93	-0.246	-2.819	1.14E-04	0.009
	REACTOME_PEPTIDE_CHAIN_ELONGATION	Other	83	-0.708	-7.706	<0.0001	<0.0001
	KEGG_RIBOSOME	Other	84	-0.681	-7.363	<0.0001	<0.0001
	REACTOME_INFLUENZA_VIRAL_RNA_TRANSCRIPTION_AND_REPLICATION	Other	99	-0.614	-7.059	<0.0001	<0.0001
	REACTOME_3_UTR_MEDIATED_TRANSLATIONAL_REGULATION	Other	102	-0.591	-6.945	<0.0001	<0.0001
	REACTOME_NONSENSE_MEDIATED_DECAY_ENHANCED_BY_THE_EXON_JUNCTION_COMPLEX	Other	103	-0.564	-6.783	<0.0001	<0.0001
	REACTOME_GENERIC_TRANSCRIPTION_PATHWAY	Other	328	-0.303	-6.336	<0.0001	<0.0001
	REACTOME_SRP_DEPENDENT_COTRANSLATIONAL_PROTEIN_TARGETING_TO_MEMBRANE	Other	106	-0.504	-6.136	<0.0001	<0.0001
	REACTOME_TRANSLATION	Other	142	-0.434	-5.974	<0.0001	<0.0001
	REACTOME_PROCESSING_OF_CAPPED_INTRON_CONTAINING_PRE_MRNA	Other	131	-0.414	-5.516	<0.0001	<0.0001
	REACTOME_MRNA_PROCESSING	Other	151	-0.382	-5.504	<0.0001	<0.0001
	KEGG_SPLICEOSOME	Other	120	-0.409	-5.342	<0.0001	<0.0001
	REACTOME_MRNA_SPLICING	Other	103	-0.427	-5.032	<0.0001	<0.0001
	REACTOME_FORMATION_OF_THE_TER	Other	47	-0.570	-4.739	<0.0001	<0.0001

	NARY_COMPLEX_AND_SUBSEQUENTLY_THE_43S_COMPLEX						
	REACTOME_ACTIVATION_OF_THE_MRNA_UPON_BINDING_OF_THE_CAP_BINDING_COMPLEX_AND_EIFS_AND_SUBSEQUENT_BINDING_TO_43S	Other	54	-0.531	-4.569	<0.0001	<0.0001
	REACTOME_RESPIRATORY_ELECTRON_TRANSPORT_ATP_SYNTHESIS_BY_CHEMIOSMOTIC_COUPLING_AND_HEAT_PRODUCTION_BY_UNCOUPLING_PROTEINS_	Other	78	-0.423	-4.387	<0.0001	<0.0001
	REACTOME_RESPIRATORY_ELECTRON_TRANSPORT	Other	64	-0.438	-4.050	<0.0001	<0.0001
	REACTOME_RNA_POL_II_TRANSCRIPTION	Other	94	-0.346	-3.900	<0.0001	<0.0001
	REACTOME_TCA_CYCLE_AND_RESPIRATORY_ELECTRON_TRANSPORT	Other	114	-0.310	-3.887	<0.0001	<0.0001
	REACTOME_TRANSCRIPTION	Other	190	-0.238	-3.701	<0.0001	<0.0001
	REACTOME_MRNA_SPLICING_MINOR_PATHWAY	Other	42	-0.458	-3.473	<0.0001	<0.0001
	KEGG_OXIDATIVE_PHOSPHORYLATION	Other	114	-0.270	-3.441	<0.0001	<0.0001
	KEGG_HUNTINGTONS_DISEASE	Other	170	-0.226	-3.423	<0.0001	<0.0001
	REACTOME_DNA_REPAIR	Other	103	-0.285	-3.408	<0.0001	<0.0001
	KEGG_PARKINSONS_DISEASE	Other	111	-0.274	-3.398	<0.0001	<0.0001
	REACTOME_TRANSPORT_OF_MATURE_TRANSCRIPT_TO_CYTOPLASM	Other	49	-0.406	-3.390	<0.0001	<0.0001
	KEGG_RNA_DEGRADATION	Other	57	-0.370	-3.333	<0.0001	<0.0001
	REACTOME_NUCLEOTIDE_EXCISION_REPAIR	Other	48	-0.395	-3.270	<0.0001	<0.0001
	REACTOME_CLEAVAGE_OF_GROWING_TRANSCRIPT_IN_THE_TERMINATION_REGION_	Other	39	-0.427	-3.179	<0.0001	<0.0001
	REACTOME_REGULATION_OF_MRNA_STABILITY_BY_PROTEINS_THAT_BIND_AU_RICH_ELEMENTS	Other	80	-0.301	-3.145	<0.0001	<0.0001
	KEGG_RNA_POLYMERASE	Other	29	-0.480	-3.094	<0.0001	<0.0001
	REACTOME_TRANSCRIPTION_COUPLED_NER_TC_NER	Other	43	-0.391	-2.976	<0.0001	<0.0001
	KEGG_NUCLEOTIDE_EXCISION_REPAIR	Other	42	-0.383	-2.945	1.63E-05	0.001
	KEGG_AMINOACYL_TRNA_BIOSYNTHESIS	Other	40	-0.389	-2.928	3.06E-05	0.002
	REACTOME_RNA_POL_III_TRANSCRIPTION_INITIATION_FROM_TYPE_3_PROMOTER	Other	26	-0.480	-2.925	3.00E-05	0.002
	REACTOME_TRNA_AMINOACYLATION	Other	41	-0.391	-2.916	4.36E-05	0.003
	REACTOME_DESTABILIZATION_OF_MRNA_BY_AUF1_HNRNP_D0	Other	50	-0.343	-2.882	8.65E-05	0.006
	REACTOME_ANTIVIRAL_MECHANISM_BY_IFN_STIMULATED_GENES	Other	63	-0.313	-2.878	8.48E-05	0.006
	REACTOME_RNA_POL_III_TRANSCRIPTION	Other	33	-0.419	-2.869	9.64E-05	0.007
	KEGG_UBIQUITIN_MEDIATED_PROTEOLYSIS	Other	130	-0.211	-2.861	9.46E-05	0.007
	REACTOME_AUTODEGRADATION_OF_THE_E3_UBIQUITIN_LIGASE_COP1	Other	47	-0.358	-2.842	1.20E-04	0.009
	REACTOME_MRNA_3_END_PROCESSING	Other	30	-0.438	-2.839	1.18E-04	0.009
	REACTOME_MITOCHONDRIAL_PROTEIN_IMPORT	Other	48	-0.349	-2.824	1.16E-04	0.009
	REACTOME_RNA_POL_II_PRE_TRANSCRIPTION_EVENTS	Other	55	-0.319	-2.786	1.50E-04	0.012
	REACTOME_TRANSPORT_OF_MATURE_MRNA_DERIVED_FROM_AN_INTRONLESS_TRANSCRIPT	Other	31	-0.406	-2.681	2.67E-04	0.024
	REACTOME_FORMATION_OF_RNA_POL	Other	39	-0.351	-2.617	3.91E-04	0.038

	_II_ELONGATION_COMPLEX_						
	REACTOME_SYNTHESIS_OF_DNA	Other	90	-0.235	-2.615	3.96E-04	0.039
	KEGG_PROTEASOME	Other	44	-0.333	-2.604	4.33E-04	0.044
	REACTOME_LATE_PHASE_OF_HIV_LIFE_CYCLE	Other	96	-0.328	-3.747	<0.0001	<0.0001
	REACTOME_AUTODEGRADATION_OF_CDH1_BY_CDH1_APC_C	Other	56	-0.342	-3.003	<0.0001	<0.0001
	REACTOME_VIF_MEDIATED_DEGRADATION_OF_APOBEC3G	Other	48	-0.359	-2.958	<0.0001	<0.0001
	REACTOME_CDK_MEDIATED_PHOSPHORYLATION_AND_REMOVAL_OF_CDC6	Other	46	-0.358	-2.946	1.66E-05	0.001
	REACTOME_INTERACTIONS_OF_VPR_WITH_HOST_CELLULAR_PROTEINS	Other	31	-0.420	-2.756	2.05E-04	0.017
	REACTOME_DEADENYLATION_DEPENDENT_MRNA_DECAY	Other	42	-0.354	-2.729	2.49E-04	0.021
	REACTOME_CDT1_ASSOCIATION_WITH_THE_CDC6_ORC_ORIGIN_COMPLEX	Other	54	-0.308	-2.691	2.80E-04	0.024
	REACTOME_SCF_BETA_TRCP_MEDIATED_DEGRADATION_OF_EMI1	Other	49	-0.319	-2.688	2.75E-04	0.024
