# Supplementary material for: Functional differences between PD-1+ and PD-1- CD4+ effector T cells in healthy donors and patients with glioblastoma multiforme
Source: PLoS One. 2017 Sep 7;12(9):e0181538. doi: 10.1371/journal.pone.0181538 (PMC5589094; doi:10.1371/journal.pone.0181538)
Supplement: S6 Table — (PDF) [file pone.0181538.s013.pdf]

**S6 Table.** Data for patients used in this study.

| Glioma (G) | Age | Sex | Diagnosis | WHO grade | Previous Treatments | Time from First Diagnosis/MRI to Tumor Resection | | | | Ki67 | IDH1 R132H mutation | MGMT promoter methylation |
|---|---|---|---|---|---|---|---|---|---|---|---|---|
| | | | | | | **Years** | **Months** | **Weeks** | **Days** | | | |
| G1 | 45-49 | F | Glioblastoma Multiforme | IV | Dexamethosone | | 4 | | | 10% | negative | unmethylated |
| G2 | 65-69 | F | Glioblastoma Multiforme | IV | Dexamethosone | | | | 4 | 40% | negative | partially methylated |
| G3 | 60-64 | M | Glioblastoma Multiforme | IV | Dexamethasone | | | | 3 | 80% | negative | unmethylated |
| G4 | 30-34 | F | Secondary Glioblastoma Multiforme | IV | Dexamethasone, Avastin | | | | N/A | 10-20% | | |
| G5 | 70-74 | F | Glioblastoma Multiforme | IV | Dexamethasone | | 10 | | | 20% | not recorded | methylated |
| G6 | 60-64 | M | Glioblastoma Multiforme with Oligodendroglial Component | IV | Dexamethasone | | | | 3 | >25 % | | |
| G7 | 45-49 | M | Glioblastoma Multiforme | IV | Dexamethasone | | 1 | | | >15 % | | |
| G8 | 60-64 | M | Glioblastoma Multiforme | IV | Dexamethasone | | | 1 | | >10 % | not recorded | unmethylated |
| G9 | 60-64 | M | Glioblastoma Multiforme | IV | Dexamethasone | | | | 10 | >25 % | | |
| G10 | 45-49 | M | Glioma with Prominent Oligodendrogliomatous Differentiation | III | Dexamethasone | | | 2 | | 12% | positive | not recorded |
| G11 | 30-34 | F | Anaplastic Oligodendroglioma | III | Dexamethasone | | | | 16 | 20% | positive | methylated |
| G12 | 60-64 | F | Anaplastic Oligodendroglioma | III | Dexamethasone | 12 | | | | 20% | positive | methylated |
| G13 | 45-49 | M | Astrocytoma | II | Dexamethosone | | | | 5 | <4% | positive | methylated |
| G14 | 55-59 | M | Oligoastrocytoma | II | Dexamethasone | 14 | | | | <3% | positive | methylated |
| G15 | 40-44 | M | Oligodendroglioma | II | Dexamethasone | | 3 | | | 5% | positive | not recorded |
| G16 | 55-59 | F | Oligodendrogli | II | Dexamethasone | | | 6 | | NR | not | not |

| | | | | | | | | | | | | |
|---|---|---|---|---|---|---|---|---|---|---|---|---|
| | | | oma | | | | | | | | recorded | recorded |
| G17 | 35-39 | F | Oligoastrocytoma | II | Dexamethasone | | | 6 | | 4-6% | negative | partially methylated |
| G18 | 35-39 | M | Astrocytoma | II | Dexamethasone | | | | 6 | 5-6% | | |
| G19 | 60-64 | M | Glioblastoma Multiforme | IV | Dexamethosone | | | 2 | | >50% | not recorded | methylated |
| G20 | 55-59 | M | Glioblastoma Multiforme | IV | Dexamethasone | | 1 | | | 20% | not recorded | methylated |
| G21 | 45-49 | F | Glioblastoma Multiforme | IV | Dexamethasone | | | | 1 | 20% | negative | methylated |
| G22 | 45-49 | M | Glioblastoma Multiforme | IV | Dexamethasone | | | 1 | | 10% | not recorded | unmethylated |
| G23 | 50-54 | F | Glioblastoma Multiforme | IV | Dexamethasone | | | | 5 | 15% | negative | unmethylated |
| G24 | 70-74 | M | Gliosarcoma | IV | Dexamethasone | | | 2 | | 10% | not recorded | not recorded |
| G25 | 75-79 | M | Glioblastoma Multiforme | IV | Dexamethasone | | | | 1 | >20% | negative | partially methylated |
| G26 | 75-79 | F | Glioblastoma Multiforme | IV | Dexamethasone | | 2 | | | highly elevated | negative | unmethylated |
| G27 | 15-19 | F | Glioblastoma Multiforme | IV | N/A | | | 1 | | 40% | negative | unmethylated |
| G28 | 70-74 | F | Glioblastoma Multiforme | IV | Dexamethasone | | | | 2 | 25% | negative | methylated |
| G29 | 80-84 | F | Glioblastoma Multiforme | IV | Dexamethasone | | | | 3 | 15% | negative | unmethylated |
| G30 | 60-64 | M | Glioblastoma Multiforme | IV | Prednisone | | | | 1 | 15% | positive | methylated |
| G31 | 60-64 | M | Glioblastoma Multiforme | IV | Dexamethasone | | | | 1 | 20% | negative | methylated |
| G32 | 65-69 | M | Glioblastoma Multiforme | IV | Dexamethasone | | 1 | | | 40% | not recorded | unmethylated |
| G33 | 55-59 | M | Glioblastoma Multiforme | IV | Dexamethasone | | | | 6 | 20% | negative | unmethylated |
| G34 | 65-69 | M | Glioblastoma Multiforme | IV | Dexamethasone | | 1 | | | >60% | not recorded | methylated |
| G35 | 50-54 | F | Glioblastoma Multiforme | IV | Dexamethasone | | 1 | | | 5% | not recorded | partially methylated |
| G36 | 55-59 | M | Recurrent Glioblastoma Multiforme | IV | Dexamethasone | | | | N/A | NR | | |
| G37 | 70-74 | M | Glioblastoma | IV | Dexamethasone | | | 1 | | NR | not | unmethyla |

| | | | | | | | | | | | | |
|---|---|---|---|---|---|---|---|---|---|---|---|---|
| | | | Multiforme | | | | | | | | recorded | ted |
| G38 | 45-49 | M | Glioblastoma Multiforme | IV | Dexamethasone | | | 5 | | 40% | negative | unmethylated |
| G39 | 65-69 | F | Glioblastoma Multiforme | IV | Dexamethasone | | | | 3 | 40% | negative | methylated |
| G40 | 80-84 | M | Glioblastoma Multiforme | IV | N/A | | | 1 | | 15% | positive | methylated |
| | | | | | | | | | | | | |
| BT 213 | 79 | F | Glioblastoma | IV | None (Keppra) | | | | 5 | "scattered mitotic figures" | negative | unmethylated |
| BT 215 | 76 | F | Glioblastoma | IV | Dexamethasone | | | | 8 | "numerous mitotic figures" | negative | methylated |
| BT 219 | 56 | F | Anaplastic Oligoastrocytoma | III | Dexamethasone (and Keppra) | | | | 3 | 25-30% | negative | partially methylated |
| BT 220 | 63 | M | Recurrent Residual Glioblastoma | IV | Partial brain XRT + dexamethasone (and Keppra) | | 5 | | | 10-15% | negative | partially methylated |
| BT 223 | 84 | F | Glioblastoma | IV | Dexamethasone (and Keppra) | | 2 | | | >30% | negative | methylated |
| BT 228 | 40 | F | Glioblastoma | IV | Dexamethasone* | | | | 12* | >25% | negative | methylated |
| BT 229 | 61 | M | Glioblastoma | IV | Dexamethasone (and Keppra) | | | | 8 | >10% | negative | methylated |

*Note: 10 years prior, oligoastrocytoma (WHO grade 2) in same location (cerebellum) treated with surgery, temozolomide, radiation therapy, and dexamethasone.
