# Supplementary material for: Functional differences between PD-1+ and PD-1- CD4+ effector T cells in healthy donors and patients with glioblastoma multiforme
Source: PLoS One. 2017 Sep 7;12(9):e0181538. doi: 10.1371/journal.pone.0181538 (PMC5589094; doi:10.1371/journal.pone.0181538)
Supplement: S7 Table — (PDF) [file pone.0181538.s014.pdf]

S7 Table. Curated exhaustion and T cell specific gene signatures from the literature.

Gene Set	Description	Reference	Genes
**Th17 Core 1**	Core signature of memory Th17 cells	Ramesh, 2014	IL17RE, PIK3R6, HLF, CCR6, RORC, CTSH, LRP12, CHN1, HPGD, ADAM12, PTPN13
**Th17 cytokines**	Cytokines secreted by Th17 cells.	Ciofani, 2012	IL23R, IL9, IL17R, IL17A, IL24, IL12RB1, IL17F, LIF, IL10, TNFRSF8, IL21, IL1R1
**Th17 core**	Combinatorial core of transcription factor targets involved in Th17 cell specification.	Ciofani, 2012 Figure 6D	STAT3, IRF4, RORC, MAF, BATF, FOSL2, CYSLTR2, CCL5, HIF1A, IL18RAP, IL17A, IL17F, CCL20, CCR8, IL2, CXCR3, TBX21, CCL4, CCL3, CCR6, IL1R1, GPR15, IL18R1, IL21RB1, IL7R, IL2RA, LTB4R1, IL12RB2, LTB, IL10RA, SMAD3, IL23A, CCR7, IL24, IL4RA, IL21, CXCL10, IL6RA, IL16
**CD8 Blood**	Cell type specific gene sets were identified, including for CD8 T cells	Palmer, 2006	CD8B1, CD8A, CCL5, D12S2489E, PHEMX, KLRG1, S100B, KLRC1, ADRB2, CST7, GZMH, Gzmc, C1orf21, IL2RB, DUSP2, GPR56, CCL4L, ZNF145, FCGBP, TBX21, PRF1, GNLY
**Th1**	Th1-specific gene signature derived from C57BL/6 mice.	Wei, 2011	See Supplementary Table 4 of Wei *et al.*
**Th2**	Th2-specific gene signature derived from C57BL/6 mice.		
**iTreg**	iTreg-specific gene signature derived from C57BL/6 mice.		
**IFN-responsive**	Exhaustion-specific genes associated with CD4+ T cell dysfunction during chronic infection in mice	Crawford, 2014 Figure 3C	MX2, MX1, ISG20, ISG15, IRF7, IFRD1, IFITM3, IFITM1 IFIT3, IFIT1, IFIH1, IFI44, IFI30, AIM2
**Co-inhibitory exhaustion**		Crawford, 2014 Supplemental Table 2	BTLA, CD160, CD200, CD200R1, CD200R1L, CD244, CD274, CTLA4, HAVCR2, LAG3, LAIR1, LILRB4, PDCD1, PDCD1LG2, PILRA, SIRPB1, TIGIT
**Core exhaustion**		Crawford, 2014 Supplemental	ARAP2, CCNA2, CCNB2, CCNYL1, CD86, CENPE, CRLF3, DEPDC1, DPH5, DTL, ENTPD5, EOMES, FAM105A, GPI, GPR141, GRAP2, HSPA4L, IDH2,

		Table 3	IFI44, IFIH1, IRF4, ISG20, KIF15, KLHL6, KLHL7, LAG3, MKI67, MLF1, MLKL, MX1, NAB1, NAP1L3, NCAPG, NLK, NSMAF, NUDT15, PLOD2, PON3, PRR11, RCBTB1, RSAD2, SATB1, SCARNA17, SETBP1, SH2D1A, SNORD35B, TAF1A, TINF2, TMEM140, TMEM2, TNFSF4, TPX2, TXNIP, USP18
**Anergic**	Differentially expressed genes found in early anergic T cell clones (A.E7 $CD4^{+}$)	Safford, 2005 Table 1	EGR2, ETV6, FOXP1, HLF, IRF4, JARID2, NFATC1, NOTCH1, NR4A2, NR4A3, ZFP36L1, ACTN4, AGT, ANGPTL2, ANKRD28 ANP32A, RCBTB1, S100A5, SFRP4, SOCS4, GCH1, KIFC3, PFKP, PLA2G10, ARFIP1, BNIP3, CCRN4L, DTNA, CCL1, CCL3, CSF1, TNFSF11, TNFSF9, FBXO34, GADD45B, HEBP2, DDR1, FYN, JAK3, ADORA2A, ADORA2B, F2R, GABRA4, KCNJ11, KCNK5, KCNQ5, CASP4, CTSE, FURIN, TINAG, GGA2, SLC29A3, STX11, CDC14A, DUSP6, HIF1AN, HSPA1A, IER3, JUP, LAG3, LRRC3, MARCH2, MMD, MYH14, MYL7, MYO1C, MYO1E, NDRG1, OAZ3, AGAP1, TEKT2, TNFRSF19, TNFRSF4, RNF19A, SOCS6, KIF15, CLEC4E, CD40LG, MPZL2, ADGRE5, LPAR4, SRGN
**Activated Treg (up)**	Genes identified by RNAseq in activated and unactivated Tregs ($CD4^{+}CD25^{hi}$) and Th ($CD4^{+}CD25^{-}$)	Birzele, 2011	Table S1, Treg specific, >2FC CD25 activated v unactivated
**Activated Treg (down)**			Table S1, Treg specific, <2FC CD25 activated v unactivated
**Activated Teff (up)**			Table S1, Teff specific, >2FC CD4 activated v unactivated
**Activated Teff (down)**			Table S1, Teff specific, <2FC CD4 activated v unactivated
**PD-1^{hi} up**	Sorted CD8 CCR7lo PD1hi and PD1lo and naïve cells from humans were profiled with microarrays (Table I and Table II)	Duraiswamy, 2011	CD28, CD27, CTLA4, RGS1, SIRPG, MEGF6, PASK, ZNF512B, CXCR6 CXCR4, GZMK, ITM2A, SGPP2, TTC9, MBOAT1
**PD-1^{hi} down**			SH2D1B, TRA/TRD, LYN, LAT2, RAP2A, VAV3, KIR3DL2, KLRC3, KIR3DL1/KIR3DS1, KIR2DL2/3, KIR2DS1/2/4/5, KLRC1/2, KLRF1, KLRC4, IR2DS3, KLRD1, KIR2DL1, KIR2DL4, NCR1, KIR2DL5A,

			NCAM1, ITGAM, PALLD, ITGAX, IKZF2, KLF11, ZNF683, TFCP2L1, METTL7A, GNLY, GZMB, MYO6, GOLIM4, IL8RB, IL7, CMKLR1, CTBP2 BOK, RASSF4, PDGFD, GPR56, HDGFRP3, ADRB1, PRSS23, GOLM1, GLB1L2, TM6SF1, RHOBTB3, SYNGR1, OSBPL5, TSPAN2, TMCC3, LILRB1, DOCK5, ARRB1, PTGDS FCGR3B, CES1, BNC2, SSX2IP, MXRA7
**TF exhaustion**	Gene sets associated with CD8 T cell dysfunction in acute versus chronic CMV infection in mice. Set identified through network analysis of transcription factors associated with Tbet.	Doering, 2012	See Supplemental Table 7, 242 gene signature of transcription factor 'neighbors' in acute and chronic networks
**Exhausted up, memory down**	Gene sets associated with progressive stimulation of CD8 T cells with antigen, identified by comparing CD8 from primary to quaternary antigen exposure.	Wirth, 2010 STable 6 blue module	FIGNL1, CTLA4, PLSCR1, ART3, PRC1, TOP2A, PLK4, MKI67, HIST1H2AE, CD160, EOMES, PGLYRP1, CDC6, MYB, CKS2
**Exhausted up, memory up**		Wirth, 2010 STable 6 orange module	ADGRG1, CCL3, CCL4, CD244, ENTPD1, FGL2, GPD2, GZMA GZMB, GZMK, IRF4, LAG3, LGALS3, MAD2L1, NR4A2, PBX3 PDCD1, PERP, PON2, PRDM1, RGS16, WBP5
**Exhausted down, memory down**		Wirth, 2010 STable 6 yellow module	ARHGAP1, ATP6V0B, CCR7, CEBPB. CRLF3, CTSW, DGKA, DUSP1, DUSP12, EEF1D, EEF2, ELOVL5, EPHB4, EVL, FOSB, GARS, GM2A, GZMM, IL7R, IMPDH2, KCNN4, KCTD12, KLF3, LEF1, LGALS13 LTB, MBP, MRPL13, PDLIM1, PIK3CD, PLAC8, PRG2, PSMD1, RPL10A, RPL13, RPL8, RPLP1, RPN2, RPS3, RPS5, SELL, SLC3A2, SMAD1, SMAD7

**Repeat antigen exposure, up**	Gene sets associated with progressive stimulation of CD8 T cells with antigen, compared quaternary to primary antigen exposure.	Wirth, 2010 Table S5, >2FC	ALCAM, ANXA1, ARHGEF12 ,BHLHE40 ,BHLHE40, CAPG, CAPN2, CARNS1, CCDC109B, CCR5, CD244, CD80, CDC20B, CDHR1,CHN2, CMKLR1, CRYBG3, CX3CR1, DDX28, DENND5A, EMP1, ERN1, ESM1, FASLG, FBXL2,FCGR2B, GABARAPL1, GALNT3,GNA15, GSAP,GSTM5,GZMB,HAVCR2, IFIT2, IL12RB2,IL18RAP,IL1RL1, IL2RA,INPP4A,ITGAM,KCNJ8,KLRC2, KLRC3,KLRG1, LAMC1,LONRF3, LPIN1,LPP, MBD3L2, MMP25, MS4A4A, MT2A, MYL10, NEBL,OSBPL3, PIK3AP1,PLK3, PLSCR4,REEP5,RNF216,RORA, S1PR5, SAR1B, SETBP1,SLAMF1,SLAMF7, SPATS2,SRXN1, SYTL3,TCF4, TCF7L2, TEF,TMBIM1, TMEM97, TSPAN2, TTC39C, ZEB2
